# Emergence of Noise-Induced Oscillations in the Central Circadian Pacemaker

**DOI:** 10.1371/journal.pbio.1000513

**Published:** 2010-10-12

**Authors:** Caroline H. Ko, Yujiro R. Yamada, David K. Welsh, Ethan D. Buhr, Andrew C. Liu, Eric E. Zhang, Martin R. Ralph, Steve A. Kay, Daniel B. Forger, Joseph S. Takahashi

**Affiliations:** 1 Department of Neuroscience, University of Texas Southwestern Medical Center, Dallas, Texas, United States of America; 2 Department of Neurobiology and Physiology, Northwestern University, Evanston, Illinois, United States of America; 3 Department of Psychology, University of Toronto, Toronto, Ontario, Canada; 4 Department of Mathematics, University of Michigan, Ann Arbor, Michigan, United States of America; 5 Department of Cell and Developmental Biology, University of California, San Diego, La Jolla, California, United States of America; 6 Department of Psychiatry, University of California, San Diego, La Jolla, California, United States of America; 7 Veterans Affairs San Diego Healthcare System, San Diego, California, United States of America; 8 Genomics Institute of Novartis Research Foundation, San Diego, California, United States of America; 9 Center for Biological Timing and Cognition, University of Toronto, Toronto, Ontario, Canada; 10 Center for Computational Medicine and Bioinformatics, University of Michigan, Ann Arbor, Michigan, United States of America; 11 Howard Hughes Medical Institute, University of Texas Southwestern Medical Center, Dallas, Texas, United States of America; Howard Hughes Medical Institute/Stanford University, United States of America

## Abstract

*Bmal1* is an essential transcriptional activator within the mammalian circadian clock. We report here that the suprachiasmatic nucleus (SCN) of *Bmal1*-null mutant mice, unexpectedly, generates stochastic oscillations with periods that overlap the circadian range. Dissociated SCN neurons expressed fluctuating levels of PER2 detected by bioluminescence imaging but could not generate circadian oscillations intrinsically. Inhibition of intercellular communication or cyclic-AMP signaling in SCN slices, which provide a positive feed-forward signal to drive the intracellular negative feedback loop, abolished the stochastic oscillations. Propagation of this feed-forward signal between SCN neurons then promotes quasi-circadian oscillations that arise as an emergent property of the SCN network. Experimental analysis and mathematical modeling argue that both intercellular coupling and molecular noise are required for the stochastic rhythms, providing a novel biological example of noise-induced oscillations. The emergence of stochastic circadian oscillations from the SCN network in the absence of cell-autonomous circadian oscillatory function highlights a previously unrecognized level of circadian organization.

## Abstract 2

Computational modeling and experimentation explain how intercellular coupling and intracellular noise can generate oscillations in a mammalian neuronal network even in the absence of cell-autonomous oscillators.

## Author Summary

The suprachiasmatic nucleus (SCN) is the master circadian pacemaker in mammals that controls and coordinates physiological processes in a daily manner. The SCN is composed of a network of cells, with each cell acting as an autonomous oscillator. In isolated individual cells, timekeeping is not precise because of the inherent randomness in the biochemical reactions within each cell, involving its core clock components. However, in the SCN network, precise rhythms can emerge because of intercellular coupling. In this article, we study a loss-of-function mutation of BMAL1, a core clock component, which eliminates timekeeping in isolated cells. Surprisingly, in both experiments and mathematical simulations, we find that noisy rhythms emerge from the SCN network even in the presence of this BMAL1 mutation. This random yet coordinated timekeeping has not been observed in previous modeling and experimental work and indicates that a network of cells can utilize noise to help compensate for loss of a physiological function. In normal function, the SCN network mitigates any variability observed in individual cellular rhythms and produces a precise and rhythmic network timekeeping signal. When the individual cells are no longer rhythmic, the coupling pathways within the SCN network can propagate stochastic rhythms that are a reflection of both feed-forward coupling mechanisms and intracellular noise. Thus, in a manner analogous to central pattern generators in neural circuits, rhythmicity can arise as an emergent property of the network in the absence of component pacemaker or oscillator cells.

## Introduction

Mammalian circadian clocks are cell autonomous and self-sustained within the central pacemaker, the suprachiasmatic nucleus (SCN), as well as within peripheral tissues and fibroblasts [1]–[4]. Circadian rhythms are generated at the molecular level by an autoregulatory transcriptional and translational feedback loop [5],[6]. The bHLH-PAS proteins, CLOCK (*Clock* RefSeq: NM_007715) and BMAL1 (*Arntl* RefSeq: NM_007489), form an activator complex that drives transcription of the *Per* (RefSeq: *Per1* NM_011065 and *Per2* NM_011066) and *Cry* (RefSeq: *Cry1* NM_007771 and *Cry2* NM_009963) genes [7]–[9]. PER and CRY proteins then form repressor complexes and translocate back to the nucleus to inhibit their own transcription [10],[11]. Previous work has demonstrated that *Bmal1* plays an essential role in normal circadian clock function—its inactivation leads to a loss of circadian rhythmicity at the behavioral level [7]. To understand the role of *Bmal1* at a more mechanistic level, we have analyzed the effects of a *Bmal1* loss-of-function mutation (*Bmal1* ^−/−^) on cell- and tissue-autonomous circadian rhythms in the SCN and peripheral tissues. We report here an unexpected stochastic rhythmicity in the SCN of *Bmal1*-deficient mice, which appears as an emergent network property of the SCN.

The roles of intercellular coupling and molecular noise in circadian clock function have been analyzed using mathematical models [12]–[15]. Previous work on modeling the role of coupling within the SCN [16]–[22] and in populations of coupled oscillators [23]–[25] highlights two key ideas: (1) Intercellular coupling can induce rhythmicity in a population of damped oscillators [26]—a prediction that has recently been validated in some [20],[21] but not all [27] modeling studies, and (2) intercellular coupling can synchronize and improve the precision of noisy intracellular oscillators [27]–[30]. More recent modeling studies have emphasized the importance of molecular noise [30]–[35] and its contribution to the generation of single-cell rhythmicity [31],[32],[34],[36]. Importantly, the combination of both intercellular coupling and molecular noise has not previously been shown to generate oscillations in the absence of component oscillatory elements.

In the SCN, the nature and dynamics of the coupling network itself remain largely undefined. Vasoactive intestinal polypeptide (VIP) (*Vip* RefSeq: NM_011702) and its receptor *Vipr2* (RefSeq: NM_009511) are critical signaling elements within the coupling pathway in the SCN [37],[38]. However, it is not known whether the coupling pathway itself may contribute to temporal dynamics and, if so, what its kinetics might be. We report here a mathematical model that incorporates both intercellular coupling and molecular noise, and we show that the interplay between the two can generate oscillations in the SCN neuronal network, even in the absence of a functional circadian clock in individual cells. Our results suggest that the SCN network itself in conjunction with the acute induction pathways for *Per1* and *Per2* can generate quasi-circadian oscillations.

## Results

### Rhythmic PER2::LUC Output in *Bmal1* ^−/−^ SCN Explants

**Figure 1:**
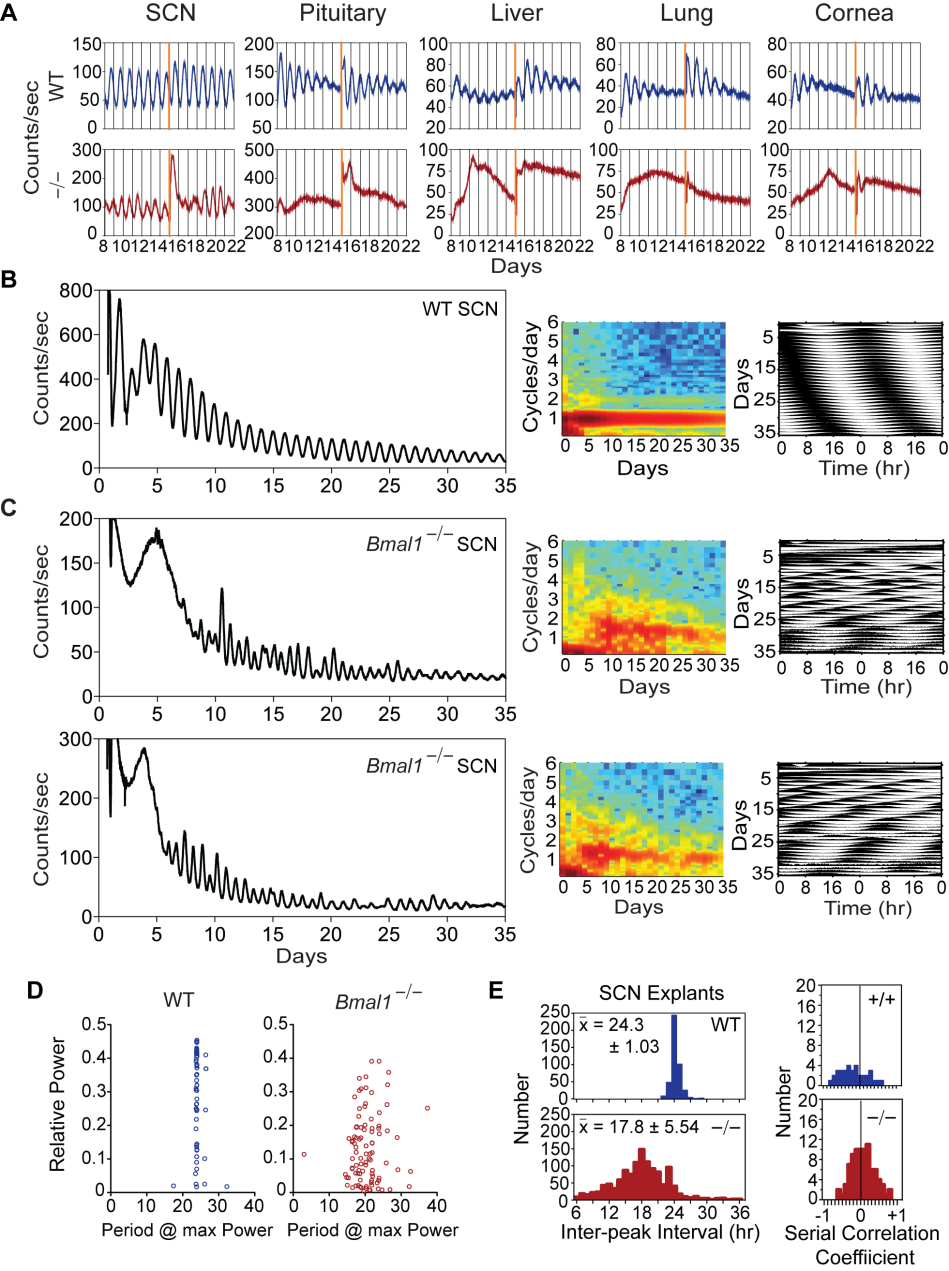
Effects of *Bmal1* mutation on PER2::LUC bioluminescence rhythms. (A) Representative records of PER2::LUC bioluminescence from various tissue explants from wild type (WT) and homozygous *Bmal1*-null mutant (^−/−^) mice. Mice were kept in a light-dark cycle (12 h light, 12 h dark) for approximately 2 wk, then released into constant darkness. The tissue explants were dissected (day 0) and immediately cultured for recording. Data are shown following a medium change (day 8); another medium change occurred at day 15. (B and C) Detailed view of PER2::LUC bioluminescence from SCN explants of WT (B) and *Bmal1* ^−/−^ mice (C). Records begin on the day of culture (day 0) and are “raw” LumiCycle bioluminescence recordings that are not normalized nor corrected for baseline drift. All SCN explants show persistent PER2::LUC rhythms for >35 d (left). FFT spectrograms of the baseline-subtracted records (middle) show a tightly regulated frequency (cycles per day) for the WT SCN; however, more variable frequency components are observed in the *Bmal1* ^−/−^ SCN. Double-plotted raster plots (right) illustrate stable PER2::LUC rhythms in the WT SCN and instability of the rhythms in *Bmal1* ^−/−^ SCN. (D) FFT spectral analysis for PER2::LUC rhythms from SCN explants. Period values with the maximum spectral power were determined for WT and *Bmal1* ^−/−^ SCN explants using FFT spectral analysis (see Methods under Single-Cell Imaging Data Analysis). Each data point represents the maximum frequency component in a 10-d epoch of data. *Bmal1* ^−/−^ SCN explants showed high variability in period length (average = 20.51±4.39 SD h) compared to WT SCN explants (average period = 24.16±0.83 SD h). (E) Histograms of inter-peak intervals for the PER2::LUC rhythmic expression patterns (left) and serial correlation coefficient (*r_s_*, right) of successive inter-peak intervals. The *Bmal1* ^−/−^ SCN explants show a significantly shorter average inter-peak interval and a much broader distribution compared to WT SCNs. Histograms represent 433 inter-peak intervals from 23 WT SCN explants and 1,239 intervals from 19 *Bmal1* ^−/−^ SCN explants. *r_s_* estimates were calculated from successive 7 to 10 inter-peak interval epochs. Histograms represent 36 *r_s_* estimates from 14 WT SCN explants and 85 *r_s_* estimates from 16 *Bmal1* ^−/−^ SCN explants. The average serial correlation coefficient for WT SCN explants was negative (*r_s_* = −0.17, *p*<0.01) as would be expected from a circadian pacemaker-driven process. However, the coefficient for the *Bmal1* ^−/−^ SCN was slightly positive (*r_s_* = 0.07, *p*<0.05).

*Bmal1* is the only known clock gene whose loss-of-function leads to complete loss of circadian rhythmicity in wheel-running behavior [7]. In addition, circadian rhythms of *Per1* and *Per2* mRNA expression are disrupted in the SCN of *Bmal1* ^−/−^ mice [7]. To analyze the role of *Bmal1* in cell- and tissue-autonomous circadian properties in central and peripheral oscillators, we measured PER2::LUC bioluminescence rhythms in various tissue explants from wild type (WT; *Bmal1* ^+/+^) and homozygous *Bmal1* mutant (*Bmal1* ^−/−^) mice. Surprisingly, a persistent, yet highly variable, rhythmicity emerged in *Bmal1* ^−/−^ SCN explants (Figure 1 and Video S1), whereas all peripheral tissues failed to generate PER2::LUC circadian rhythms (Figure 1A).

WT and *Bmal1* ^−/−^ SCN explants show persistent PER2::LUC bioluminescence rhythms for more than 35 d in culture (Figure 1B and 1C; also see Datasets S1 and S2). Fast Fourier transform (FFT) spectrograms of the records show a consistent frequency (about one cycle per day) for the WT SCN; however, more variable frequency components are observed in the *Bmal1* ^−/−^ SCN (Figure 1B and 1C, middle). Double-plotted raster plots illustrate the stable PER2::LUC rhythmicity in the WT SCN and the unstable rhythms in *Bmal1* ^−/−^ SCN (Figure 1B and 1C, right). FFT analysis shows that the dominant periodicity in WT SCN is tightly clustered around 24 h, while periodicity is much more variable, ranging from about 14 to 30 h, in *Bmal1* ^−/−^ SCN (Figure 1D). Because of the highly variable periods and instability of the rhythmicity from *Bmal1* ^−/−^ SCN, we refer to these fluctuations as *stochastic*. All tissues from WT littermates, including the SCN, pituitary, liver, lung, and cornea, displayed normal circadian rhythms similar to those observed in previous studies (Figure 1A) [4],[28].

Due to the stochastic nature of the rhythms in the *Bmal1* ^−/−^ SCN, period estimates based on averages of long time series do not adequately describe the cycle-to-cycle variability. Hence, we measured individual peak-to-peak intervals of the PER2::LUC expression patterns from each SCN explant (Figure 1E, left). The mean inter-peak intervals observed in the WT SCN explants were circadian at about 24 h (24.3±1.03 SD h). These values were similar to the periods estimated by Levenberg-Marquardt (LM) curve fitting of the entire time series using the LumiCycle Analysis Program (WT = 24.04±0.09 SD h, *n* = 23). By contrast, the *Bmal1* ^−/−^ SCN showed a significantly shorter average inter-peak interval length of 17.8 h with a much greater variance (SD = 5.54 h, *n* = 19) than WT SCN explants.

Using the inter-peak interval data, we determined the Serial Correlation Coefficient of successive intervals, *r_s_* (Figure 1E, right). A negative serial correlation reflects the likelihood that a long cycle will be followed by a short cycle, or vice versa, which is a characteristic feature of a functional pacemaker-driven system [39]. The average serial correlation coefficients for WT SCN explants were negative (mean *r_s_* = −0.17±0.0606 SEM, *t*-test, *p*<0.01) as would be expected from a circadian pacemaker-driven process (Figure 1E, right). However, the coefficient for the *Bmal1* ^−/−^ SCN was slightly positive and marginally significant (mean *r_s_* = 0.07±0.0336 SEM, *t*-test, *p*<0.05). This weak serial correlation coefficient differs from the pacemaker-driven processes seen in WT SCN explants and is consistent with either an oscillator with a highly labile period or a “random walk” process [40].

### Isolated *Bmal1* ^−/−^ SCN Neurons Fail to Generate Circadian Rhythms

**Figure 2:**
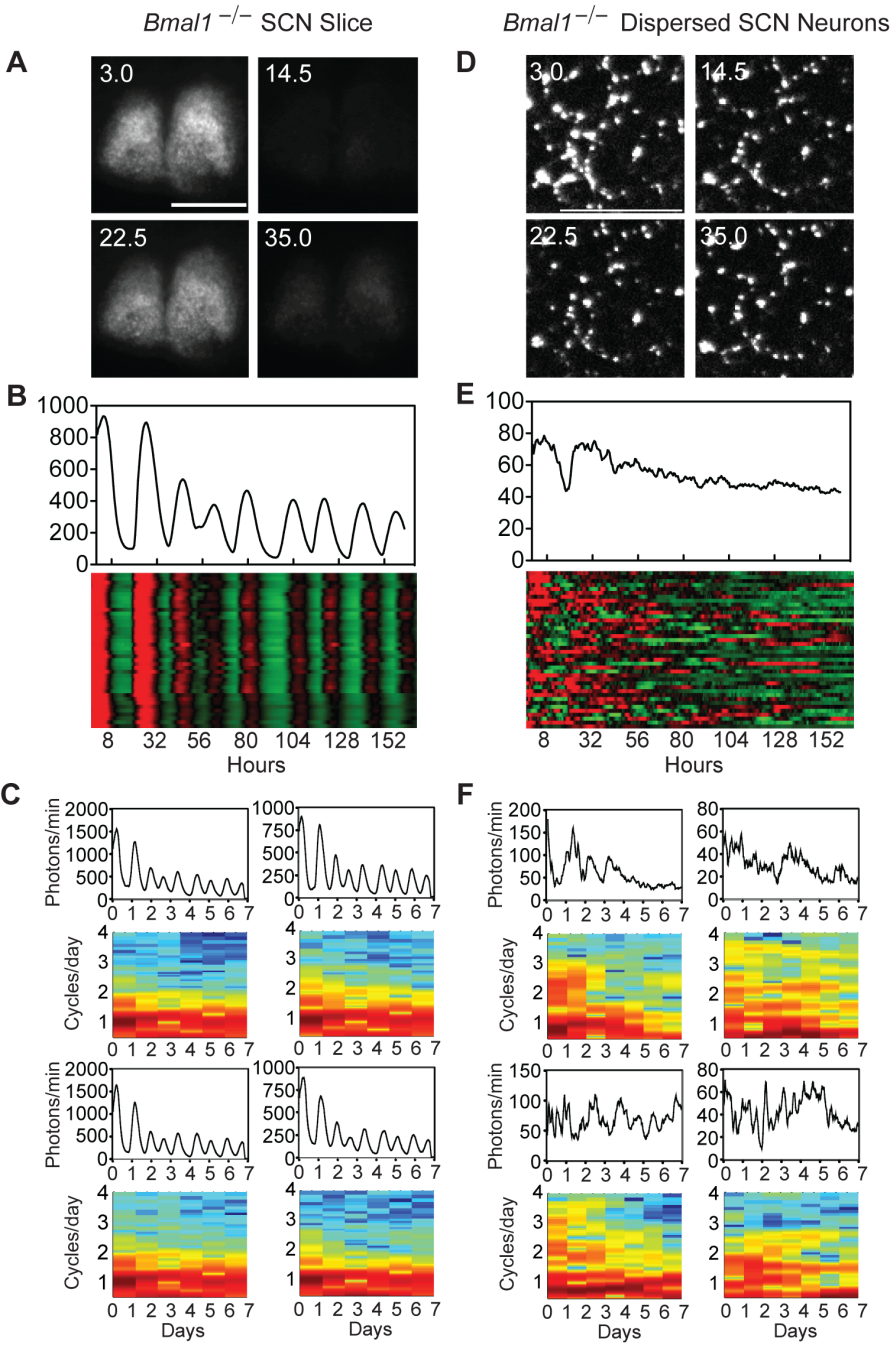
Stochastic rhythmicity in *Bmal1* ^−/−^ SCN is not cell autonomous. (A) Bioluminescence images of a *Bmal1* ^−/−^ SCN explant culture at peak and trough phases. Numbers indicate hours after start of imaging. Scale bar = 500 µm. Imaging experiments were initiated after 2–3 wk of culture. (B) Average bioluminescence and heatmap plots of bioluminescence intensity of individual *Bmal1* ^−/−^ neurons in an intact organotypic SCN slice. Forty cells are presented, with each horizontal line representing a single cell. These cells show tightly synchronized stochastic rhythms that are comparable to rhythms seen with PMT luminometry. (C) PER2::LUC rhythms (top) and corresponding FFT Spectrograms (bottom) for first 4 cells shown in (B) (i.e., coupled in *Bmal1* ^−/−^ SCN explant). (D) Bioluminescence images of dissociated individual *Bmal1* ^−/−^ SCN neurons showing nonrhythmic bioluminescence patterns. Numbers and scale bar are as in (A). (E) Average bioluminescence and heatmap plots of bioluminescence intensity of 40 individual *Bmal1* ^−/−^ neurons in dispersed culture imaged in (D), showing a lack of co-ordinated rhythmicity. (F) PER2::LUC rhythms (top) and corresponding FFT spectrograms (bottom) for first 4 cells shown in (E) (i.e., dispersed *Bmal1* ^−/−^ SCN cells).

To determine whether the stochastic PER2::LUC rhythms from *Bmal1* ^−/−^ SCN explants are cell autonomous, we studied PER2::LUC bioluminescence at the single-cell level (Figure 2). We first imaged the overt bioluminescence expression patterns from *Bmal1* ^−/−^ SCN explants (Figure 2A, Video S1, Datasets S3 and S4) and analyzed bioluminescence from individual neurons (Figure 2B and 2C). The SCN cells in an intact organotypic slice were tightly synchronized and exhibited stochastic rhythms comparable to those seen in the *Bmal1* ^−/−^ SCN explants using luminometry. Separately, we cultured dissociated SCN neurons and imaged bioluminescence from individual cells. In contrast to the cells in an intact organotypic slice, dissociated *Bmal1* ^−/−^ neurons did not express detectable circadian rhythms (Figure 2D, 2E, 2F, Video S2, Datasets S5 and S6). A total of 243 out of 243 *Bmal1* ^−/−^ SCN cells were equal to or below a threshold FFT amplitude for rhythmicity independently derived from WT SCN cells. Although *Bmal1* ^−/−^ SCN cells did not generate obvious circadian oscillations, they did express fluctuating levels of PER2::LUC—likely a reflection of *Per2* transcriptional and post-transcriptional noise (Figure 2F). The lack of rhythmicity in dissociated SCN cells indicates that the stochastic rhythmicity observed in *Bmal1* ^−/−^ SCN explants is not a cell-autonomous property and likely arises from intercellular network interactions (to be addressed later). Thus, the integrity of the cellular anatomy of the SCN is an important factor for stochastic rhythm generation in *Bmal1* ^−/−^ SCN explants.

### Stochastic Model of the Cell-Autonomous Circadian Oscillator

**Figure 3:**
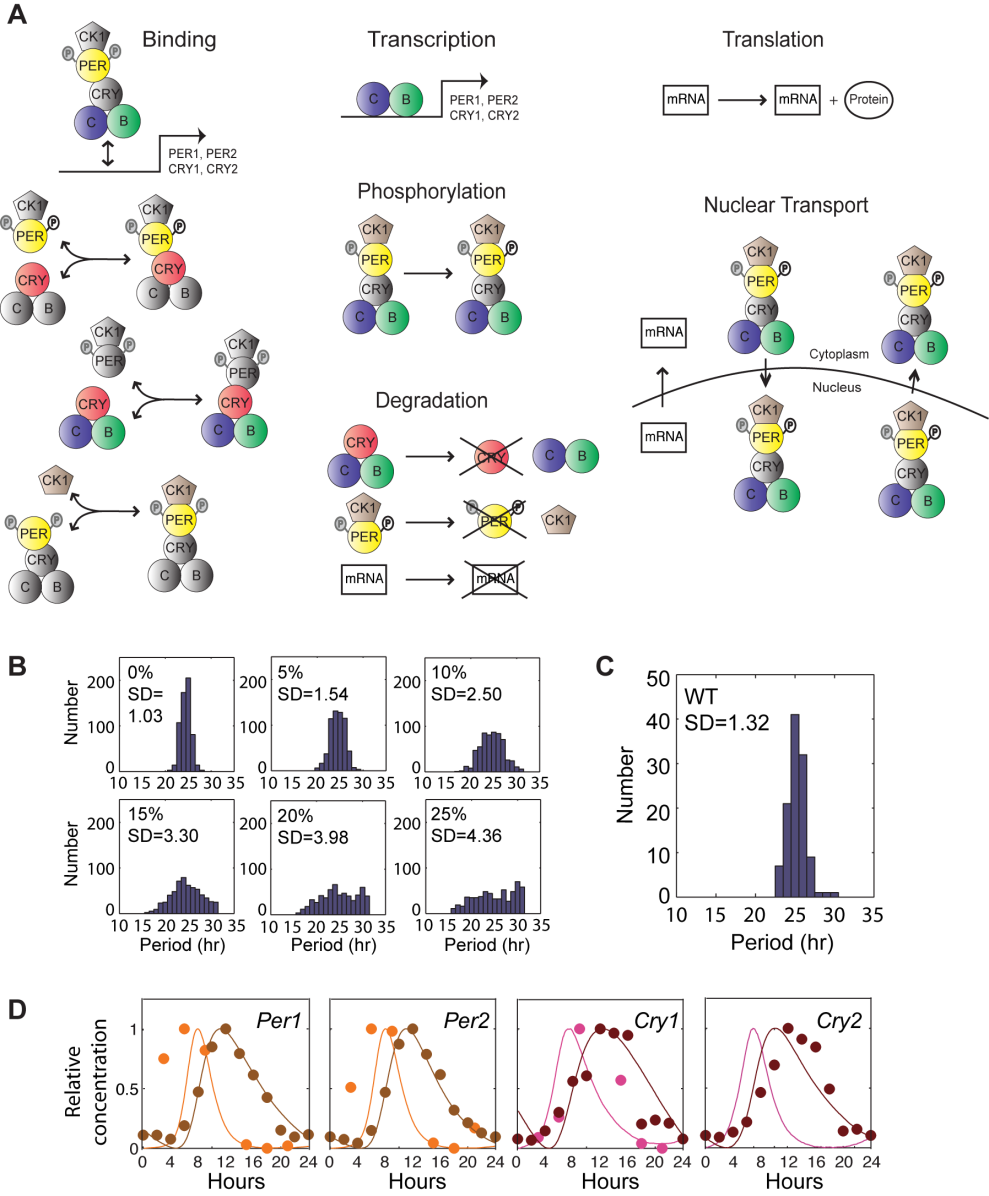
Illustration of the elements of the stochastic model of the cellular circadian clock and heterogeneity in period length. (A) The biochemical processes modeled in an SCN cell. The specific equations and rates are included in Figure S7 (see also Protocol S1). Gray components represent molecules that could be bound but have no effect on the indicated process. This model is based on the Forger-Peskin model [14] but contains three major improvements: (1) it allows for binding and unbinding of CLOCK:BMAL to *Per1*, *Per2*, *Cry1*, and *Cry2* genes; (2) it allows interaction of CLOCK:BMAL with CRY1 or CRY2; and (3) it uses updated rates of degradation of mRNAs and proteins measured empirically by Siepka et al. [41]. (B) Histograms of average period length of 250 simulated single cells when the variation in biochemical parameters is within 0%, 5%, 10%, 15%, 20%, or 25% of the mean values of the rate constants. (C) Histogram of single-cell periods experimentally measured from dissociated WT SCN cells. The mean and standard deviation values from this experiment were used to select the best match of mean and standard deviation for the simulated cells; the simulated values were comparable to experimental values at 5% variation in rate constants. (D) Representative traces of relative concentration of *Cry1/2* and *Per1/2* mRNA and protein. Circles indicate experimental data from Lee et al. [11], and lines are stochastic simulations of a population of 100 cells using the model summarized in Figure 5A. Orange and pink traces represent mRNA levels (mRNA values for *Cry2* were not reported in the original data). Brown and red traces represent protein levels.

To explore possible origins of the stochastic rhythmicity in the *Bmal1* ^−/−^ SCN, we used mathematical modeling of circadian oscillators to examine the role of intercellular coupling and molecular noise. The key clock components in our mathematical model and their possible interactions within a single SCN cell are depicted in Figure 3A. This model is similar to the Forger-Peskin stochastic model [34] but with three refinements. In the current simulation, we (1) explicitly modeled the binding of CLOCK:BMAL to regulatory regions of the *Per1*, *Per2*, *Cry1*, and *Cry2* genes; (2) explicitly modeled the interaction of BMAL with CRY1 or CRY2 and its subsequent effects on transcriptional regulation; and (3) updated the rates of degradation of mRNAs and proteins using measurements from recent experiments [41]. An explanation of these changes, as well as a list of rate constants, can be found in Figure S7 (also see Protocols S1 and S2).

We accounted for the heterogeneity of circadian period of SCN neurons by choosing all rate constants from Gaussian distributions. When no Gaussian variation is introduced to the rate constants (Figure 3B, “0%”), the standard deviation for period of the population of oscillators is 1.03 h, which reflects variability in period due to the stochastic model (i.e., no rate constant variability). As the standard deviation of the rate constants is increased, the standard deviation of the circadian period from a population of oscillators increases further (Figure 3B). When we chose the standard deviation of these Gaussians to be within 5% of their mean values, the resulting period of the model simulations had a standard deviation (SD = 1.54 h) similar to the experimental data obtained from dispersed WT SCN neurons, which have a standard deviation of 1.32 h for circadian period (Figure 3C). When these individual neurons were coupled in a network of cells (see below), the total mRNA and protein concentrations followed time courses similar to those found experimentally (Figure 3D).

**Figure 4:**
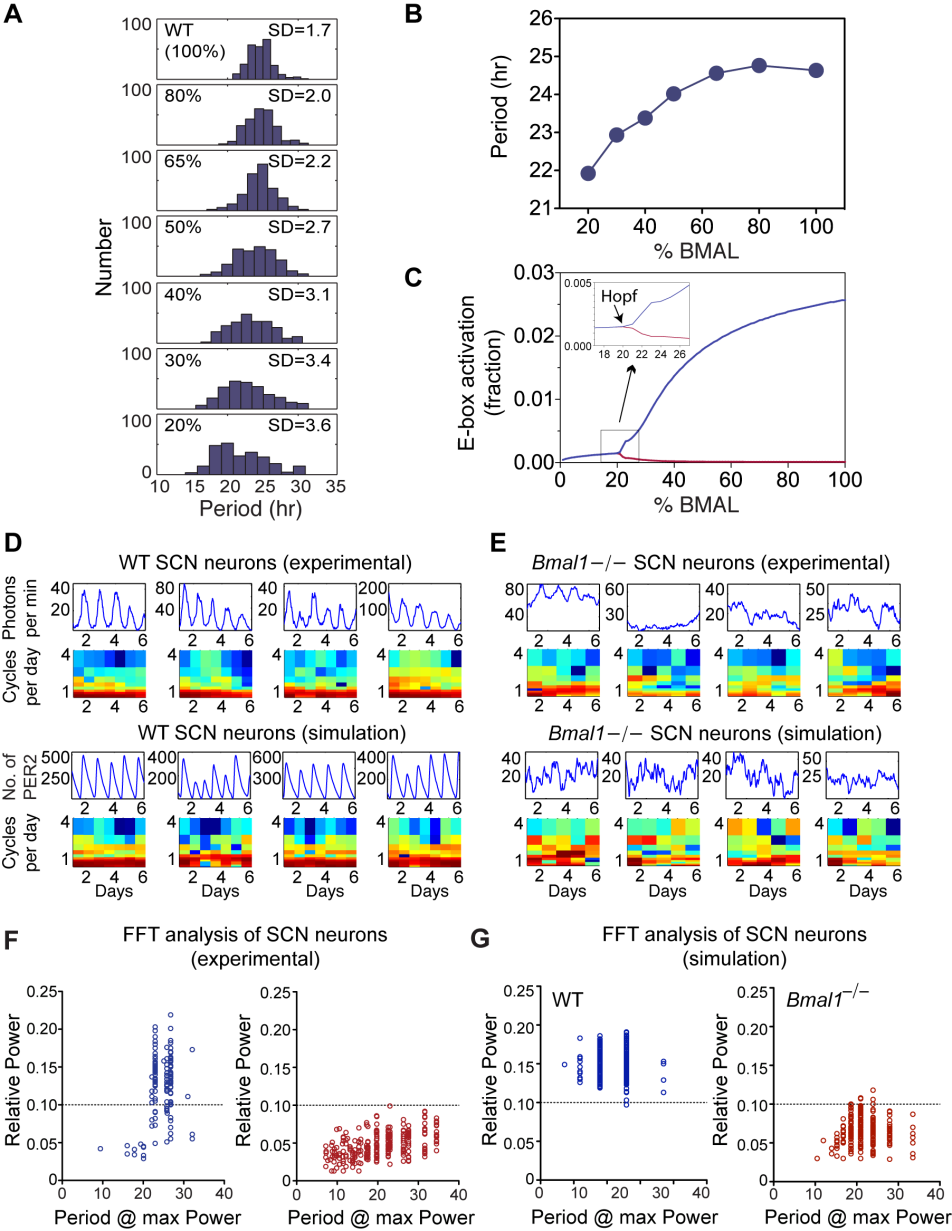
Effects of BMAL activator level on circadian oscillations in a stochastic model of isolated cellular oscillators. (A) Histograms of simulated isolated cell mean period lengths at various percentages of total WT BMAL. These results show that as the percentage of total BMAL decreases, the mean period length decreases, along with an increase in the variance of the period. (B) The figure shows that as we go below certain percentage of total BMAL, rhythms in a population of uncoupled single cells disappear. This figure is an alternative way to observe bifurcations by plotting the period from a population of single un-coupled cells as a function of total BMAL. Below ∼20% of total BMAL, rhythms disappear in single cells, indicating a Hopf bifurcation at this point. (C) The bifurcation diagram of a single oscillator as a function of total BMAL using a deterministic model. The *y*-axis plots the value G, which mathematically represents the fraction of time an E-box is activated. This was chosen since this variable affects basically all parts of the model (in particular PER1, PER2 CRY1, CRY2, all their relevant complexes, and the coupling factor). In theory, any possible variable could be used for the bifurcation diagram and the same behavior (i.e., a Hopf bifurcation) would be observed. Plotted on this diagram are the minimum and maximum (red and blue, respectively) values from the oscillation of G at a particular value of total BMAL—100% BMAL corresponds to WT BMAL. When these values are equal, the system is at rest and no oscillations are present; however, as these values begin to diverge, oscillations are observed. At approximately 22% of total BMAL, we begin to observe oscillations, indicating that a Hopf bifurcation exists at this point (see inset). Therefore, single cells show no sustained rhythmic behavior below ∼22% of total BMAL. (D) Representative traces of PER2::LUC bioluminescence measured experimentally in WT isolated SCN neurons and their respective FFT spectrograms are shown in the top row. Representative traces of WT simulated isolated cells and their respective FFT spectrograms are shown in the bottom row. (E) Representative traces and their respective FFT spectrograms of *Bmal1* ^−/−^ experimental (first two rows) and simulated isolated cells (bottom rows). These results show a loss of circadian rhythms in single *Bmal1* ^−/−^ SCN cells in both experimental and simulated isolated cells. (F) FFT spectral analysis for PER2::LUC rhythms recorded from dissociated SCN neurons. A cell was considered to show significant circadian periodicity when spectral analysis indicated a peak in the circadian range (20–36 h) large enough such that a 0.14 cycles/day window centered on the peak accounted for at least 10% of the total variance in the record (FFT power spectrum, Blackman-Harris windowing, peak amplitude ≥0.1) as described previously [40]. All (243 of 243) *Bmal1* ^−/−^ cells were equal to or below a 0.1 cutoff value for circadian rhythmicity (indicated by the dotted line), whereas approximately 80% of WT neurons were above this cutoff value and displayed circadian rhythmicity. (G) FFT spectral analysis on simulated PER2::LUC rhythms from isolated SCN neurons. A cell was considered to show significant circadian periodicity using the same criterion as in (F). All but two (248 of 250) WT cells were above the 0.1 cutoff value (indicated by the dotted line) for circadian rhythmicity. Only 5 of 250 simulated *Bmal1* ^−/−^ neurons were equal to or above this cutoff value.

This stochastic model was then used to simulate *Bmal1* ^−/−^ SCN isolated cells and the *Bmal1* ^−/−^ coupled SCN network, respectively. As a first step, we examined the effects of reducing the total BMAL activator concentration on the generation of rhythms in simulations of isolated cells. As the BMAL activator abundance was gradually decreased, the distribution of periods widened and the mean period shortened until a complete loss of rhythms was observed below 20% BMAL activator abundance in a population of uncoupled cells (Figure 4A and 4B). This behavior is consistent with bifurcation diagrams of single cell simulations (Figure 4C) that show the existence of a Hopf bifurcation at approximately 22% of total BMAL. Above this percentage, oscillations can be shown to emerge in our simulations. At 0% total BMAL activator abundance no element in the model could initiate transcription; thus, some level of residual BMAL activator must be present in the model in order for clock gene expression to occur.

In *Bmal1* null mutants, other functional E-box activator proteins may be present. The most likely candidate for “residual BMAL” activity in *Bmal1* mutants is BMAL2 (also known as MOP9; RefSeq: *Arntl2* NM_172309), a paralog of BMAL1, which is regionally co-expressed in the SCN and forms transcriptionally active complexes with CLOCK [7]. BMAL1 and BMAL2 show similar sensitivity to CRY1-mediated transcriptional repression (Figure S1; for methods, see Text S1), which suggests that the core negative feedback loop of the circadian oscillator could remain functional in the presence of BMAL2. Using quantitative PCR methods that normalize the amplification of *Bmal1* and *Bmal2* RNA, we estimate that *Bmal2* mRNA levels are approximately 10% of the level of *Bmal1* and that *Bmal2* expression levels are unaffected by *Bmal1* loss-of-function (i.e., no compensation of *Bmal2* in *Bmal1* ^−/−^ mutants) (Figure S2; for methods, see Text S1). Indeed recent work has shown that *Bmal2* can substitute for *Bmal1* if ectopically driven at high levels in *Bmal1* ^−/−^ mice [42]. Since BMAL1 and BMAL2 appear to be functionally equivalent, we use the term “BMAL” in our simulations to represent the sum total of BMAL1 and BMAL2.

On the basis of the expression analysis, we chose the 10% BMAL activator concentration to simulate the *Bmal1* ^−/−^ SCN neuron because *Bmal2* represents approximately 10% of the wild-type level of *Bmal1*; and in *Bmal1* ^−/−^ cells, the remaining *Bmal2* would contribute about 10% of the total BMAL1 and BMAL2 activity. Our simulated and experimental isolated cell rhythms show remarkable agreement at the cell autonomous level. In Figure 4D, we show representative experimental records from dissociated WT SCN cells (first row) along with their associated spectrograms (second row). Similarly, the third and fourth rows in parallel display the computational simulations for the isolated WT SCN neurons with their associated spectrograms. Comparable traces are shown for isolated *Bmal1* ^−/−^ cells in Figure 4E. Thus, the 10% BMAL activator simulations faithfully capture the stochastic behavior of PER2::LUC expression seen in dissociated *Bmal1* ^−/−^ SCN neurons.

### Effects of Intercellular Coupling on Stochastic versus Deterministic Models of the Oscillator

**Figure 5:**
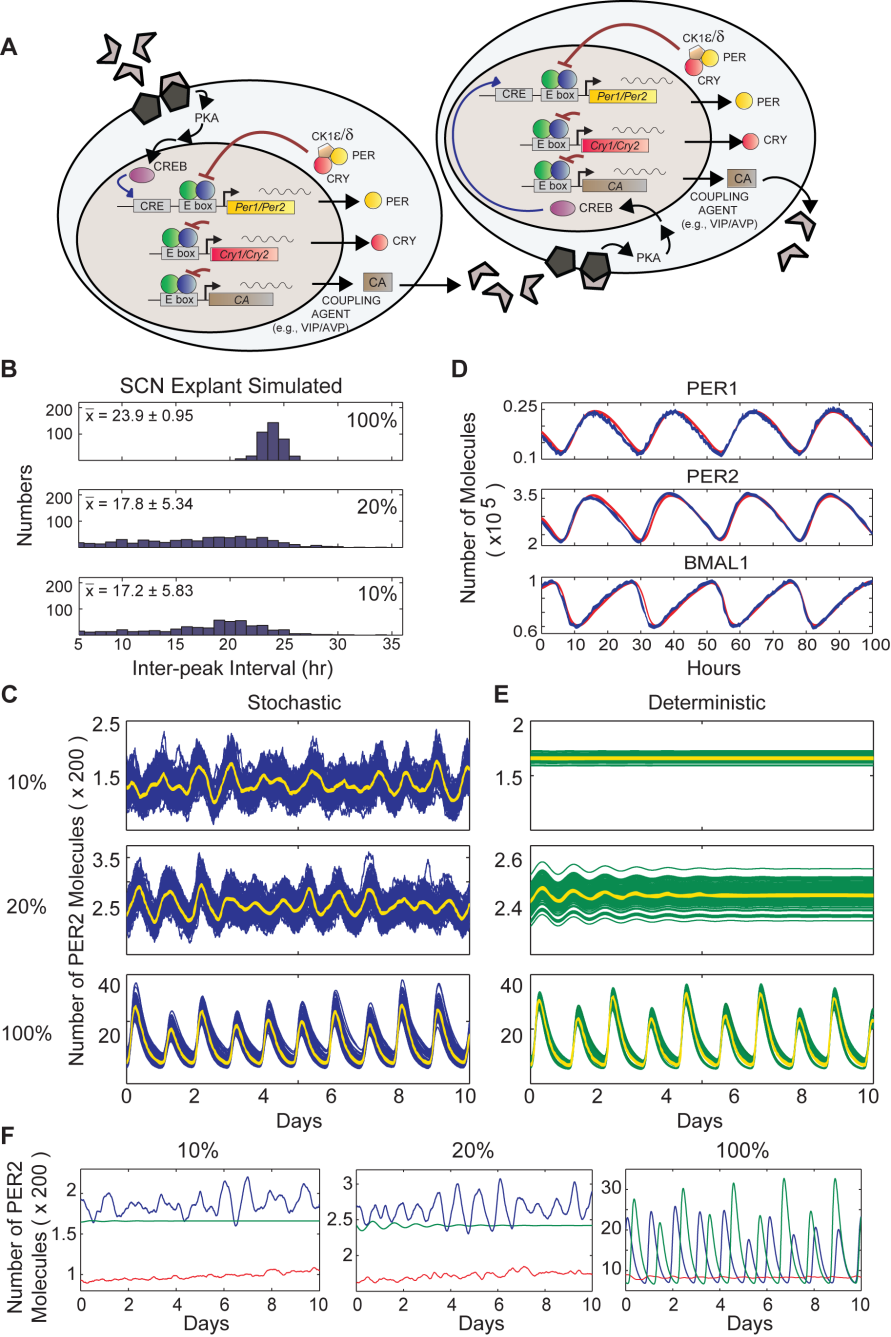
Modeling of intercellular coupling mechanisms in a population of simulated SCN cells. (A) Computational simulations applying coupling mechanisms to a population of 100 cells. The CLOCK:BMAL complex activates production of coupling agents (CA; e.g., VIP). CAs are secreted and act on cell-surface receptors on other SCN neurons, triggering cell-signaling pathways. The final product of the receptor pathways, CREB, binds to a CRE element upstream of PER. In the proposed mechanism, CREB forms a dimer and binds to a CRE element, which leads to activation of PER. CRY can repress CREB-activated PER production. (B) Histograms of average inter-peak intervals in a simulated population of coupled cells with 100%, 20%, and 10% of total WT BMAL. These results show that as the amount of BMAL is decreased, the average period decreases and the variance in the period length increases. Calculations were done as in Figure 1E. (C) The output of PER2 protein from stochastic simulations with 10%, 20%, and WT (100%) amounts of BMAL. The population averages from each simulation are plotted in yellow. (D) The proposed model was simulated for a single cell using both stochastic and deterministic approaches. In the limit of a large number of molecules, the results of the stochastic simulations (blue) agreed with the deterministic simulations (red) when the same parameter values were used. Shown in this figure are concentrations of total PER1, total PER2, and free BMAL1 proteins. Results would be similar for any other protein complex or mRNA in the proposed model. (E) The output of PER2 protein from deterministic simulations of a coupled population of oscillators with 10%, 20%, and WT (100%) amounts of BMAL. The population averages from each simulation are plotted in yellow. Contrary to the stochastic simulation (as shown above in 5C), the deterministic simulation could not sustain rhythmicity by coupling alone. (F) The average PER2 out from a simulated population of 100 cells. The left panel is the mean population rhythm of noisy coupled cells (stochastic; blue), coupled deterministic cells (green), and noisy uncoupled cells (red) at 10% of total WT BMAL. The middle and right panels are the same as the left panel, except that the cells are at 20% and 100% of total WT BMAL, respectively. These results indicate that neither noise alone nor coupling alone is sufficient to produce rhythms in a population of BMAL1-deficient cells. In addition, it is evident that coupling elevates the average number of PER molecules.

Although there are many mathematical models of the SCN that incorporate coupling of a population of circadian oscillators [16],[19]–[21], very few models have studied coupling in a population of oscillators in which the individual oscillators are stochastic in nature [26],[27]. The results in this paper represent the most detailed stochastic simulation of coupled SCN oscillators to date. To model a population of stochastic oscillators in the SCN, we utilized a group of 100 “cell autonomous” stochastic oscillators as described in the previous section and coupled the population of oscillators (see Figures 5, S3, and S4). For the coupling pathway, we used a model of the VIP signaling pathway in the SCN, whereby a rhythmic coupling agent (CA) is released by one SCN cell and affects neighboring SCN cells by activating an adenosine 3′,5′-monophosphate (cAMP) signaling pathway, which ultimately activates cAMP response element binding (CREB) protein and cAMP response elements (CRE) on the *Per1* and *Per2* promoters to induce PER1 and PER2 proteins. In our proposed model, the intercellular coupling is all-to-all. We assumed that CRE activation of *Per1* and *Per2* could occur only when repressors were not bound as suggested in previous experiments [43]. Thus, this is the equivalent of allowing CRY to repress CREB-mediated PER production.

When coupling is included in a population of WT single-cell models, this coupled cellular network was able to produce coherent ∼24-h rhythms (Figure 5B, top, and 5C, bottom) as seen in WT SCN explants and in other coupled oscillator models of the SCN [16],[19]–[21]. Remarkably, as seen in experiments, rhythmicity also emerged when a population of nonrhythmic *Bmal1* ^−/−^ SCN simulated neurons was coupled together (Figure 5C, top and middle). To compare these rhythms to those found experimentally, we calculated the peak-to-peak intervals of the rhythms from the SCN network with 100%, 20%, and 10% BMAL activator concentrations (Figure 5B). The distribution of these inter-peak intervals is similar to experimental measurements from WT and *Bmal1* ^−/−^ explant rhythms (Figure 5B; compare with Figure 1E). The observed decrease in period in the simulated SCN explants with lower activator concentrations is similar to that observed in simulated single cells, implying that the short period of the *Bmal1* ^−/−^ explant may be attributable to a shortened intracellular, single-cell periodicity. However, given the general absence of rhythms in individual uncoupled *Bmal1* ^−/−^ cells, it is interesting that coupling of these intrinsically nonrhythmic cells (10% BMAL) can generate stochastic rhythms.

While only three sets of simulations are shown in Figure 5B and 5C, we systematically varied the coupling strength and BMAL concentration to determine the amplitude of rhythms and synchrony of the network in Figure S3. This shows that the stochastic rhythmicity can be seen over a wide range of values for the coupling strength. In addition, we note that the relative amplitudes of the *Bmal1* ^−/−^ simulations are reduced when compared with those found experimentally. Figure S3 shows that the amplitude of these rhythms depends greatly on the coupling strength and mechanism. In particular as the coupling becomes nonlinear, the amplitude increases. Including other nonlinear aspects of the coupling (e.g., electrical activity) could also increase the amplitude. In addition, there are likely to be other transcriptional inputs to the *Per* and *Cry* genes that could affect amplitude and that were not included in the model.

Since the emergent rhythms in the *Bmal1* ^−/−^ SCN explants were stochastic, we hypothesized that the molecular noise, inherent in all biochemical reactions and included in our current model through our use of the Gillespie stochastic simulation algorithm, could play an important role. To test this hypothesis, we developed a deterministic version of our proposed model to understand the implications of the noise. The deterministic model represents a version of our proposed model without any noise. Each cell in the deterministic model had its parameters chosen from a distribution as was done in the stochastic model. Once the parameter was chosen it remained fixed for the rest of the simulation. For example, the bifurcation analysis in Figure 4C used the deterministic model with its mean parameters. The stochastic and deterministic models produced equivalent results when the number of each molecule species (Figure 5D) in the stochastic model was increased. This equivalence serves as a check on the accuracy of both the stochastic and deterministic simulation approaches. The output of 100 simulated cells is shown using both the stochastic and deterministic models in Figure 5C and 5E. The total BMAL concentration was varied from 10% to 20% and finally 100% of the activator level in WT SCN, and the ensemble averages from each simulation are plotted in yellow. Strikingly, when a population of cells using the deterministic model was coupled, rhythmicity could not be recovered at 10% BMAL activator levels and only damped rhythms could be seen at 20% BMAL activator levels (Figure 5E).

The discrepancy between the stochastic and deterministic models clearly indicates that noise is a necessary but not sufficient condition for the rhythmicity observed in the *Bmal1* ^−/−^ SCN explants. We reach this conclusion because the deterministic model fails to show any sustained rhythmicity at the 10% or 20% level of BMAL activator concentration. Our analysis indicates that noise alone could not restore the rhythmicity in individual cells, nor is the coupling mechanism alone sufficient to induce oscillations in a population of cells without noise. Thus, what is necessary to induce rhythms in a population is a joint requirement for both molecular noise and intercellular coupling.

### Uncoupling Abolishes the Stochastic Rhythms in *Bmal1* ^−/−^ SCN Slices

**Figure 6:**
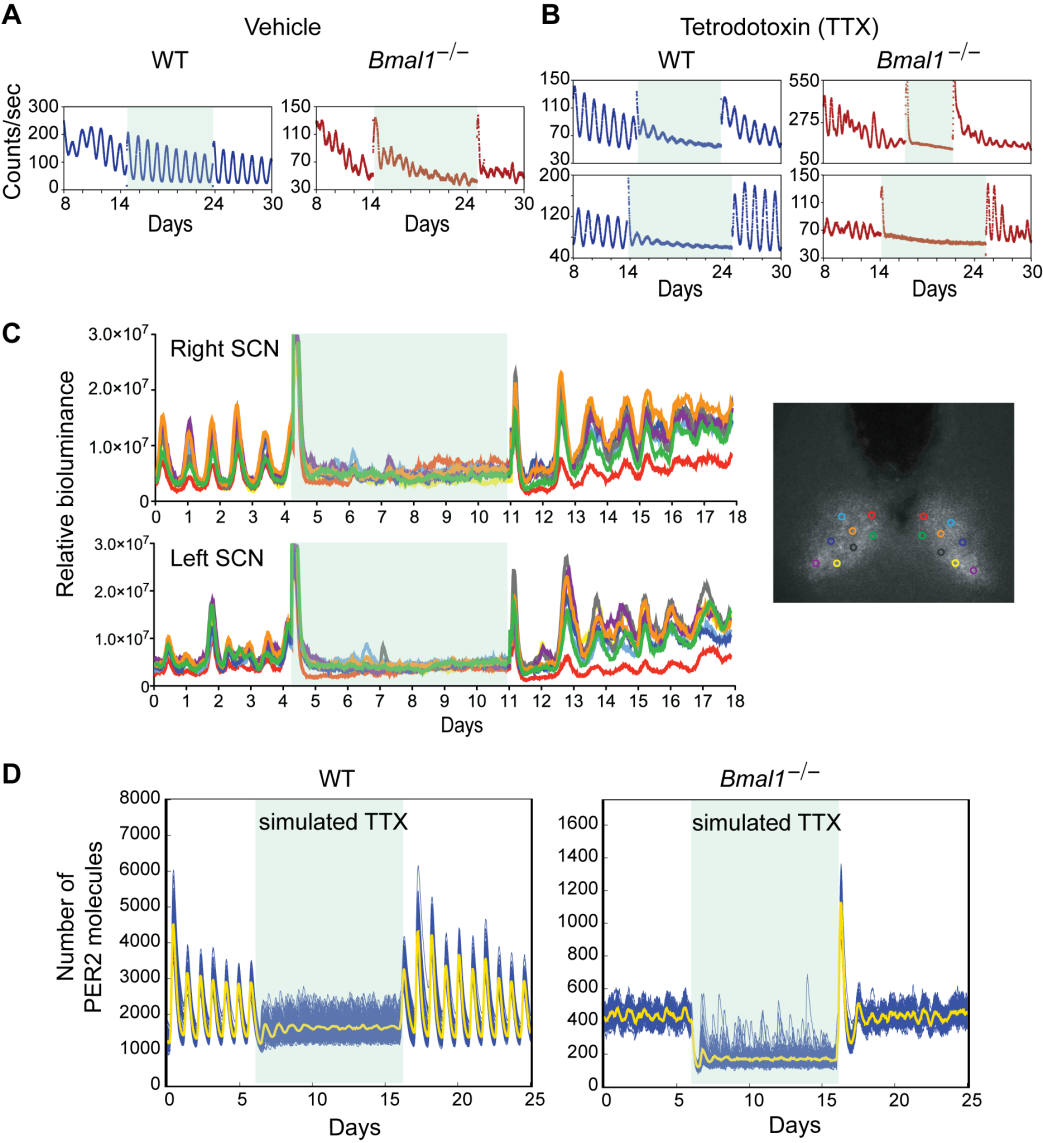
Uncoupling SCN cells abolishes stochastic rhythms from *Bmal1* ^−/−^ SCN explants. (A and B) Representative records of PER2::LUC rhythms of the SCN explants from WT (blue) and *Bmal1* ^−/−^ (red) mice. Data are shown following a medium change (day 8); shaded area indicates when SCN explants were changed to fresh medium containing vehicle solution (A) or tetrodotoxin (B). (C) Single-cell rhythmicity before, during, and after TTX treatment from cells within an intact *Bmal1* ^−/−^ SCN slice. On the right is a PER2::LUC bioluminescence image of the *Bmal1* ^−/−^ SCN explant showing color-coded locations of the analyzed cells. Uncoupling cells by TTX treatment within an intact organotypic SCN slice results in arrhythmic single-cells with average PER2::LUC level similar to that of SCN neurons in dispersed culture (see Figure 2F). Records of individual *Bmal1* ^−/−^ and WT cells are shown in Figure S5. (D) WT and *Bmal1* ^−/−^ SCN networks are simulated with normal coupling, with coupling reduced to 0% of its original value (simulating the effect of TTX), and with coupling slowly restored (with a time constant of 2 h).

The coupled stochastic model of the *Bmal1* ^−/−^ SCN predicts that both noise and coupling are required to generate stochastic rhythms. To test the role of coupling in SCN explants, we used a variety of agents previously reported to uncouple SCN neurons. We first used tetrodotoxin (TTX) treatment on *Bmal1* ^−/−^ SCN rhythms (Figure 6). TTX prevents action potentials by selectively and reversibly blocking voltage-dependent Na^+^ channels. TTX application has been shown to desynchronize neurons within an intact SCN [44], suggesting that action potentials and/or consequent neuronal transmission are required for maintaining SCN synchrony. Under continuous TTX administration, WT SCN tissue showed persistent PER2::LUC rhythms. However, the amplitude of the rhythm gradually diminished cycle by cycle (damping), which is attributable to intercellular desynchrony and a reduction in amplitude at the individual oscillator level [44]. In contrast, the *Bmal1* ^−/−^ SCN exhibited an immediate loss of stochastic rhythmicity of PER2::LUC output when treated with TTX. The PER2::LUC rhythms returned immediately upon removal of TTX in both WT and *Bmal1* ^−/−^ SCN explants (Figures 6B and S5). To determine whether the loss of stochastic rhythmicity was due to desynchrony of individual cellular rhythms, we used bioluminescence imaging to measure single-cell behavior during TTX treatment (Figures 6C and S5). Uncoupling cells by TTX treatment within an organotypic *Bmal1* ^−/−^ SCN slice caused a loss of stochastic rhythmicity at the single-cell level with PER2::LUC patterns similar to those of SCN neurons in dispersed culture. Thus, TTX treatment abolished stochastic oscillations by preventing the sustainment of rhythmicity at the single-cell level.

Using the coupled stochastic model of the SCN, we mimicked the experiments that blocked coupling via TTX and then restored the coupling by removing TTX (Figure 6D). Our simulation results reproduce the experimental data for both the WT and *Bmal1* ^−/−^ SCNs; these results demonstrate that abolition of coupling alone in the model can account for the observed patterns of PER2::LUC bioluminescence from *Bmal1* ^−/−^ SCN when blocked with TTX.

Similar findings were observed when other pharmacological agents were used to uncouple the SCN neurons (Figure S6). For example, pertussis toxin treatment, which blocks G_i/o_-mediated signal transduction, and bicuculline, which blocks GABA_A_-receptors, both abolished PER2::LUC stochastic rhythmicity—these agents have previously been shown to uncouple SCN cells [45],[46].

### Role of the cAMP and PER Induction Pathway in Coupling and Period Determination

**Figure 7:**
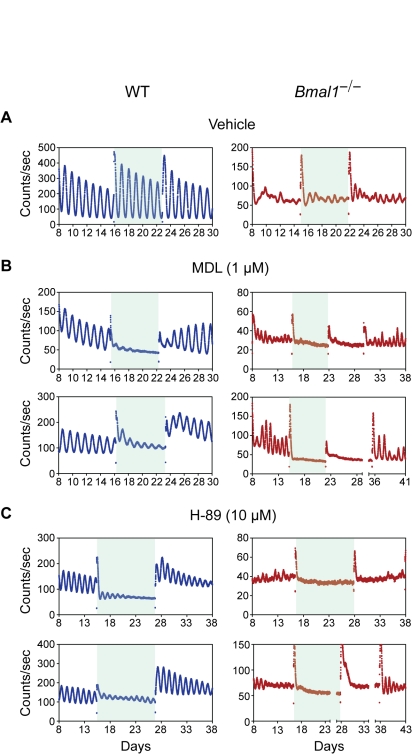
Inhibition of cAMP signaling abolishes stochastic rhythms from *Bmal1* ^−/−^ SCN explants. (A–C) Representative records of PER2::LUC rhythms of the SCN explants from WT (blue) and *Bmal1* ^−/−^ (red) mice. Data are shown following a medium change (day 8); shaded area indicates when SCN explants were chronically treated with vehicle solution (A), 1 µM MDL (B), or 10 µM H-89 (C). WT SCN explants recovered rhythms immediately following the washout of MDL (B, left) or H-89 (C, left); however, almost all *Bmal1* ^−/−^ explants required second washout to reinitiate their rhythms (B and C; right).

A prominent pathway for coupling SCN neurons involves the VIP receptor-signaling pathway that activates cAMP and CREB-mediated induction of *Per1* and *Per2* transcription [37],[38],[43],[47],[48]. To test the role of the cAMP signaling pathway in coupling in SCN explants, we used MDL-12,330A (MDL), a potent inhibitor of adenylyl cyclase, and H-89, an inhibitor of cAMP-activated protein kinase (protein kinase A; PKA) (Figure 7). Previous studies have shown that MDL reduces cAMP concentrations to basal levels in SCN cells [47], and H-89 prevents VIP- or calcium-induced circadian transcription in SCN cells [49]. Similar to the effect of TTX treatment, WT SCN explants showed damped rhythms with prolonged exposure to these inhibitors of cAMP-mediated signal transduction (Figure 7B and 7C, left), and the *Bmal1* ^−/−^ SCN explants showed an immediate loss of stochastic rhythmicity (Figure 7B and 7C, right). Thus, the cAMP pathway appears to be necessary for the generation of stochastic oscillations.

**Figure 8:**
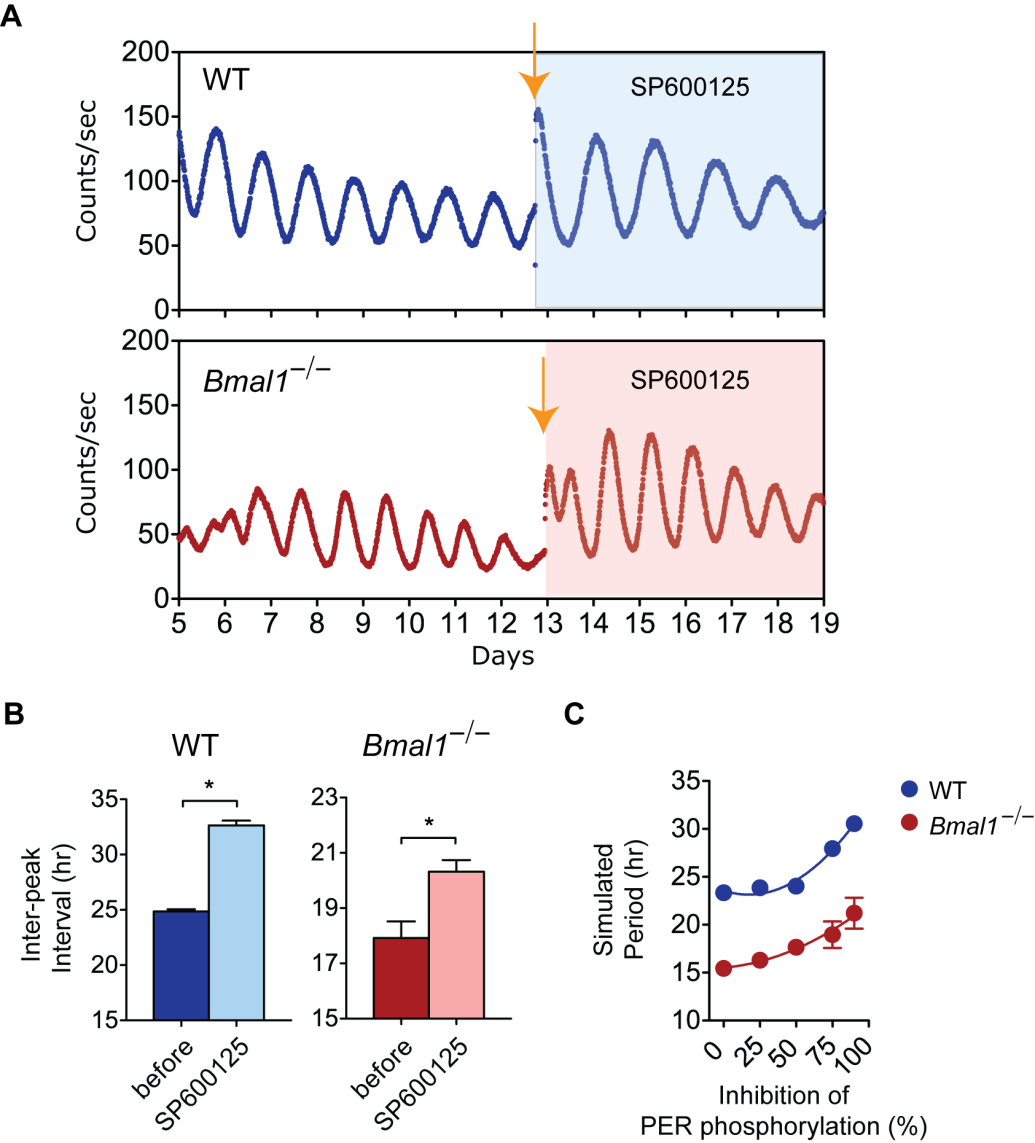
Effects of SP600125 on periodicity in WT and *Bmal1* ^−/−^ SCN explants. (A) Representative records of PER2::LUC rhythms from SCN explants of WT (top) and *Bmal1* ^−/−^ (bottom) mice. Shown are 8 d of bioluminescence record before SP600125 treatment followed by 6 d of bioluminescence record during the SP600125 (25 µM) treatment (shaded). At the time indicated by orange arrow, individual SCN explants were changed into a fresh medium with the kinase inhibitor. (B) Average inter-peak intervals of PER2::LUC rhythms (±SEM) of WT (left; before = 24.86±0.19 h, SP600125 = 32.65±0.43 h) and *Bmal1^−/−^* (right; before = 17.92±0.60 h, SP600125 = 20.32±0.42 h) SCN explants. SP600125 treatment lengthened the intervals in both WT (paired *t*-test, *p*<0.0001, *df* = 5) and *Bmal1^−/−^* (paired *t*-test, *p*<0.0005, *df* = 6) SCN explants. (C) Average period (±SEM; *n* = 6 per data point) from simulated WT (blue) and *Bmal1* ^−/−^ (red) SCN networks with varying percent inhibition of PER phosphorylation by CK1. The error bars (±SEM) cannot be seen in some of the data plots because the symbol is larger than the bar. Period values of WT and *Bmal1* ^−/−^ SCN networks were significantly different from each other and as a function of inhibition of PER phosphorylation (two-way ANOVA, *p*<0.001).

In the circadian clock mechanism, the half-life of the PER proteins contributes to the determination of circadian period, which is under the control of CK1ε/δ-mediated PER phosphorylation [50]–[52]. Modeling studies have also suggested that the *Per2* feedback loop, in particular, may have a dominant role in setting the period of circadian oscillation [53]. The VIP/cAMP signaling pathway ultimately converges on *Per* induction, and thus the periodicity of stochastic rhythms in *Bmal1*-null mutant SCN may also depend upon PER. To test a role for PER proteins in determining the period of stochastic rhythms, we examined the effects of agents that inhibit CK1ε/δ to lengthen circadian period. We treated SCN explants with a potent protein kinase inhibitor, SP600125, which has been shown to lengthen circadian period and to inhibit CK1ε kinase activity [51]. SP600125 treatment lengthened WT SCN circadian period to over 30 h (Figure 8A, top and 8B, left) as shown previously. Interestingly, SP600125 also significantly lengthened the period (average inter-peak intervals) of the stochastic rhythms from *Bmal1* ^−/−^ SCN explants (Figure 8A, bottom and 8B, right). Modeling experiments in which the phosphorylation rate of PER by CK1 was reduced also lengthened the periodicity of WT and *Bmal1*-mutant SCN simulations in a similar fashion (Figure 8C). Thus, the kinetics of the quasi-circadian periodicity that emerges from the SCN network are regulated by a PER-dependent process in which the phosphorylation rate of PER by CK1 can determine the periodicity of the emergent network oscillation.

## Discussion

The circadian pacemaker in the SCN plays a dominant role in the generation and control of circadian behavioral rhythms in mammals [54],[55]. While it is well established that the generation of circadian rhythmicity is a cell-autonomous property of SCN neurons [1] and that coupling plays an important role in enhancing the precision of circadian oscillations [56], it has not previously been demonstrated that the SCN network itself, in the absence of cell autonomous oscillatory function, can generate quasi-circadian oscillations. The experiments reported here argue strongly that a PER-dependent neural coupling mechanism in the SCN can provide a feed-forward signal to drive the circadian network and propagate quasi-circadian oscillations under conditions when cell-autonomous circadian oscillations have been abolished by the loss of *Bmal1* function. Mathematical modeling experiments comparing stochastic and deterministic models of the coupled SCN network strongly suggest that both molecular noise and intercellular coupling are essential for the generation of “emergent” oscillations from the network.

While it is clear that coupling is essential for normal SCN function [37],[38],[44] and is responsible for the robustness of SCN oscillations [28],[56], there are no precedents demonstrating that coupling per se can generate oscillations in the circadian domain. In the field of central pattern generators, rhythmic outputs can be generated by circuits that are devoid of intrinsically rhythmic pacemaker neurons [57]. In such cases, rhythmicity is an emergent property of the network. The time course of central pattern generators, however, is orders of magnitude faster than circadian oscillations. Thus, it seems likely that the mechanisms leading to oscillations in these two time domains will differ. This indeed appears to be the case since transcriptional rather than synaptic mechanisms underlie the long time constants in the circadian range.

Because the mammalian circadian clock mechanism involves gene expression, and gene expression is affected by molecular noise [58]–[60], noise is expected to influence circadian clocks. Indeed molecular noise is a key contributor to the stochastic nature of intracellular rhythmicity [61], and overcoming molecular noise has been proposed as a key principle in the design of circadian clocks [35]. However, in these previous examples, reduction rather than enhancement of noise has been the key factor. A novel finding of the present study is that molecular noise can be an integral part of the functional SCN network. Molecular noise is amplified by a sensitive coupling mechanism among SCN neurons and can “kick start” oscillations within the network. Future modeling work, using more detailed models of electrical [62],[63] and chemical [16],[21],[64] signaling in the SCN, can be used to identify the specific nonlinearities that can cause noise-induced oscillations [17]. The highly sensitive nature of the coupling mechanism allows for the amplification of not only the oscillatory signals but also the noise intrinsic in the overall network. This noise amplification can explain why the rhythms in *Bmal1* ^−/−^ SCNs are highly variable. Therefore, sensitive transcriptional regulation mechanisms that play a crucial role in promoting rhythmicity are also likely to amplify the effects of molecular noise. This finding is consistent with previous studies, indicating how certain choices of transcriptional regulatory mechanisms can greatly amplify the effects of molecular noise thereby leading to noise-induced oscillations [31]–[35].

Our simulations were able to compare mathematical models with and without noise, something that cannot be easily accomplished experimentally at this time. Molecular noise depends on the number of reactions that take place in a system. Although future experimental techniques may be able to address this issue, they do not exist for the larger mammalian genetic networks [65]. Also, stochastic bifurcation theory could be applied to understand our numerical results mathematically.

Classic work has shown that noise can induce oscillations [66]. In addition, there is a growing body of literature that demonstrates how molecular noise can enhance the behavior of cellular networks within many organisms (reviewed in [58]). Similar theoretical and experimental studies of the somite segmentation clock in vertebrate development have shown how coupling can be used to reduce the effects of noise [67],[68]. Another recent modeling paper has shown how coupling and noise can synergistically enhance calcium oscillations in two coupled cells [69]. This does not mean that noise is beneficial in all circumstances. However, we demonstrate here that the effects of molecular noise can propagate in a large network of coupled cells and may even be beneficial. This result is counterintuitive since previous work indicates that coupling diminishes the effects of noise as one increases the number of cells in the network [17]. Thus, our work establishes the importance of molecular noise in the functioning of intercellular networks. The emergence of stochastic circadian oscillations from the SCN network in the absence of cell-autonomous circadian oscillatory function highlights a previously unrecognized level of circadian organization. While we acknowledge that it is difficult to assess the role of such noise-induced oscillations in the wild-type animal in which oscillations from the cell autonomous oscillators would be expected to dominate the ensemble output from the SCN network, the resonance in the coupling network highlighted here could contribute to the robustness of the mammalian circadian system. In addition, network oscillations of the type reported here could also underlie other rhythmic phenomena such as the food-entrainable oscillator or the methamphetamine-inducible oscillator, which are thought to be independent of the circadian pacemaking system in the SCN [70]–[74]. These normally occult oscillators appear to require either restricted food availability or psychostimulant drugs to activate or reveal their presence. Perhaps feed-forward inputs from feeding signals or psychostimulants can generate quasi-circadian oscillations in behavior in a manner analogous to the stochastic SCN network oscillations reported here.

## Materials and Methods

### Animals and Behavioral Analysis

All animal care and experimental treatments were approved and performed in strict accordance with Northwestern University and University of Texas Southwestern Medical Center guidelines for animal care and use. Mice were bred from PER2::LUC (http://jaxmice.jax.org/strain/006852.html) homozygous parents [4] mated with *Bmal1* ^+/−^ mice [4],[7]. From the first generation of pups, mice carrying a copy of the *Per2::luc* (*luc*/+) and heterozygous for the mutation in *Bmal1* were then crossed to obtain *Bmal1* ^+/+^, *Bmal1* ^+/−^, and *Bmal1* ^−/−^ mutants harboring the PER2::LUC reporter.

At approximately 8–10 wk of age, mice were placed in individual running wheel cages, and activity was recorded using the ClockLab data collection system (Actimetrics, Wilmette, IL) [75]. After 2 wk in LD 12∶12, the mice were released into constant darkness (DD) for an additional 4 wk. Animals were then returned to LD 12∶12 for at least 2 wk before their tissues were harvested for bioluminescence experiments.

### SCN Explant Culture and Bioluminescence Data Analysis

Animals were euthanized by cervical dislocation between ZT 11–13. The tissues were removed immediately and put in Hank balanced salt solution (HBSS; with 10 mM HEPES, 25 units/ml penicillin, and 25 µg/ml streptomycin) on ice. Brain slices containing the SCN were sectioned at 300 µm using a vibratome followed by scalpel dissection of the SCN, resulting in a piece of tissue about 1 mm×1 mm in size. The peripheral tissues were dissected into pieces approximately 1 mm^3^ in size, with the exception of the pituitary, which was cultured as a whole. Each dissected tissue was cultured on a Millicell culture membrane (PICMORG50, Millipore) with 1.2 ml DMEM medium (Cellgro), supplemented with 10 mM HEPES (pH 7.2), 2% B27 (Invitrogen), 25 units/ml penicillin, 25 µg/ml streptomycin, and 0.1 mM luciferin (Promega) as described previously [76]. Medium changes were performed by lifting the Millicell culture membrane and placing it into a new culture dish prepared with fresh medium. For peripheral tissues, forskolin (10 µM) was administered for ∼30 min to synchronize the cells before placement into fresh medium. For uncoupling experiments, TTX (1 µM), BIC (200 µM), PTX, (5 ng/ml), MDL (1 µM), and H-89 (10 µM) were used. To test the role of PER2 in intercellular coupling and period determination, the protein kinase inhibitor, SP600125 (25 µM), was used. Drugs were added during medium change and were left undisturbed until replacement with fresh medium. All reagents were purchased from Sigma-Aldrich.

Explant cultures were maintained at 36 °C either in an incubator or in a temperature-controlled room. The bioluminescence was continuously monitored with LumiCycle photomultiplier tube (PMT) detector systems (Actimetrics, Wilmette, IL). Dark counts from the PMT were measured with luciferin-containing medium alone and subtracted from overall bioluminescence. Bioluminescence was measured immediately upon placement in culture, continuing without interruption for >7 d.

Bioluminescence analyses were performed using the LumiCycle Analysis Program (Actimetrics, Wilmette, IL). Raw data were baseline fitted. Then peak-to-peak durations (inter-peak intervals) were measured by manually identifying individual peaks. Serial correlation coefficient estimates were calculated for 7 to 10 cycle epochs using methods described previously [39].

### SCN Slice and Dispersed Cell Culture for Imaging

*Bmal1* ^−/−^ SCN slices used for imaging were dissected from 4–10-d-old pups, cut by tissue chopper (Stoelting) to a thickness of 400 µm, and cultured on Millicell-CM membrane inserts (PICMORG50). SCN slices were maintained in culture for 2–3 wk before imaging. For TTX experiments, adult SCN explants were dissected as described in the previous section.

For preparation of dispersed SCN neurons, we used 2–5-d-old *Bmal1* ^−/−^ pups (or WT controls from the same heterozygous breeding line). Cylindrical punches of unilateral SCN were made from 400 µm coronal sections using a 20-gauge needle. Punches from 2–6 mice were pooled in each preparation, and the experiment was performed twice for each genotype. Cells were dissociated using papain and cultured as previously described [1], except that medium contained 5% FBS instead of rat serum. SCN neurons were maintained in culture for 2–7 wk before imaging.

### Single-Cell Imaging

Bioluminescence imaging was performed as previously reported [3],[28],[77]. Just before imaging, medium was changed to fresh explant medium containing 1 mM luciferin. Culture dishes were sealed and placed on the stage of an inverted microscope (Olympus IX71) in a dark room. A heated lucite chamber around the microscope stage (Solent Scientific, UK) kept the cells at a constant 36°C. For experiments presented in Figure 2 and Videos S1 and S2, images were collected using an Olympus 4× XLFLUOR (NA 0.28) or UPlanSApo (NA 0.16) objective and transmitted to a CCD camera (Spectral Instruments SI800, Tucson, AZ) cooled to −92°C. For dispersed neurons, signal-to-noise ratio was improved by 2×2 binning of pixels. Images of 29.8 min exposure duration were collected at 30 min intervals for 7 d. SCN neuron viability was assessed by cell morphology and stability of average daily bioluminescence, and no differences were observed between WT and *Bmal1* ^−/−^ cells. For TTX experiments, adult SCN slice images were collected with a dual microchannel plate intensified gallium arsenide phosphide XR/MEGA-10Z CCD camera (Stanford Photonics, Inc., Palo Alto, CA) cooled to −20°C.

### Single-Cell Imaging Data Analysis

Bioluminescence was analyzed using MetaMorph (Molecular Devices) as previously described [3],[28],[77]. For TTX experiments, CellCycle single-cell analysis software (Actimetrics, Wilmette, IL) was used. Cells that were clearly discriminable from adjacent cells and that remained bioluminescent for the entire experiment were selected for analysis. Bioluminescence time series were first imported into LumiCycle Analysis v. 2.31 (Actimetrics). A linear baseline was subtracted from raw data (polynomial order = 1). Due to high initial transients of luminescence in some cases, the first 12 h of data were excluded from analysis in all cells. Maximum spectral power value was determined using FFT-Relative Power, which was defined as relative spectral power density at the peak within the range of 0–36 h, i.e., proportion of total variance within a 0.14 cycles/day window centered at highest point within the range of 0–36 h. A cell was considered to show significant circadian periodicity when the spectral analysis indicated a peak in the circadian range (20–36 h) large enough such that a 0.14 cycles/day window centered on the peak accounted for at least 10% of the total variance in the record (FFT power spectrum, Blackman-Harris windowing, peak amplitude ≥0.1) as described previously [78]. This is the same criterion used in our previous study of mutant SCN neurons [28], chosen so as to include all clearly rhythmic WT cells. Comparisons significant by ANOVA (*p*<0.05) were further explored by pairwise *t*-tests. Period was defined as the period of the best-fit sine wave as estimated by a Levenberg-Marquardt algorithm.

### Mathematical Simulation

Simulations used a revised version of the Forger-Peskin stochastic model of the mammalian circadian clock [14],[34]. We explicitly modeled the binding and unbinding of CRY1 or CRY2 (and any other bound proteins) with CLOCK:BMAL (which has the same biochemical properties as the CLOCK:BMAL1 or CLOCK:BMAL2 complexes, which were treated equivalently). Based on experimental evidence [28],[79], we assumed that the concentration of these protein complexes was constant (1,600 molecules per cell for the WT case). The original Forger-Peskin model [14] contained a binding event of the CRY proteins to activator on the promoter. This was replaced by a binding event of CLOCK:BMAL to the promoter. Consistent with fitting of experimental data [41] we assumed that there was just one active E-box on the promoters of *Per1*, *Per2*, *Cry1*, and *Cry2*. Reaction rates can be found in Figure S7.

We also modeled a CRE element where a factor (e.g., CREB) can bind and activate transcription of *Per1* and *Per2* or a secreted CA. The “coupling” factor is equivalent to AVP or VIP and mediates signaling between SCN neurons (see Figure 5). Since medium changes can stimulate transcription, presumably through this CRE element, we assumed that there was a constant low level of this factor in the network (one molecule per cell). Removing this constitutive activation had little effect on our simulations (unpublished data).

Due to the revised transcription mechanism, new rates of transcription were chosen to give qualitative agreement of the uncoupled model with protein concentration levels determined experimentally by Lee et al. (2001) [11]. Other parameters were fit to published experimental data on the mRNA and protein time courses used in previous models [14],[34]. Degradation rates for *Per1/2* and *Cry1/2* mRNAs and CRY1 and PER2 proteins were obtained directly from published experimental data [41]. This yielded a model with a slightly short period (22.9 h) that was scaled to give a 24-h period.

Simulations were performed using the Gillespie Algorithm as in Forger and Peskin (2005) [34] or MATLAB's ode15s with a maximum time step of 0.05 h. In the limit of large numbers of molecules, these simulations matched, as did a version of the model coded in Mathematica. Simulations typically lasted 500 h; the first 50–100 h were discarded to remove the effects of initial transients. Using MATLAB, spectrograms of the experimental and simulated single cell and slice data were produced. Specifically, the “spectrogram” command was used, with window size 128, overlap set to 100, and sampling set to 48 cycles per day. Simulated data were collected and analyzed in the same manner as the experimental data (Figure 4D, 4E). Network simulations contained 100 cells with mean field coupling. We then calculated the normalized amplitude and the order parameter defined by Garcia-Ojalvo and colleagues [80]. Codes written in the C programming language are available as Protocol S1.

## Supporting Information

Dataset S1 **Bioluminescence data from 14 individual WT SCN explants.**
(0.68 MB XLS)

Dataset S2 **Bioluminescence data from 16 individual** ***Bmal1*** **^−/−^ SCN explants.**
(0.86 MB XLS)

Dataset S3 **Bioluminescence data from 40 individual** ***Bmal1*** **^−/−^ SCN cells in an intact SCN explant slice.**
(0.31 MB XLS)

Dataset S4 **Bioluminescence data from 40 individual** ***Bmal1*** **^−/−^ SCN cells in an intact SCN explant slice.**
(0.29 MB XLS)

Dataset S5 **Bioluminescence data from 115 individual** ***Bmal1*** **^−/−^ SCN cells dispersed in culture.**
(2.59 MB XLS)

Dataset S6 **Bioluminescence data from 128 individual** ***Bmal1*** **^−/−^ SCN cells dispersed in culture.**
(4.72 MB XLS)

Figure S1 **BMAL1 and BMAL2 transcriptional activity show similar sensitivity to CRY1 repression.** Sensitivity to CRY1 repression was tested by comparing the activities of CLOCK:BMAL1 or CLOCK:BMAL2 complexes in the presence of 0 to 25 ng of *Cry1* plasmid in *Per1-luciferase* reporter assays (as described in [81]). BMAL1 and BMAL2 show similar sensitivity to CRY1-mediated transcriptional repression, suggesting that BMAL2 may account for a low level of activation in the absence of BMAL1. *Bmal2-13*, *-14*, and *-15* represent three independent *Bmal2* clones. For the reporter assay, 293T cells were transiently transfected with Lipofectamine 2000 (Invitrogen) containing 100 ng *Bmal1* or *Bmal2* construct, 100 ng *Clock*, 20 ng *Per1-Luc* reporter, and increasing amounts of *Cry1*.
(0.30 MB TIF)

Figure S2 **Relative** ***Bmal1*** **and** ***Bmal2*** **expression levels in WT and** ***Bmal1*** **^−/−^ tissues −** ***Bmal1*** **and** ***Bmal2*** **mRNA levels were measured using quantitative PCR.** The primers were designed and tested to have consistent amplification efficiency for both *Bmal1* and *Bmal2* template cDNAs. Tissues were collected from animals housed under a light∶dark cycle (12 h light∶12 h dark) at three different time points (zeitgeber time 12, or ZT 12 = onset of dark period). In the case of *Bmal1* ^−/−^ tissues, the mutant transcript level was measured and displayed in the graph. In WT tissues, *Bmal1* clearly shows daily oscillations peaking at ZT18 in the brain and at ZT12 in the liver; however, in *Bmal1* ^−/−^ tissues, the mutant transcript levels are elevated throughout the day and do not show oscillations (presumably due to the absence of *Rev-erb-alpha* repression of *Bmal1*). The total level of *Bmal2* expression is about 10% of the average daily level of *Bmal1* in WT tissues, and its expression is unaffected by *Bmal1* loss-of-function (in *Bmal1* ^−/−^ tissues).
(0.75 MB TIF)

Figure S3 **PER2 levels from simulated cells with varying amounts of BMAL and different coupling mechanisms.** Coupling is critical to understanding the generation of stochastic oscillations in the presence of noise. Therefore, we explored the effectiveness of our hypothesized coupling mechanism in our proposed network model (see Figure 5A). Specifically, we studied the amplitude and order parameter (a measure of the synchronization in a population of cells) as a function of the amount of activator concentration and coupling strength in a population of 100 cells. The order parameter is a mathematical quantity to measure the level of synchronization in a population of cells [80]. It is the ratio of the variance of the averaged ensemble's PER2 (numerator) and the average variance of each individual cell's PER2 in the ensemble (denominator). Furthermore, this parameter is a dimensionless number between 0 (no synchronization, all cells out of phase) and 1 (fully synchronized, all cells in-phase). The coupling strength is a measure of the strength of the inter-cellular coupling strength. It is implemented by changing the production rate of the coupling factor within the model. (A) Amplitude and order parameter surface plots of a population of 100 cells. At right are traces of PER2 levels of simulated cells in the network (blue) along with the population average (yellow); these traces were simulated using the amounts of BMAL and intercellular coupling that produced the strongest amplitudes. The specific values for BMAL and intercellular coupling are BMAL = 1.0, coupling = 0.015. Based on these plots, synchronization occurs over a wide range of parameter values, and therefore the systems behavior is not sensitive to this value. (B) Additional simulations at various ratios of BMAL and coupling strength as noted in individual plots. These values were selected to represent the strongest coupling strength with the lowest BMAL ratio, strongest coupling strength with the highest BMAL ratio, the weakest coupling with the lowest BMAL ratio, the weakest coupling with the highest BMAL ratio, and a level in between. In all simulations, indicated in this figure, the same coupling mechanism is used. We observe that as the strength of the coupling increases in the network, the amplitude of the emergent oscillations also increases.
(0.87 MB TIF)

Figure S4 **Phase portraits of simulated** ***Per2*** **mRNA and simulated PER2 protein.** (A) Phase portraits of PER2 protein and *Per2* mRNA from both WT and *Bmal1* ^−/−^ simulations of SCN network. We plotted *Per2* mRNA against PER2 protein from a WT or *Bmal1* ^−/−^ SCN network simulation. Graphs that are plotted in this manner are called phase portraits, which are geometric trajectories of the dynamical system. In the WT SCN (left panel), we observe a limit cycle in the plane indicative of stable oscillations. Here the phase portraits reveal sustained oscillations, as indicated by the limit cycle in this figure. On the right panel, we plotted *Per2* mRNA against PER2 protein from a *Bmal1* ^−/−^ network simulation. The phase portraits in *Bmal1* ^−/−^ do not reveal clear limit cycles; this fact does not rule out oscillatory behavior and may indicate instead the existence of rhythmic behavior that is aided by the existence of noise in the network. (B) WT and *Bmal1* ^−/−^ SCN network simulations of PER2 protein and *Per2* mRNA as a function of time. On the left panel, we see the changes in WT PER2 protein and *Per2* mRNA concentrations as function of time. As expected, these profiles show oscillations that are approximately 24 h. However, on the right panel, we observe that PER2 protein and *Per2* mRNA concentrations from *Bmal1* ^−/−^ SCN simulations have emergent but stochastic rhythms.
(0.59 MB TIF)

Figure S5 **PER2::LUC bioluminescence from individual** ***Bmal1*** **^−/−^ and WT SCN neurons before, during, and after TTX treatment.** Individual single-cell records before, during, and after TTX treatment from cells within intact *Bmal1* ^−/−^ and WT SCN slices from Figure 6C. Uncoupling cells by TTX treatment within an intact organotypic *Bmal1* ^−/−^ SCN slice results in arrhythmic single-cells with PER2::LUC patterns similar to those of SCN neurons in dispersed culture (see Figure 2F). Under continuous TTX administration (red bar), WT SCN cells are able to maintain PER2::LUC rhythms, although with diminished amplitude. However, when treated with TTX, the *Bmal1* ^−/−^ SCN neurons show an immediate, complete loss of rhythmicity. Upon removal of TTX, the robustness returns to the WT single-cell rhythms and the stochastic rhythmicity returns to *Bmal1* ^−/−^ SCN neurons.
(0.65 MB TIF)

Figure S6 **Uncoupling SCN cells abolishes stochastic rhythms from** ***Bmal1*** **^−/−^ SCN explants.** Representative records of PER2::LUC rhythms of the SCN explants from wild type (blue) and *Bmal1* ^−/−^ (red) mice. Data are shown following a medium change (day 8); shaded area indicates when SCN explants were changed to fresh medium containing vehicle solution (A), Tetrodotoxin (B), Bicuculline (C), and Pertussis toxin (D). (A and B) The effects of vehicle (A) and tetrodotoxin (B) treatments are discussed in the main text of the manuscript (see Figure 6). (C) The application of GABA_A_ receptor antagonist, bicuculline (BIC), effectively blocks GABA-evoked inhibitory postsynaptic currents preventing inter-regional synchronization between dorsal and ventral SCN [45]. Bicuculline application to WT SCN explants showed gradual damping of the PER2::LUC peak-to-trough amplitude; however, it also showed heightened overall luminescence, indicating a higher level of PER2 expression (C, left). The increase in luminescence level was also seen in *Bmal1* ^−/−^ SCN with BIC treatment, but the rhythmic nature of PER2::LUC was eliminated. When BIC was removed, the WT SCN rhythm was immediately restored. *Bmal1* ^−/−^ SCN briefly showed residual effects of BIC and took 1 to 2 d to fully recover its rhythmicity (C, right). (D) Pertussis toxin (PTX) irreversibly inactivates Gi and Go protein activity by preventing their inhibition of adenylyl cyclase; consequently, its application prevents Gi/o-mediated coupling within the SCN. PTX treatment has been shown to both decrease the synchrony among rhythmic neurons and abolishes rhythms in a subset of neurons within the SCN [46]. PTX application to WT SCN significantly reduced the peak-to-trough amplitude leading to damping of rhythms, as was observed with TTX and BIC treatments. *Bmal1* ^−/−^ SCN responded to PTX in a similar fashion as it did to BIC treatment—the PER2::LUC stochastic rhythmicity was completely abolished with an overall increase in luminescence level. Unlike the previous two treatments that were used, rhythms did not fully return when PTX was removed in either WT or *Bmal1* ^−/−^ SCN, likely due to the irreversible nature of PTX. (E) Relative values of PER2::LUC luminescence brightness and FFT power for SCN explants from above (B–D). Bioluminescent counts before, during, and after each of the drug treatments were averaged to obtain Relative Brightness. Spectral analysis (FFT power spectrum, Blackman-Harris windowing) as described previously [78] was used to estimate the amplitude of the circadian frequency range using ClockLab software (Actimetrics, Wilmette, IL). Values for time intervals (±SEM) before, during, and after the treatment are graphed for each genotype and treatment. Relative brightness: there was no significant genotype effect by ANOVA, *F*(1,36) = 0.01, *p*>0.16, but significant drug effect in luminescence level, *F*(2,36) = 0.01, *p*<0.00003; Tukey-Kramer, *p*<0.05. Relative FFT Power: there were both significant genotype and drug effects, *F*(1,45) = 0.01, *p*<0.008, and *F*(2,36) = 0.01, *p*<0.0005, respectively; Tukey-Kramer, *p*<0.05.
(0.76 MB TIF)

Figure S7 **Table of reaction rates and summary of changes.** Listed rate constants are for the stochastic model. Deterministic rate constants can be determined by setting V = 1. Changes from the Forger and Peskin model [14] are highlighted as follows: New reactions are highlighted in green; updated parameters, based on the half-lives reported by Siepka et al. [41], are highlighted in yellow. To match these data, we also increased the rate of formation and transport of mRNA to the cytoplasm (tmc) from the slow rate used in [34] and slightly increased the rate of export of proteins from the nucleus (ne). Since a new mechanism of transcription regulation was used (where just one strong E-box drives transcription of all genes), several of our reaction rates were updated (highlighted in blue) including the maximal rate of transcription of the PER and CRY genes (trPo, trPt, trRo, trRt), the translation rate of PER (tlp), and the concentration of the kinases (Ct) to match the data reported by Lee et al. [11] on the relative concentration of clock proteins. The new concentrations in the model can be converted to number of molecules equivalent to those reported in [34] by choosing V = 200.
(0.92 MB TIF)

Protocol S1 **Code for the stochastic mathematical simulation of the circadian molecular clock; the code is written in the C programming language.** This code was used to generate simulations found in Figures 3B, 4A, 4D, 4E, 5B, 5C, 5D, 5F, 6D, 8C, S3, and S4. The parameters, and a brief description, used in this code can be found in Figure S7.
(0.05 MB DOC)

Protocol S2 **Description of model equations in human readable format.** The equations for this model, parameters, and a brief description can be found in Figure S7. The equations were simulated in MatLab and were used to generate Figures 3D, 4B, 4C, 5D, 5E, and 5F.
(0.18 MB DOC)

Text S1 **Supplementary methods.**
(0.06 MB DOC)

Video S1 **Bioluminescence expression patterns from** ***Bmal1*** **^−/−^ SCN explants.**
(1.36 MB MOV)

Video S2 **Bioluminescence expression patterns from** ***Bmal1*** **^−/−^ dissociated SCN neurons.**
(1.48 MB MOV)

## Abbreviations

CA: coupling agent
cAMP: adenosine 3′,5′-monophosphate
CRE: cAMP response element
CREB: CRE-binding protein
FFT: Fast Fourier transform
LUC: luciferase
*Per2*: *Period2*
SCN: suprachiasmatic nucleus
TTX: tetrodotoxin
VIP: vasoactive intestinal polypeptide
WT: wild type
