# Supplementary figures and images for: Emergence of Noise-Induced Oscillations in the Central Circadian Pacemaker
Source: PLoS Biol. 2010 Oct 12;8(10):e1000513. doi: 10.1371/journal.pbio.1000513 (PMC2953532; doi:10.1371/journal.pbio.1000513)

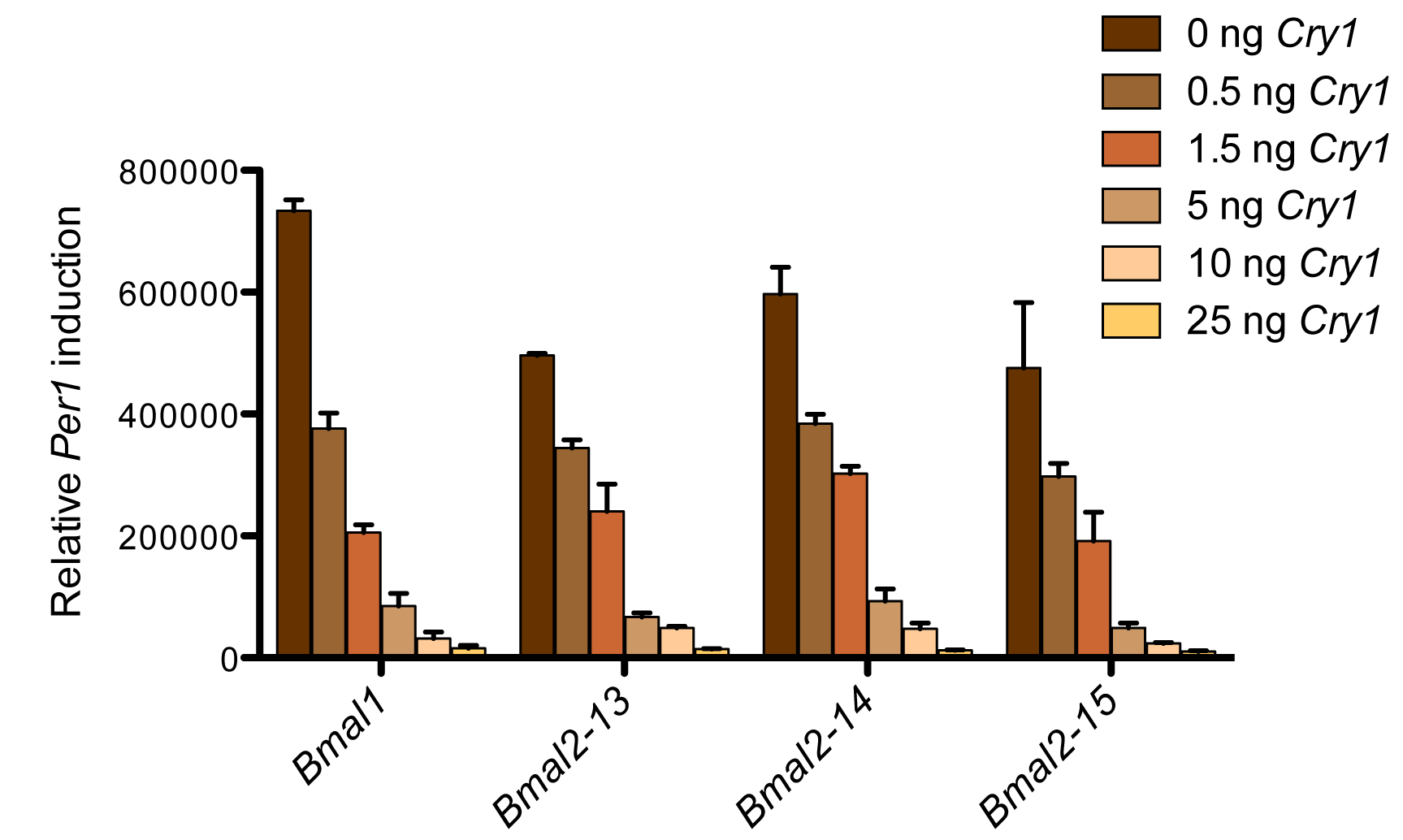

Supplement: Figure S1 — BMAL1 and BMAL2 transcriptional activity show similar sensitivity to CRY1 repression. Sensitivity to CRY1 repression was tested by comparing the activities of CLOCK:BMAL1 or CLOCK:BMAL2 complexes in the presence of 0 to 25 ng of Cry1 plasmid in Per1-luciferase reporter assays (as described in [81]). BMAL1 and BMAL2 show similar sensitivity to CRY1-mediated transcriptional repression, suggesting that BMAL2 may account for a low level of activation in the absence of BMAL1. Bmal2-13, -14, and -15 represent three independent Bmal2 clones. For the reporter assay, 293T cells were transiently transfected with Lipofectamine 2000 (Invitrogen) containing 100 ng Bmal1 or Bmal2 construct, 100 ng Clock, 20 ng Per1-Luc reporter, and increasing amounts of Cry1. (0.30 MB TIF) [file pbio.1000513.s007.tif]

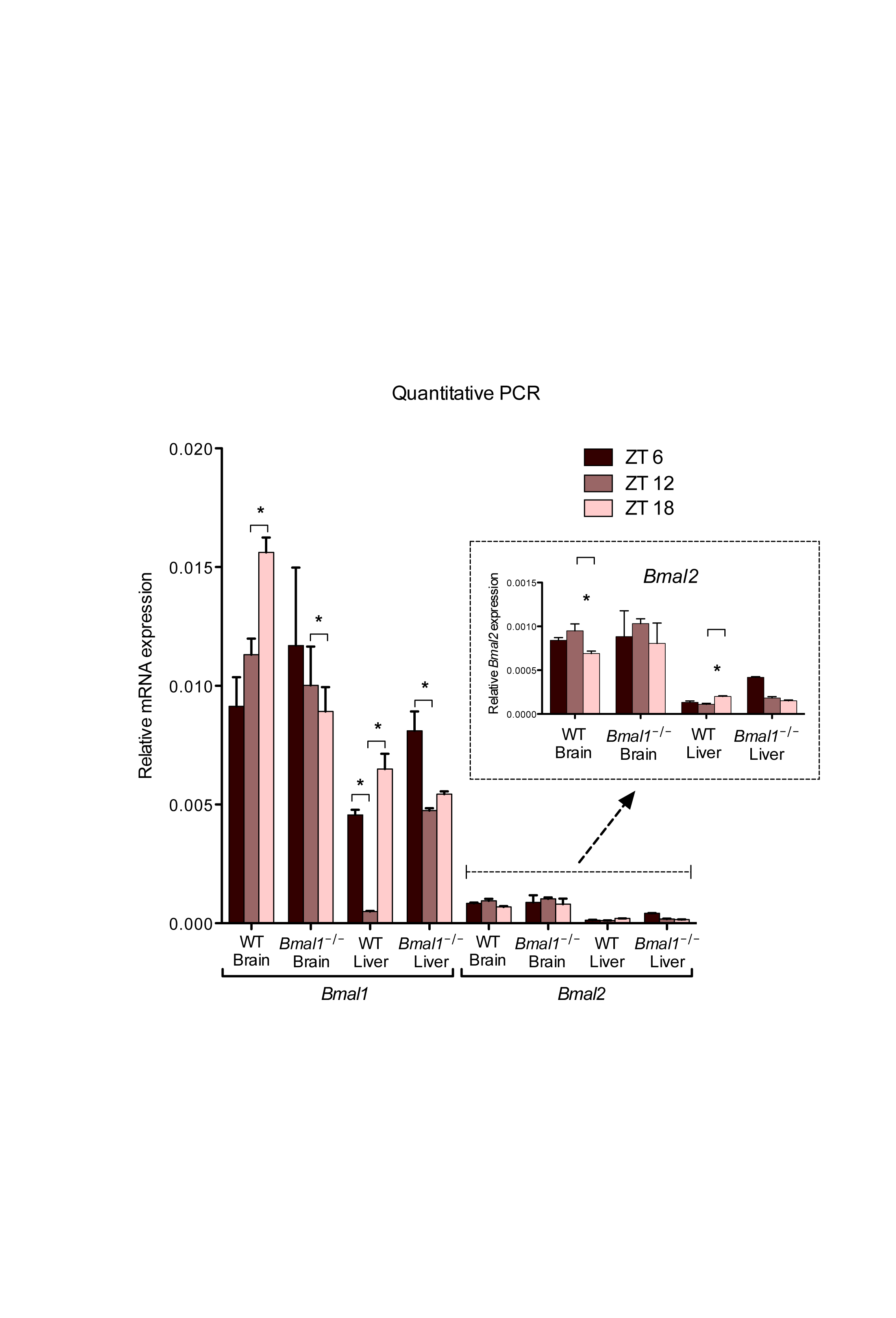

Supplement: Figure S2 — Relative Bmal1 and Bmal2 expression levels in WT and Bmal1 −/− tissues − Bmal1 and Bmal2 mRNA levels were measured using quantitative PCR. The primers were designed and tested to have consistent amplification efficiency for both Bmal1 and Bmal2 template cDNAs. Tissues were collected from animals housed under a light∶dark cycle (12 h light∶12 h dark) at three different time points (zeitgeber time 12, or ZT 12 = onset of dark period). In the case of Bmal1 −/− tissues, the mutant transcript level was measured and displayed in the graph. In WT tissues, Bmal1 clearly shows daily oscillations peaking at ZT18 in the brain and at ZT12 in the liver; however, in Bmal1 −/− tissues, the mutant transcript levels are elevated throughout the day and do not show oscillations (presumably due to the absence of Rev-erb-alpha repression of Bmal1). The total level of Bmal2 expression is about 10% of the average daily level of Bmal1 in WT tissues, and its expression is unaffected by Bmal1 loss-of-function (in Bmal1 −/− tissues). (0.75 MB TIF) [file pbio.1000513.s008.tif]

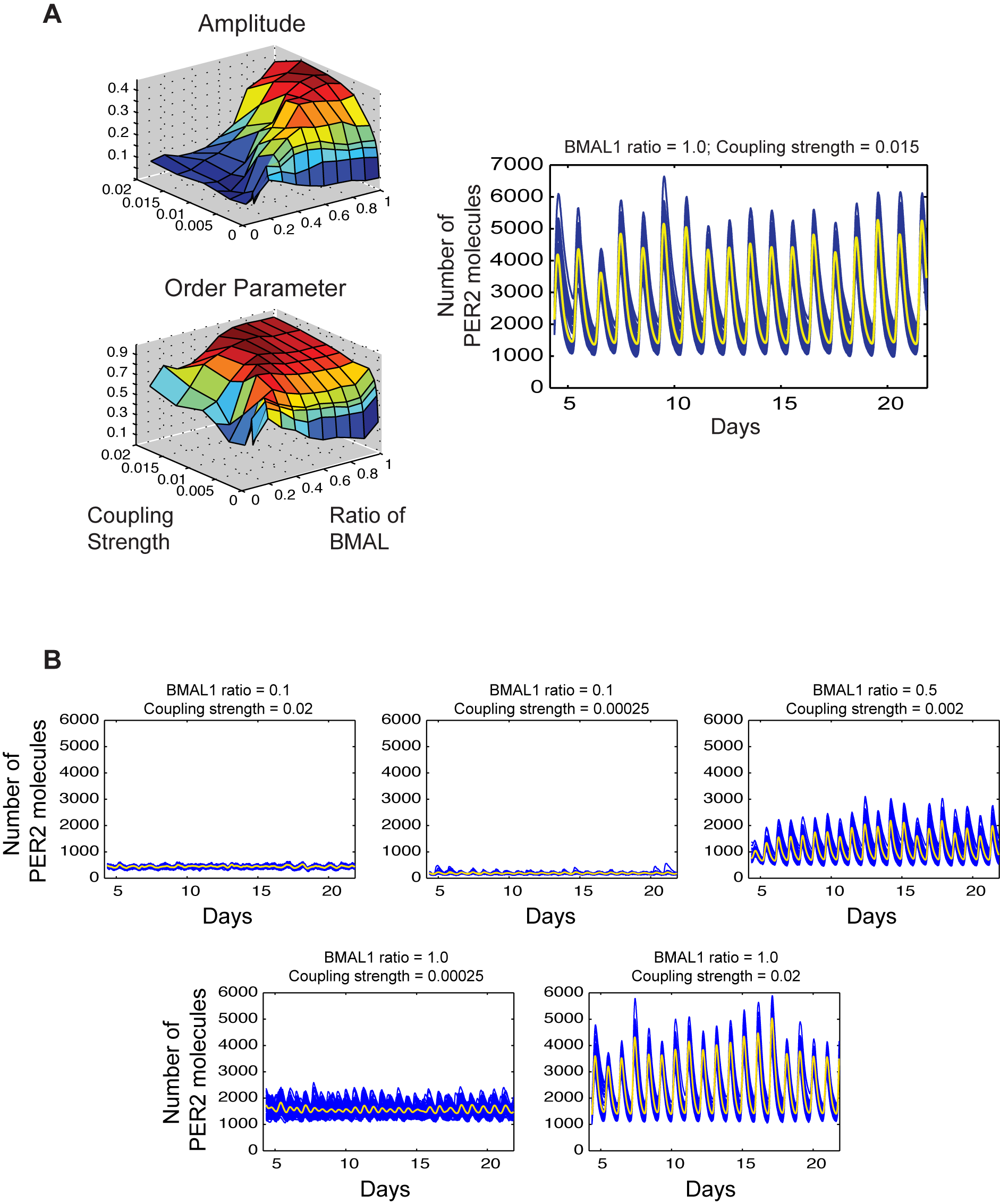

Supplement: Figure S3 — PER2 levels from simulated cells with varying amounts of BMAL and different coupling mechanisms. Coupling is critical to understanding the generation of stochastic oscillations in the presence of noise. Therefore, we explored the effectiveness of our hypothesized coupling mechanism in our proposed network model (see Figure 5A). Specifically, we studied the amplitude and order parameter (a measure of the synchronization in a population of cells) as a function of the amount of activator concentration and coupling strength in a population of 100 cells. The order parameter is a mathematical quantity to measure the level of synchronization in a population of cells [80]. It is the ratio of the variance of the averaged ensemble's PER2 (numerator) and the average variance of each individual cell's PER2 in the ensemble (denominator). Furthermore, this parameter is a dimensionless number between 0 (no synchronization, all cells out of phase) and 1 (fully synchronized, all cells in-phase). The coupling strength is a measure of the strength of the inter-cellular coupling strength. It is implemented by changing the production rate of the coupling factor within the model. (A) Amplitude and order parameter surface plots of a population of 100 cells. At right are traces of PER2 levels of simulated cells in the network (blue) along with the population average (yellow); these traces were simulated using the amounts of BMAL and intercellular coupling that produced the strongest amplitudes. The specific values for BMAL and intercellular coupling are BMAL = 1.0, coupling = 0.015. Based on these plots, synchronization occurs over a wide range of parameter values, and therefore the systems behavior is not sensitive to this value. (B) Additional simulations at various ratios of BMAL and coupling strength as noted in individual plots. These values were selected to represent the strongest coupling strength with the lowest BMAL ratio, strongest coupling strength with the highest BMAL ratio, t [file pbio.1000513.s009.tif]

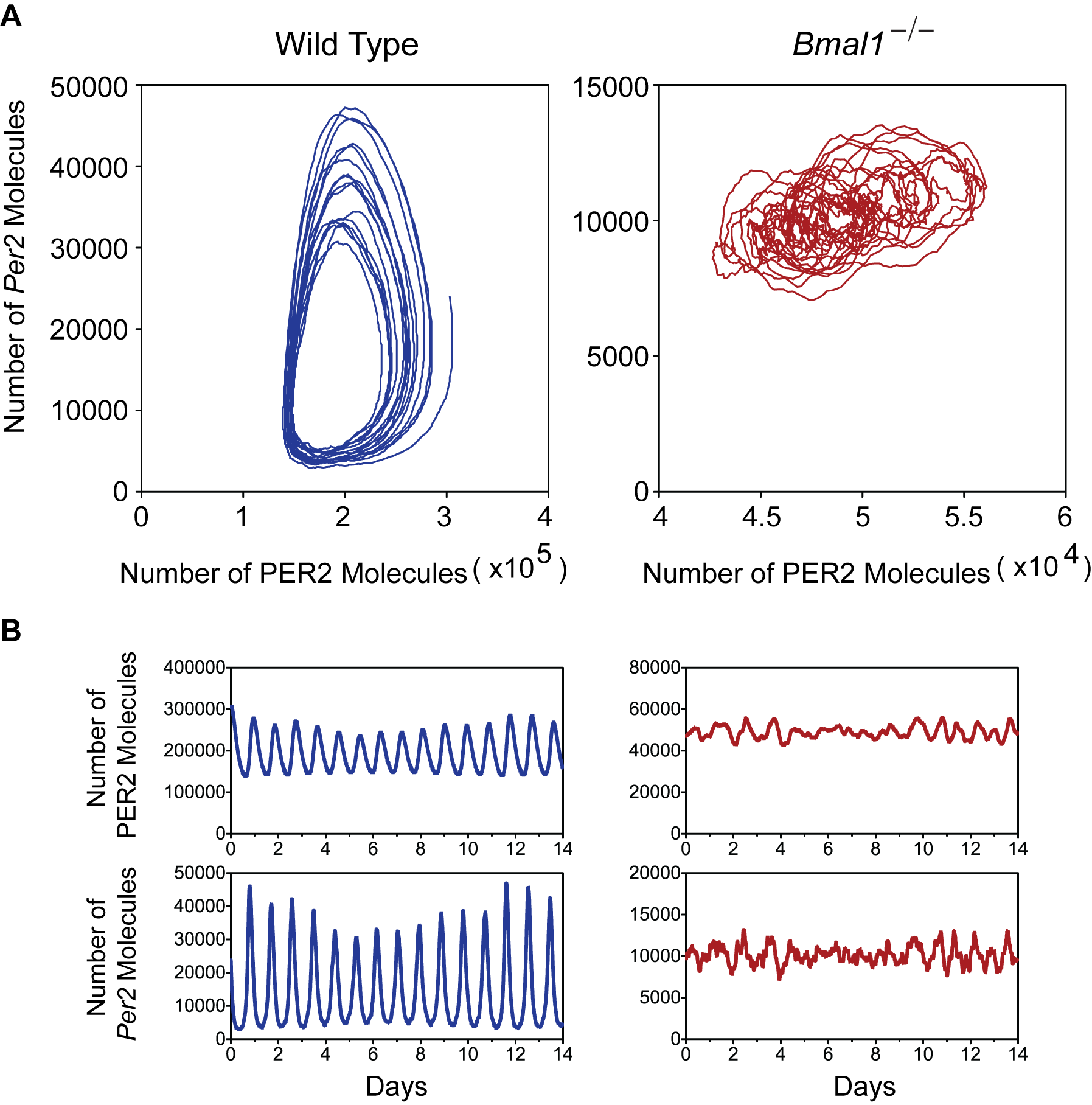

Supplement: Figure S4 — Phase portraits of simulated Per2 mRNA and simulated PER2 protein. (A) Phase portraits of PER2 protein and Per2 mRNA from both WT and Bmal1 −/− simulations of SCN network. We plotted Per2 mRNA against PER2 protein from a WT or Bmal1 −/− SCN network simulation. Graphs that are plotted in this manner are called phase portraits, which are geometric trajectories of the dynamical system. In the WT SCN (left panel), we observe a limit cycle in the plane indicative of stable oscillations. Here the phase portraits reveal sustained oscillations, as indicated by the limit cycle in this figure. On the right panel, we plotted Per2 mRNA against PER2 protein from a Bmal1 −/− network simulation. The phase portraits in Bmal1 −/− do not reveal clear limit cycles; this fact does not rule out oscillatory behavior and may indicate instead the existence of rhythmic behavior that is aided by the existence of noise in the network. (B) WT and Bmal1 −/− SCN network simulations of PER2 protein and Per2 mRNA as a function of time. On the left panel, we see the changes in WT PER2 protein and Per2 mRNA concentrations as function of time. As expected, these profiles show oscillations that are approximately 24 h. However, on the right panel, we observe that PER2 protein and Per2 mRNA concentrations from Bmal1 −/− SCN simulations have emergent but stochastic rhythms. (0.59 MB TIF) [file pbio.1000513.s010.tif]

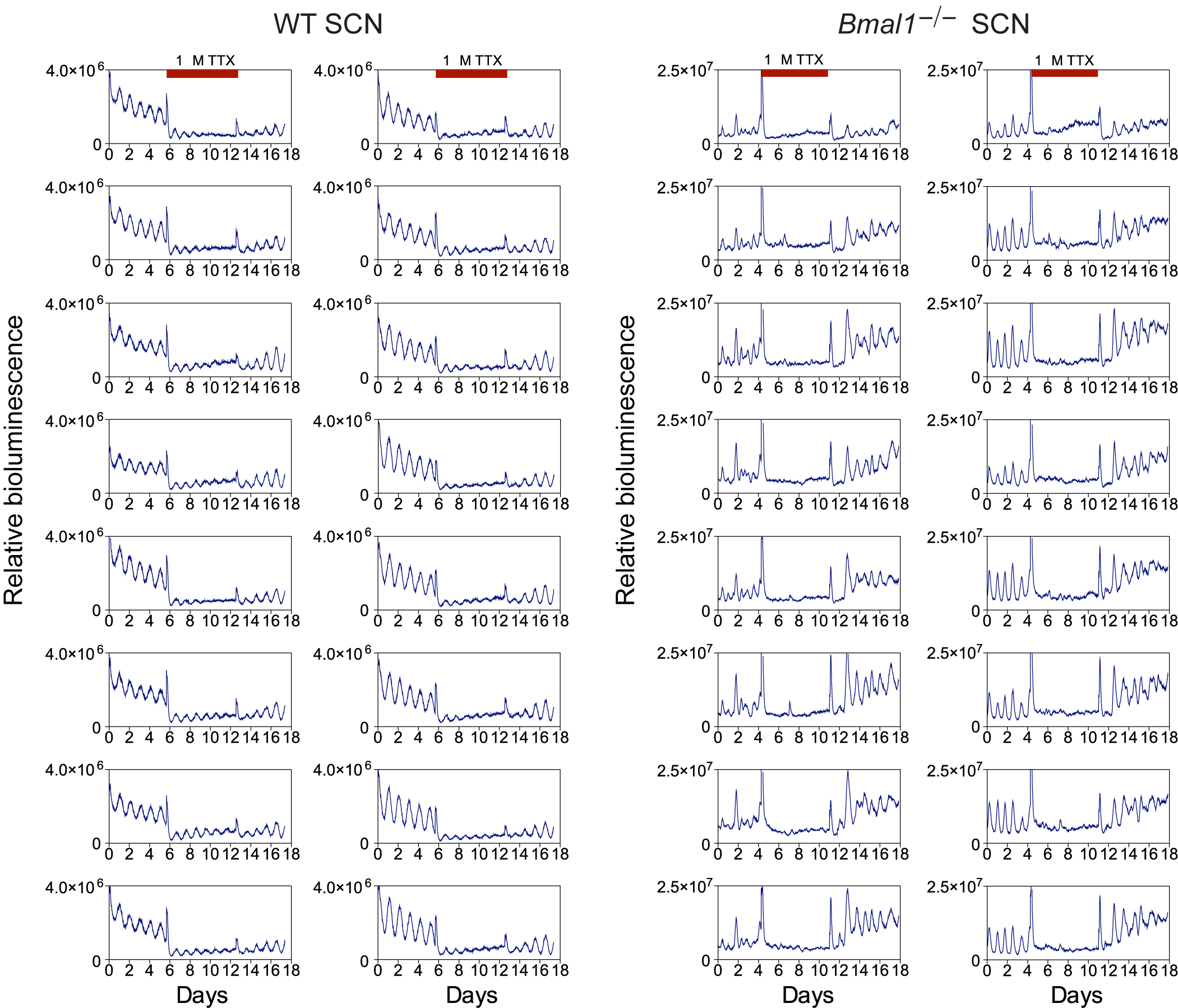

Supplement: Figure S5 — PER2::LUC bioluminescence from individual Bmal1 −/− and WT SCN neurons before, during, and after TTX treatment. Individual single-cell records before, during, and after TTX treatment from cells within intact Bmal1 −/− and WT SCN slices from Figure 6C. Uncoupling cells by TTX treatment within an intact organotypic Bmal1 −/− SCN slice results in arrhythmic single-cells with PER2::LUC patterns similar to those of SCN neurons in dispersed culture (see Figure 2F). Under continuous TTX administration (red bar), WT SCN cells are able to maintain PER2::LUC rhythms, although with diminished amplitude. However, when treated with TTX, the Bmal1 −/− SCN neurons show an immediate, complete loss of rhythmicity. Upon removal of TTX, the robustness returns to the WT single-cell rhythms and the stochastic rhythmicity returns to Bmal1 −/− SCN neurons. (0.65 MB TIF) [file pbio.1000513.s011.tif]

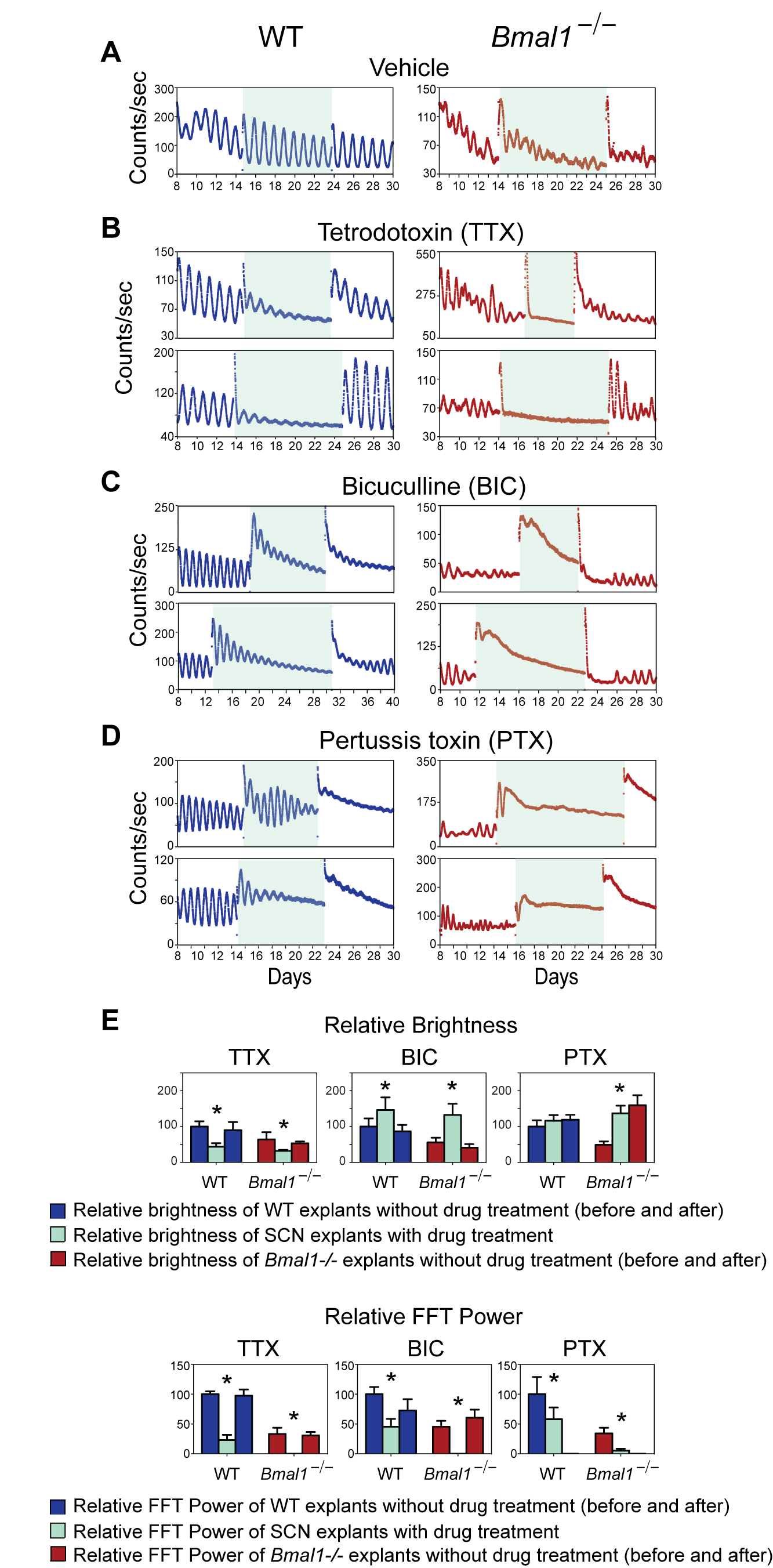

Supplement: Figure S6 — Uncoupling SCN cells abolishes stochastic rhythms from Bmal1 −/− SCN explants. Representative records of PER2::LUC rhythms of the SCN explants from wild type (blue) and Bmal1 −/− (red) mice. Data are shown following a medium change (day 8); shaded area indicates when SCN explants were changed to fresh medium containing vehicle solution (A), Tetrodotoxin (B), Bicuculline (C), and Pertussis toxin (D). (A and B) The effects of vehicle (A) and tetrodotoxin (B) treatments are discussed in the main text of the manuscript (see Figure 6). (C) The application of GABAA receptor antagonist, bicuculline (BIC), effectively blocks GABA-evoked inhibitory postsynaptic currents preventing inter-regional synchronization between dorsal and ventral SCN [45]. Bicuculline application to WT SCN explants showed gradual damping of the PER2::LUC peak-to-trough amplitude; however, it also showed heightened overall luminescence, indicating a higher level of PER2 expression (C, left). The increase in luminescence level was also seen in Bmal1 −/− SCN with BIC treatment, but the rhythmic nature of PER2::LUC was eliminated. When BIC was removed, the WT SCN rhythm was immediately restored. Bmal1 −/− SCN briefly showed residual effects of BIC and took 1 to 2 d to fully recover its rhythmicity (C, right). (D) Pertussis toxin (PTX) irreversibly inactivates Gi and Go protein activity by preventing their inhibition of adenylyl cyclase; consequently, its application prevents Gi/o-mediated coupling within the SCN. PTX treatment has been shown to both decrease the synchrony among rhythmic neurons and abolishes rhythms in a subset of neurons within the SCN [46]. PTX application to WT SCN significantly reduced the peak-to-trough amplitude leading to damping of rhythms, as was observed with TTX and BIC treatments. Bmal1 −/− SCN responded to PTX in a similar fashion as it did to BIC treatment—the PER2::LUC stochastic rhythmicity was completely abolished with an overall increase in luminescence level. Unlike th [file pbio.1000513.s012.tif]

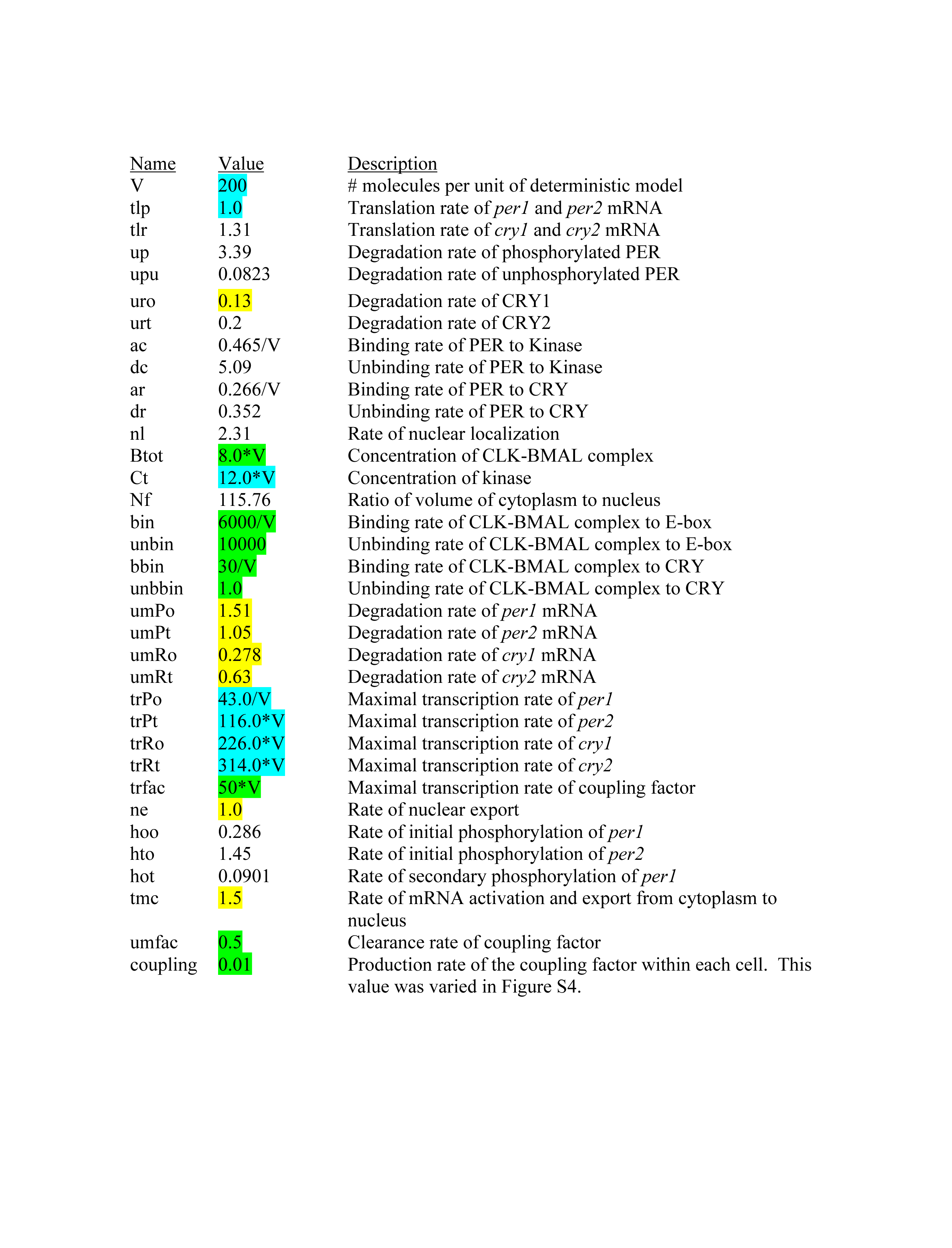

Supplement: Figure S7 — Table of reaction rates and summary of changes. Listed rate constants are for the stochastic model. Deterministic rate constants can be determined by setting V = 1. Changes from the Forger and Peskin model [14] are highlighted as follows: New reactions are highlighted in green; updated parameters, based on the half-lives reported by Siepka et al. [41], are highlighted in yellow. To match these data, we also increased the rate of formation and transport of mRNA to the cytoplasm (tmc) from the slow rate used in [34] and slightly increased the rate of export of proteins from the nucleus (ne). Since a new mechanism of transcription regulation was used (where just one strong E-box drives transcription of all genes), several of our reaction rates were updated (highlighted in blue) including the maximal rate of transcription of the PER and CRY genes (trPo, trPt, trRo, trRt), the translation rate of PER (tlp), and the concentration of the kinases (Ct) to match the data reported by Lee et al. [11] on the relative concentration of clock proteins. The new concentrations in the model can be converted to number of molecules equivalent to those reported in [34] by choosing V = 200. (0.92 MB TIF) [file pbio.1000513.s013.tif]
