# Supplementary material for: Emergence of Noise-Induced Oscillations in the Central Circadian Pacemaker
Source: PLoS Biol. 2010 Oct 12;8(10):e1000513. doi: 10.1371/journal.pbio.1000513 (PMC2953532; doi:10.1371/journal.pbio.1000513)
Supplement: Protocol S2 — Description of model equations in human readable format. The equations for this model, parameters, and a brief description can be found in Figure S7. The equations were simulated in MatLab and were used to generate Figures 3D, 4B, 4C, 5D, 5E, and 5F. (0.18 MB DOC) [file pbio.1000513.s015.doc]

Variables

MnPo Per1 mRNA (nucleus)

MnPt Per2 mRNA (nucleus)

MnRo Cry1 mRNA (nucleus)

MnRt Cry2 mRNA (nucleus)

McPo Per1 mRNA (cytoplasm)

McPt Per2 mRNA (cytoplasm)

McRo Cry1 mRNA (cytoplasm)

McRt Cry2 mRNA (cytoplasm)

x[j][k][l][m][n] Various states of Complex

j = 0 no PER

j = 1 unphosphorylated PER1

j = 2 PER1 with initial Phosphorylation level

j = 3 PER1 with secondary Phosphorylation level

j = 4 PER2 with both phosphorylation levels

j = 5 unphosphorylated PER2

j = 6 phosphorylationed PER2

k = 0 no CRY

k = 1 CRY1

k = 2 CRY2

l = 0 no Kinase

l = 1 Kinase

m = 0 cytoplasm

m = 1 nucleus

n = 0 no BMAL

n = 1 BMAL1

n = 2 BMAL2

facr Factor protein

G Probability of Free E-box

GR Probability of Repressed E-box

For each simulation (expect in the case of the bifurcation diagram in Figure 3, parameters were chosen from a Gaussian distribution (see Figure S1).

Equations

dGR[t]/dt=-unbin GR[t]+bin (1-G[t]-GR[t]) (x[0][1][0][1][1][t]+x[0][1][0][1][2][t]+x[0][1][1][1][1][t]+x[0][1][1][1][2][t]+x[0][2][0][1][1][t]+x[0][2][0][1][2][t]+x[0][2][1][1][1][t]+x[0][2][1][1][2][t]+x[2][1][0][1][1][t]+x[2][1][0][1][2][t]+x[2][1][1][1][1][t]+x[2][1][1][1][2][t]+x[2][2][0][1][1][t]+x[2][2][0][1][2][t]+x[2][2][1][1][1][t]+x[2][2][1][1][2][t]+x[4][1][0][1][1][t]+x[4][1][0][1][2][t]+x[4][1][1][1][1][t]+x[4][1][1][1][2][t]+x[4][2][0][1][1][t]+x[4][2][0][1][2][t]+x[4][2][1][1][1][t]+x[4][2][1][1][2][t]+x[6][1][0][1][1][t]+x[6][1][0][1][2][t]+x[6][1][1][1][1][t]+x[6][1][1][1][2][t]+x[6][2][0][1][1][t]+x[6][2][0][1][2][t]+x[6][2][1][1][1][t]+x[6][2][1][1][2][t])

dG[t]/dt=-unbin G[t]+bin (1-G[t]-GR[t]) (x[0][0][0][1][1][t]+x[0][0][0][1][2][t])

dfacr[t]/dt=-umfac facr[t]+trfac G[t]

dMnRo[t]/dt=trRo G[t]-tmc MnRo[t]

dMcRo[t]/dt=-umRo McRo[t]+tmc MnRo[t]

dMnRt[t]/dt=trRt G[t]-tmc MnRt[t]

dMcRt[t]/dt=-umRt McRt[t]+tmc MnRt[t]

dMnPo[t]/dt=trPo G[t]+coupfac facr[t] (1-GR[t])-tmc MnPo[t]

dMcPo[t]/dt=-umPo McPo[t]+tmc MnPo[t]

dMnPt[t]/dt=trPt G[t]+coupfac facr[t] (1-GR[t])-tmc MnPt[t]

dMcPt[t]/dt=-umPt McPt[t]+tmc MnPt[t]

dx[0][0][0][0][0][t]/dt=0

(When a derivative = 0, this indicates a complex that cannot form, for example as the above complex has nothing in it).

dx[0][0][0][0][1][t]/dt=-bbin x[0][0][0][0][1][t] x[0][1][0][0][0][t]+unbbin x[0][1][0][0][1][t]+uro x[0][1][0][0][1][t]-bbin x[0][0][0][0][1][t] x[0][1][1][0][0][t]+unbbin x[0][1][1][0][1][t]-bbin x[0][0][0][0][1][t] x[0][2][0][0][0][t]+unbbin x[0][2][0][0][1][t]+urt x[0][2][0][0][1][t]-bbin x[0][0][0][0][1][t] x[0][2][1][0][0][t]+unbbin x[0][2][1][0][1][t]-bbin x[0][0][0][0][1][t] x[2][1][0][0][0][t]+unbbin x[2][1][0][0][1][t]-bbin x[0][0][0][0][1][t] x[2][1][1][0][0][t]+unbbin x[2][1][1][0][1][t]-bbin x[0][0][0][0][1][t] x[2][2][0][0][0][t]+unbbin x[2][2][0][0][1][t]-bbin x[0][0][0][0][1][t] x[2][2][1][0][0][t]+unbbin x[2][2][1][0][1][t]-bbin x[0][0][0][0][1][t] x[4][1][0][0][0][t]+unbbin x[4][1][0][0][1][t]-bbin x[0][0][0][0][1][t] x[4][1][1][0][0][t]+unbbin x[4][1][1][0][1][t]-bbin x[0][0][0][0][1][t] x[4][2][0][0][0][t]+unbbin x[4][2][0][0][1][t]-bbin x[0][0][0][0][1][t] x[4][2][1][0][0][t]+unbbin x[4][2][1][0][1][t]-bbin x[0][0][0][0][1][t] x[6][1][0][0][0][t]+unbbin x[6][1][0][0][1][t]-bbin x[0][0][0][0][1][t] x[6][1][1][0][0][t]+unbbin x[6][1][1][0][1][t]-bbin x[0][0][0][0][1][t] x[6][2][0][0][0][t]+unbbin x[6][2][0][0][1][t]-bbin x[0][0][0][0][1][t] x[6][2][1][0][0][t]+unbbin x[6][2][1][0][1][t]

dx[0][0][0][0][2][t]/dt=-bbin x[0][0][0][0][2][t] x[0][1][0][0][0][t]+unbbin x[0][1][0][0][2][t]+uro x[0][1][0][0][2][t]-bbin x[0][0][0][0][2][t] x[0][1][1][0][0][t]+unbbin x[0][1][1][0][2][t]-bbin x[0][0][0][0][2][t] x[0][2][0][0][0][t]+unbbin x[0][2][0][0][2][t]+urt x[0][2][0][0][2][t]-bbin x[0][0][0][0][2][t] x[0][2][1][0][0][t]+unbbin x[0][2][1][0][2][t]-bbin x[0][0][0][0][2][t] x[2][1][0][0][0][t]+unbbin x[2][1][0][0][2][t]-bbin x[0][0][0][0][2][t] x[2][1][1][0][0][t]+unbbin x[2][1][1][0][2][t]-bbin x[0][0][0][0][2][t] x[2][2][0][0][0][t]+unbbin x[2][2][0][0][2][t]-bbin x[0][0][0][0][2][t] x[2][2][1][0][0][t]+unbbin x[2][2][1][0][2][t]-bbin x[0][0][0][0][2][t] x[4][1][0][0][0][t]+unbbin x[4][1][0][0][2][t]-bbin x[0][0][0][0][2][t] x[4][1][1][0][0][t]+unbbin x[4][1][1][0][2][t]-bbin x[0][0][0][0][2][t] x[4][2][0][0][0][t]+unbbin x[4][2][0][0][2][t]-bbin x[0][0][0][0][2][t] x[4][2][1][0][0][t]+unbbin x[4][2][1][0][2][t]-bbin x[0][0][0][0][2][t] x[6][1][0][0][0][t]+unbbin x[6][1][0][0][2][t]-bbin x[0][0][0][0][2][t] x[6][1][1][0][0][t]+unbbin x[6][1][1][0][2][t]-bbin x[0][0][0][0][2][t] x[6][2][0][0][0][t]+unbbin x[6][2][0][0][2][t]-bbin x[0][0][0][0][2][t] x[6][2][1][0][0][t]+unbbin x[6][2][1][0][2][t]

dx[0][0][0][1][0][t]/dt=0

dx[0][0][0][1][1][t]/dt=-bbin Nf x[0][0][0][1][1][t] x[0][1][0][1][0][t]+unbbin x[0][1][0][1][1][t]+uro x[0][1][0][1][1][t]-bbin Nf x[0][0][0][1][1][t] x[0][1][1][1][0][t]+unbbin x[0][1][1][1][1][t]-bbin Nf x[0][0][0][1][1][t] x[0][2][0][1][0][t]+unbbin x[0][2][0][1][1][t]+urt x[0][2][0][1][1][t]-bbin Nf x[0][0][0][1][1][t] x[0][2][1][1][0][t]+unbbin x[0][2][1][1][1][t]-bbin Nf x[0][0][0][1][1][t] x[2][1][0][1][0][t]+unbbin x[2][1][0][1][1][t]-bbin Nf x[0][0][0][1][1][t] x[2][1][1][1][0][t]+unbbin x[2][1][1][1][1][t]-bbin Nf x[0][0][0][1][1][t] x[2][2][0][1][0][t]+unbbin x[2][2][0][1][1][t]-bbin Nf x[0][0][0][1][1][t] x[2][2][1][1][0][t]+unbbin x[2][2][1][1][1][t]-bbin Nf x[0][0][0][1][1][t] x[4][1][0][1][0][t]+unbbin x[4][1][0][1][1][t]-bbin Nf x[0][0][0][1][1][t] x[4][1][1][1][0][t]+unbbin x[4][1][1][1][1][t]-bbin Nf x[0][0][0][1][1][t] x[4][2][0][1][0][t]+unbbin x[4][2][0][1][1][t]-bbin Nf x[0][0][0][1][1][t] x[4][2][1][1][0][t]+unbbin x[4][2][1][1][1][t]-bbin Nf x[0][0][0][1][1][t] x[6][1][0][1][0][t]+unbbin x[6][1][0][1][1][t]-bbin Nf x[0][0][0][1][1][t] x[6][1][1][1][0][t]+unbbin x[6][1][1][1][1][t]-bbin Nf x[0][0][0][1][1][t] x[6][2][0][1][0][t]+unbbin x[6][2][0][1][1][t]-bbin Nf x[0][0][0][1][1][t] x[6][2][1][1][0][t]+unbbin x[6][2][1][1][1][t]

dx[0][0][0][1][2][t]/dt=-bbin Nf x[0][0][0][1][2][t] x[0][1][0][1][0][t]+unbbin x[0][1][0][1][2][t]+uro x[0][1][0][1][2][t]-bbin Nf x[0][0][0][1][2][t] x[0][1][1][1][0][t]+unbbin x[0][1][1][1][2][t]-bbin Nf x[0][0][0][1][2][t] x[0][2][0][1][0][t]+unbbin x[0][2][0][1][2][t]+urt x[0][2][0][1][2][t]-bbin Nf x[0][0][0][1][2][t] x[0][2][1][1][0][t]+unbbin x[0][2][1][1][2][t]-bbin Nf x[0][0][0][1][2][t] x[2][1][0][1][0][t]+unbbin x[2][1][0][1][2][t]-bbin Nf x[0][0][0][1][2][t] x[2][1][1][1][0][t]+unbbin x[2][1][1][1][2][t]-bbin Nf x[0][0][0][1][2][t] x[2][2][0][1][0][t]+unbbin x[2][2][0][1][2][t]-bbin Nf x[0][0][0][1][2][t] x[2][2][1][1][0][t]+unbbin x[2][2][1][1][2][t]-bbin Nf x[0][0][0][1][2][t] x[4][1][0][1][0][t]+unbbin x[4][1][0][1][2][t]-bbin Nf x[0][0][0][1][2][t] x[4][1][1][1][0][t]+unbbin x[4][1][1][1][2][t]-bbin Nf x[0][0][0][1][2][t] x[4][2][0][1][0][t]+unbbin x[4][2][0][1][2][t]-bbin Nf x[0][0][0][1][2][t] x[4][2][1][1][0][t]+unbbin x[4][2][1][1][2][t]-bbin Nf x[0][0][0][1][2][t] x[6][1][0][1][0][t]+unbbin x[6][1][0][1][2][t]-bbin Nf x[0][0][0][1][2][t] x[6][1][1][1][0][t]+unbbin x[6][1][1][1][2][t]-bbin Nf x[0][0][0][1][2][t] x[6][2][0][1][0][t]+unbbin x[6][2][0][1][2][t]-bbin Nf x[0][0][0][1][2][t] x[6][2][1][1][0][t]+unbbin x[6][2][1][1][2][t]

dx[0][0][1][0][0][t]/dt=-ac x[0][0][1][0][0][t] x[1][0][0][0][0][t]-ac x[0][0][1][0][0][t] x[1][0][0][0][1][t]-ac x[0][0][1][0][0][t] x[1][0][0][0][2][t]+dc x[1][0][1][0][0][t]+upu x[1][0][1][0][0][t]+dc x[1][0][1][0][1][t]+dc x[1][0][1][0][2][t]-ac x[0][0][1][0][0][t] x[1][1][0][0][0][t]-ac x[0][0][1][0][0][t] x[1][1][0][0][1][t]-ac x[0][0][1][0][0][t] x[1][1][0][0][2][t]+dc x[1][1][1][0][0][t]+dc x[1][1][1][0][1][t]+dc x[1][1][1][0][2][t]-ac x[0][0][1][0][0][t] x[1][2][0][0][0][t]-ac x[0][0][1][0][0][t] x[1][2][0][0][1][t]-ac x[0][0][1][0][0][t] x[1][2][0][0][2][t]+dc x[1][2][1][0][0][t]+dc x[1][2][1][0][1][t]+dc x[1][2][1][0][2][t]-ac x[0][0][1][0][0][t] x[2][0][0][0][0][t]-ac x[0][0][1][0][0][t] x[2][0][0][0][1][t]-ac x[0][0][1][0][0][t] x[2][0][0][0][2][t]+dc x[2][0][1][0][0][t]+up x[2][0][1][0][0][t]+dc x[2][0][1][0][1][t]+dc x[2][0][1][0][2][t]-ac x[0][0][1][0][0][t] x[2][1][0][0][0][t]-ac x[0][0][1][0][0][t] x[2][1][0][0][1][t]-ac x[0][0][1][0][0][t] x[2][1][0][0][2][t]+dc x[2][1][1][0][0][t]+dc x[2][1][1][0][1][t]+dc x[2][1][1][0][2][t]-ac x[0][0][1][0][0][t] x[2][2][0][0][0][t]-ac x[0][0][1][0][0][t] x[2][2][0][0][1][t]-ac x[0][0][1][0][0][t] x[2][2][0][0][2][t]+dc x[2][2][1][0][0][t]+dc x[2][2][1][0][1][t]+dc x[2][2][1][0][2][t]-ac x[0][0][1][0][0][t] x[3][0][0][0][0][t]-ac x[0][0][1][0][0][t] x[3][0][0][0][1][t]-ac x[0][0][1][0][0][t] x[3][0][0][0][2][t]+dc x[3][0][1][0][0][t]+upu x[3][0][1][0][0][t]+dc x[3][0][1][0][1][t]+dc x[3][0][1][0][2][t]-ac x[0][0][1][0][0][t] x[3][1][0][0][0][t]-ac x[0][0][1][0][0][t] x[3][1][0][0][1][t]-ac x[0][0][1][0][0][t] x[3][1][0][0][2][t]+dc x[3][1][1][0][0][t]+dc x[3][1][1][0][1][t]+dc x[3][1][1][0][2][t]-ac x[0][0][1][0][0][t] x[3][2][0][0][0][t]-ac x[0][0][1][0][0][t] x[3][2][0][0][1][t]-ac x[0][0][1][0][0][t] x[3][2][0][0][2][t]+dc x[3][2][1][0][0][t]+dc x[3][2][1][0][1][t]+dc x[3][2][1][0][2][t]-ac x[0][0][1][0][0][t] x[4][0][0][0][0][t]-ac x[0][0][1][0][0][t] x[4][0][0][0][1][t]-ac x[0][0][1][0][0][t] x[4][0][0][0][2][t]+dc x[4][0][1][0][0][t]+up x[4][0][1][0][0][t]+dc x[4][0][1][0][1][t]+dc x[4][0][1][0][2][t]-ac x[0][0][1][0][0][t] x[4][1][0][0][0][t]-ac x[0][0][1][0][0][t] x[4][1][0][0][1][t]-ac x[0][0][1][0][0][t] x[4][1][0][0][2][t]+dc x[4][1][1][0][0][t]+dc x[4][1][1][0][1][t]+dc x[4][1][1][0][2][t]-ac x[0][0][1][0][0][t] x[4][2][0][0][0][t]-ac x[0][0][1][0][0][t] x[4][2][0][0][1][t]-ac x[0][0][1][0][0][t] x[4][2][0][0][2][t]+dc x[4][2][1][0][0][t]+dc x[4][2][1][0][1][t]+dc x[4][2][1][0][2][t]-ac x[0][0][1][0][0][t] x[5][0][0][0][0][t]-ac x[0][0][1][0][0][t] x[5][0][0][0][1][t]-ac x[0][0][1][0][0][t] x[5][0][0][0][2][t]+dc x[5][0][1][0][0][t]+upu x[5][0][1][0][0][t]+dc x[5][0][1][0][1][t]+dc x[5][0][1][0][2][t]-ac x[0][0][1][0][0][t] x[5][1][0][0][0][t]-ac x[0][0][1][0][0][t] x[5][1][0][0][1][t]-ac x[0][0][1][0][0][t] x[5][1][0][0][2][t]+dc x[5][1][1][0][0][t]+dc x[5][1][1][0][1][t]+dc x[5][1][1][0][2][t]-ac x[0][0][1][0][0][t] x[5][2][0][0][0][t]-ac x[0][0][1][0][0][t] x[5][2][0][0][1][t]-ac x[0][0][1][0][0][t] x[5][2][0][0][2][t]+dc x[5][2][1][0][0][t]+dc x[5][2][1][0][1][t]+dc x[5][2][1][0][2][t]-ac x[0][0][1][0][0][t] x[6][0][0][0][0][t]-ac x[0][0][1][0][0][t] x[6][0][0][0][1][t]-ac x[0][0][1][0][0][t] x[6][0][0][0][2][t]+dc x[6][0][1][0][0][t]+up x[6][0][1][0][0][t]+dc x[6][0][1][0][1][t]+dc x[6][0][1][0][2][t]-ac x[0][0][1][0][0][t] x[6][1][0][0][0][t]-ac x[0][0][1][0][0][t] x[6][1][0][0][1][t]-ac x[0][0][1][0][0][t] x[6][1][0][0][2][t]+dc x[6][1][1][0][0][t]+dc x[6][1][1][0][1][t]+dc x[6][1][1][0][2][t]-ac x[0][0][1][0][0][t] x[6][2][0][0][0][t]-ac x[0][0][1][0][0][t] x[6][2][0][0][1][t]-ac x[0][0][1][0][0][t] x[6][2][0][0][2][t]+dc x[6][2][1][0][0][t]+dc x[6][2][1][0][1][t]+dc x[6][2][1][0][2][t]

dx[0][0][1][0][1][t]/dt=0

dx[0][0][1][0][2][t]/dt=0

dx[0][0][1][1][0][t]/dt=-ac Nf x[0][0][1][1][0][t] x[1][0][0][1][0][t]-ac Nf x[0][0][1][1][0][t] x[1][0][0][1][1][t]-ac Nf x[0][0][1][1][0][t] x[1][0][0][1][2][t]+dc x[1][0][1][1][0][t]+upu x[1][0][1][1][0][t]+dc x[1][0][1][1][1][t]+dc x[1][0][1][1][2][t]-ac Nf x[0][0][1][1][0][t] x[1][1][0][1][0][t]-ac Nf x[0][0][1][1][0][t] x[1][1][0][1][1][t]-ac Nf x[0][0][1][1][0][t] x[1][1][0][1][2][t]+dc x[1][1][1][1][0][t]+dc x[1][1][1][1][1][t]+dc x[1][1][1][1][2][t]-ac Nf x[0][0][1][1][0][t] x[1][2][0][1][0][t]-ac Nf x[0][0][1][1][0][t] x[1][2][0][1][1][t]-ac Nf x[0][0][1][1][0][t] x[1][2][0][1][2][t]+dc x[1][2][1][1][0][t]+dc x[1][2][1][1][1][t]+dc x[1][2][1][1][2][t]-ac Nf x[0][0][1][1][0][t] x[2][0][0][1][0][t]-ac Nf x[0][0][1][1][0][t] x[2][0][0][1][1][t]-ac Nf x[0][0][1][1][0][t] x[2][0][0][1][2][t]+dc x[2][0][1][1][0][t]+up x[2][0][1][1][0][t]+dc x[2][0][1][1][1][t]+dc x[2][0][1][1][2][t]-ac Nf x[0][0][1][1][0][t] x[2][1][0][1][0][t]-ac Nf x[0][0][1][1][0][t] x[2][1][0][1][1][t]-ac Nf x[0][0][1][1][0][t] x[2][1][0][1][2][t]+dc x[2][1][1][1][0][t]+dc x[2][1][1][1][1][t]+dc x[2][1][1][1][2][t]-ac Nf x[0][0][1][1][0][t] x[2][2][0][1][0][t]-ac Nf x[0][0][1][1][0][t] x[2][2][0][1][1][t]-ac Nf x[0][0][1][1][0][t] x[2][2][0][1][2][t]+dc x[2][2][1][1][0][t]+dc x[2][2][1][1][1][t]+dc x[2][2][1][1][2][t]-ac Nf x[0][0][1][1][0][t] x[3][0][0][1][0][t]-ac Nf x[0][0][1][1][0][t] x[3][0][0][1][1][t]-ac Nf x[0][0][1][1][0][t] x[3][0][0][1][2][t]+dc x[3][0][1][1][0][t]+upu x[3][0][1][1][0][t]+dc x[3][0][1][1][1][t]+dc x[3][0][1][1][2][t]-ac Nf x[0][0][1][1][0][t] x[3][1][0][1][0][t]-ac Nf x[0][0][1][1][0][t] x[3][1][0][1][1][t]-ac Nf x[0][0][1][1][0][t] x[3][1][0][1][2][t]+dc x[3][1][1][1][0][t]+dc x[3][1][1][1][1][t]+dc x[3][1][1][1][2][t]-ac Nf x[0][0][1][1][0][t] x[3][2][0][1][0][t]-ac Nf x[0][0][1][1][0][t] x[3][2][0][1][1][t]-ac Nf x[0][0][1][1][0][t] x[3][2][0][1][2][t]+dc x[3][2][1][1][0][t]+dc x[3][2][1][1][1][t]+dc x[3][2][1][1][2][t]-ac Nf x[0][0][1][1][0][t] x[4][0][0][1][0][t]-ac Nf x[0][0][1][1][0][t] x[4][0][0][1][1][t]-ac Nf x[0][0][1][1][0][t] x[4][0][0][1][2][t]+dc x[4][0][1][1][0][t]+up x[4][0][1][1][0][t]+dc x[4][0][1][1][1][t]+dc x[4][0][1][1][2][t]-ac Nf x[0][0][1][1][0][t] x[4][1][0][1][0][t]-ac Nf x[0][0][1][1][0][t] x[4][1][0][1][1][t]-ac Nf x[0][0][1][1][0][t] x[4][1][0][1][2][t]+dc x[4][1][1][1][0][t]+dc x[4][1][1][1][1][t]+dc x[4][1][1][1][2][t]-ac Nf x[0][0][1][1][0][t] x[4][2][0][1][0][t]-ac Nf x[0][0][1][1][0][t] x[4][2][0][1][1][t]-ac Nf x[0][0][1][1][0][t] x[4][2][0][1][2][t]+dc x[4][2][1][1][0][t]+dc x[4][2][1][1][1][t]+dc x[4][2][1][1][2][t]-ac Nf x[0][0][1][1][0][t] x[5][0][0][1][0][t]-ac Nf x[0][0][1][1][0][t] x[5][0][0][1][1][t]-ac Nf x[0][0][1][1][0][t] x[5][0][0][1][2][t]+dc x[5][0][1][1][0][t]+upu x[5][0][1][1][0][t]+dc x[5][0][1][1][1][t]+dc x[5][0][1][1][2][t]-ac Nf x[0][0][1][1][0][t] x[5][1][0][1][0][t]-ac Nf x[0][0][1][1][0][t] x[5][1][0][1][1][t]-ac Nf x[0][0][1][1][0][t] x[5][1][0][1][2][t]+dc x[5][1][1][1][0][t]+dc x[5][1][1][1][1][t]+dc x[5][1][1][1][2][t]-ac Nf x[0][0][1][1][0][t] x[5][2][0][1][0][t]-ac Nf x[0][0][1][1][0][t] x[5][2][0][1][1][t]-ac Nf x[0][0][1][1][0][t] x[5][2][0][1][2][t]+dc x[5][2][1][1][0][t]+dc x[5][2][1][1][1][t]+dc x[5][2][1][1][2][t]-ac Nf x[0][0][1][1][0][t] x[6][0][0][1][0][t]-ac Nf x[0][0][1][1][0][t] x[6][0][0][1][1][t]-ac Nf x[0][0][1][1][0][t] x[6][0][0][1][2][t]+dc x[6][0][1][1][0][t]+up x[6][0][1][1][0][t]+dc x[6][0][1][1][1][t]+dc x[6][0][1][1][2][t]-ac Nf x[0][0][1][1][0][t] x[6][1][0][1][0][t]-ac Nf x[0][0][1][1][0][t] x[6][1][0][1][1][t]-ac Nf x[0][0][1][1][0][t] x[6][1][0][1][2][t]+dc x[6][1][1][1][0][t]+dc x[6][1][1][1][1][t]+dc x[6][1][1][1][2][t]-ac Nf x[0][0][1][1][0][t] x[6][2][0][1][0][t]-ac Nf x[0][0][1][1][0][t] x[6][2][0][1][1][t]-ac Nf x[0][0][1][1][0][t] x[6][2][0][1][2][t]+dc x[6][2][1][1][0][t]+dc x[6][2][1][1][1][t]+dc x[6][2][1][1][2][t]

dx[0][0][1][1][1][t]/dt=0

dx[0][0][1][1][2][t]/dt=0

dx[0][1][0][0][0][t]/dt=tlr McRo[t]-uro x[0][1][0][0][0][t]-bbin x[0][0][0][0][1][t] x[0][1][0][0][0][t]-bbin x[0][0][0][0][2][t] x[0][1][0][0][0][t]+unbbin x[0][1][0][0][1][t]+unbbin x[0][1][0][0][2][t]-ar x[0][1][0][0][0][t] x[2][0][0][0][0][t]-ar x[0][1][0][0][0][t] x[2][0][1][0][0][t]+dr x[2][1][0][0][0][t]+dr x[2][1][1][0][0][t]-ar x[0][1][0][0][0][t] x[4][0][0][0][0][t]-ar x[0][1][0][0][0][t] x[4][0][1][0][0][t]+dr x[4][1][0][0][0][t]+dr x[4][1][1][0][0][t]-ar x[0][1][0][0][0][t] x[6][0][0][0][0][t]-ar x[0][1][0][0][0][t] x[6][0][1][0][0][t]+dr x[6][1][0][0][0][t]+dr x[6][1][1][0][0][t]

dx[0][1][0][0][1][t]/dt=bbin x[0][0][0][0][1][t] x[0][1][0][0][0][t]-unbbin x[0][1][0][0][1][t]-uro x[0][1][0][0][1][t]-ar x[0][1][0][0][1][t] x[2][0][0][0][0][t]-ar x[0][1][0][0][1][t] x[2][0][1][0][0][t]+dr x[2][1][0][0][1][t]+dr x[2][1][1][0][1][t]-ar x[0][1][0][0][1][t] x[4][0][0][0][0][t]-ar x[0][1][0][0][1][t] x[4][0][1][0][0][t]+dr x[4][1][0][0][1][t]+dr x[4][1][1][0][1][t]-ar x[0][1][0][0][1][t] x[6][0][0][0][0][t]-ar x[0][1][0][0][1][t] x[6][0][1][0][0][t]+dr x[6][1][0][0][1][t]+dr x[6][1][1][0][1][t]

dx[0][1][0][0][2][t]/dt=bbin x[0][0][0][0][2][t] x[0][1][0][0][0][t]-unbbin x[0][1][0][0][2][t]-uro x[0][1][0][0][2][t]-ar x[0][1][0][0][2][t] x[2][0][0][0][0][t]-ar x[0][1][0][0][2][t] x[2][0][1][0][0][t]+dr x[2][1][0][0][2][t]+dr x[2][1][1][0][2][t]-ar x[0][1][0][0][2][t] x[4][0][0][0][0][t]-ar x[0][1][0][0][2][t] x[4][0][1][0][0][t]+dr x[4][1][0][0][2][t]+dr x[4][1][1][0][2][t]-ar x[0][1][0][0][2][t] x[6][0][0][0][0][t]-ar x[0][1][0][0][2][t] x[6][0][1][0][0][t]+dr x[6][1][0][0][2][t]+dr x[6][1][1][0][2][t]

dx[0][1][0][1][0][t]/dt=-uro x[0][1][0][1][0][t]-bbin Nf x[0][0][0][1][1][t] x[0][1][0][1][0][t]-bbin Nf x[0][0][0][1][2][t] x[0][1][0][1][0][t]+unbbin x[0][1][0][1][1][t]+unbbin x[0][1][0][1][2][t]-ar Nf x[0][1][0][1][0][t] x[2][0][0][1][0][t]-ar Nf x[0][1][0][1][0][t] x[2][0][1][1][0][t]+dr x[2][1][0][1][0][t]+dr x[2][1][1][1][0][t]-ar Nf x[0][1][0][1][0][t] x[4][0][0][1][0][t]-ar Nf x[0][1][0][1][0][t] x[4][0][1][1][0][t]+dr x[4][1][0][1][0][t]+dr x[4][1][1][1][0][t]-ar Nf x[0][1][0][1][0][t] x[6][0][0][1][0][t]-ar Nf x[0][1][0][1][0][t] x[6][0][1][1][0][t]+dr x[6][1][0][1][0][t]+dr x[6][1][1][1][0][t]

dx[0][1][0][1][1][t]/dt=bbin Nf x[0][0][0][1][1][t] x[0][1][0][1][0][t]-unbbin x[0][1][0][1][1][t]-uro x[0][1][0][1][1][t]-ar Nf x[0][1][0][1][1][t] x[2][0][0][1][0][t]-ar Nf x[0][1][0][1][1][t] x[2][0][1][1][0][t]+dr x[2][1][0][1][1][t]+dr x[2][1][1][1][1][t]-ar Nf x[0][1][0][1][1][t] x[4][0][0][1][0][t]-ar Nf x[0][1][0][1][1][t] x[4][0][1][1][0][t]+dr x[4][1][0][1][1][t]+dr x[4][1][1][1][1][t]-ar Nf x[0][1][0][1][1][t] x[6][0][0][1][0][t]-ar Nf x[0][1][0][1][1][t] x[6][0][1][1][0][t]+dr x[6][1][0][1][1][t]+dr x[6][1][1][1][1][t]

dx[0][1][0][1][2][t]/dt=bbin Nf x[0][0][0][1][2][t] x[0][1][0][1][0][t]-unbbin x[0][1][0][1][2][t]-uro x[0][1][0][1][2][t]-ar Nf x[0][1][0][1][2][t] x[2][0][0][1][0][t]-ar Nf x[0][1][0][1][2][t] x[2][0][1][1][0][t]+dr x[2][1][0][1][2][t]+dr x[2][1][1][1][2][t]-ar Nf x[0][1][0][1][2][t] x[4][0][0][1][0][t]-ar Nf x[0][1][0][1][2][t] x[4][0][1][1][0][t]+dr x[4][1][0][1][2][t]+dr x[4][1][1][1][2][t]-ar Nf x[0][1][0][1][2][t] x[6][0][0][1][0][t]-ar Nf x[0][1][0][1][2][t] x[6][0][1][1][0][t]+dr x[6][1][0][1][2][t]+dr x[6][1][1][1][2][t]

dx[0][1][1][0][0][t]/dt=-bbin x[0][0][0][0][1][t] x[0][1][1][0][0][t]-bbin x[0][0][0][0][2][t] x[0][1][1][0][0][t]+unbbin x[0][1][1][0][1][t]+unbbin x[0][1][1][0][2][t]

dx[0][1][1][0][1][t]/dt=bbin x[0][0][0][0][1][t] x[0][1][1][0][0][t]-unbbin x[0][1][1][0][1][t]

dx[0][1][1][0][2][t]/dt=bbin x[0][0][0][0][2][t] x[0][1][1][0][0][t]-unbbin x[0][1][1][0][2][t]

dx[0][1][1][1][0][t]/dt=-bbin Nf x[0][0][0][1][1][t] x[0][1][1][1][0][t]-bbin Nf x[0][0][0][1][2][t] x[0][1][1][1][0][t]+unbbin x[0][1][1][1][1][t]+unbbin x[0][1][1][1][2][t]

dx[0][1][1][1][1][t]/dt=bbin Nf x[0][0][0][1][1][t] x[0][1][1][1][0][t]-unbbin x[0][1][1][1][1][t]

dx[0][1][1][1][2][t]/dt=bbin Nf x[0][0][0][1][2][t] x[0][1][1][1][0][t]-unbbin x[0][1][1][1][2][t]

dx[0][2][0][0][0][t]/dt=tlr McRt[t]-urt x[0][2][0][0][0][t]-bbin x[0][0][0][0][1][t] x[0][2][0][0][0][t]-bbin x[0][0][0][0][2][t] x[0][2][0][0][0][t]+unbbin x[0][2][0][0][1][t]+unbbin x[0][2][0][0][2][t]-ar x[0][2][0][0][0][t] x[2][0][0][0][0][t]-ar x[0][2][0][0][0][t] x[2][0][1][0][0][t]+dr x[2][2][0][0][0][t]+dr x[2][2][1][0][0][t]-ar x[0][2][0][0][0][t] x[4][0][0][0][0][t]-ar x[0][2][0][0][0][t] x[4][0][1][0][0][t]+dr x[4][2][0][0][0][t]+dr x[4][2][1][0][0][t]-ar x[0][2][0][0][0][t] x[6][0][0][0][0][t]-ar x[0][2][0][0][0][t] x[6][0][1][0][0][t]+dr x[6][2][0][0][0][t]+dr x[6][2][1][0][0][t]

dx[0][2][0][0][1][t]/dt=bbin x[0][0][0][0][1][t] x[0][2][0][0][0][t]-unbbin x[0][2][0][0][1][t]-urt x[0][2][0][0][1][t]-ar x[0][2][0][0][1][t] x[2][0][0][0][0][t]-ar x[0][2][0][0][1][t] x[2][0][1][0][0][t]+dr x[2][2][0][0][1][t]+dr x[2][2][1][0][1][t]-ar x[0][2][0][0][1][t] x[4][0][0][0][0][t]-ar x[0][2][0][0][1][t] x[4][0][1][0][0][t]+dr x[4][2][0][0][1][t]+dr x[4][2][1][0][1][t]-ar x[0][2][0][0][1][t] x[6][0][0][0][0][t]-ar x[0][2][0][0][1][t] x[6][0][1][0][0][t]+dr x[6][2][0][0][1][t]+dr x[6][2][1][0][1][t]

dx[0][2][0][0][2][t]/dt=bbin x[0][0][0][0][2][t] x[0][2][0][0][0][t]-unbbin x[0][2][0][0][2][t]-urt x[0][2][0][0][2][t]-ar x[0][2][0][0][2][t] x[2][0][0][0][0][t]-ar x[0][2][0][0][2][t] x[2][0][1][0][0][t]+dr x[2][2][0][0][2][t]+dr x[2][2][1][0][2][t]-ar x[0][2][0][0][2][t] x[4][0][0][0][0][t]-ar x[0][2][0][0][2][t] x[4][0][1][0][0][t]+dr x[4][2][0][0][2][t]+dr x[4][2][1][0][2][t]-ar x[0][2][0][0][2][t] x[6][0][0][0][0][t]-ar x[0][2][0][0][2][t] x[6][0][1][0][0][t]+dr x[6][2][0][0][2][t]+dr x[6][2][1][0][2][t]

dx[0][2][0][1][0][t]/dt=-urt x[0][2][0][1][0][t]-bbin Nf x[0][0][0][1][1][t] x[0][2][0][1][0][t]-bbin Nf x[0][0][0][1][2][t] x[0][2][0][1][0][t]+unbbin x[0][2][0][1][1][t]+unbbin x[0][2][0][1][2][t]-ar Nf x[0][2][0][1][0][t] x[2][0][0][1][0][t]-ar Nf x[0][2][0][1][0][t] x[2][0][1][1][0][t]+dr x[2][2][0][1][0][t]+dr x[2][2][1][1][0][t]-ar Nf x[0][2][0][1][0][t] x[4][0][0][1][0][t]-ar Nf x[0][2][0][1][0][t] x[4][0][1][1][0][t]+dr x[4][2][0][1][0][t]+dr x[4][2][1][1][0][t]-ar Nf x[0][2][0][1][0][t] x[6][0][0][1][0][t]-ar Nf x[0][2][0][1][0][t] x[6][0][1][1][0][t]+dr x[6][2][0][1][0][t]+dr x[6][2][1][1][0][t]

dx[0][2][0][1][1][t]/dt=bbin Nf x[0][0][0][1][1][t] x[0][2][0][1][0][t]-unbbin x[0][2][0][1][1][t]-urt x[0][2][0][1][1][t]-ar Nf x[0][2][0][1][1][t] x[2][0][0][1][0][t]-ar Nf x[0][2][0][1][1][t] x[2][0][1][1][0][t]+dr x[2][2][0][1][1][t]+dr x[2][2][1][1][1][t]-ar Nf x[0][2][0][1][1][t] x[4][0][0][1][0][t]-ar Nf x[0][2][0][1][1][t] x[4][0][1][1][0][t]+dr x[4][2][0][1][1][t]+dr x[4][2][1][1][1][t]-ar Nf x[0][2][0][1][1][t] x[6][0][0][1][0][t]-ar Nf x[0][2][0][1][1][t] x[6][0][1][1][0][t]+dr x[6][2][0][1][1][t]+dr x[6][2][1][1][1][t]

dx[0][2][0][1][2][t]/dt=bbin Nf x[0][0][0][1][2][t] x[0][2][0][1][0][t]-unbbin x[0][2][0][1][2][t]-urt x[0][2][0][1][2][t]-ar Nf x[0][2][0][1][2][t] x[2][0][0][1][0][t]-ar Nf x[0][2][0][1][2][t] x[2][0][1][1][0][t]+dr x[2][2][0][1][2][t]+dr x[2][2][1][1][2][t]-ar Nf x[0][2][0][1][2][t] x[4][0][0][1][0][t]-ar Nf x[0][2][0][1][2][t] x[4][0][1][1][0][t]+dr x[4][2][0][1][2][t]+dr x[4][2][1][1][2][t]-ar Nf x[0][2][0][1][2][t] x[6][0][0][1][0][t]-ar Nf x[0][2][0][1][2][t] x[6][0][1][1][0][t]+dr x[6][2][0][1][2][t]+dr x[6][2][1][1][2][t]

dx[0][2][1][0][0][t]/dt=-bbin x[0][0][0][0][1][t] x[0][2][1][0][0][t]-bbin x[0][0][0][0][2][t] x[0][2][1][0][0][t]+unbbin x[0][2][1][0][1][t]+unbbin x[0][2][1][0][2][t]

dx[0][2][1][0][1][t]/dt=bbin x[0][0][0][0][1][t] x[0][2][1][0][0][t]-unbbin x[0][2][1][0][1][t]

dx[0][2][1][0][2][t]/dt=bbin x[0][0][0][0][2][t] x[0][2][1][0][0][t]-unbbin x[0][2][1][0][2][t]

dx[0][2][1][1][0][t]/dt=-bbin Nf x[0][0][0][1][1][t] x[0][2][1][1][0][t]-bbin Nf x[0][0][0][1][2][t] x[0][2][1][1][0][t]+unbbin x[0][2][1][1][1][t]+unbbin x[0][2][1][1][2][t]

dx[0][2][1][1][1][t]/dt=bbin Nf x[0][0][0][1][1][t] x[0][2][1][1][0][t]-unbbin x[0][2][1][1][1][t]

dx[0][2][1][1][2][t]/dt=bbin Nf x[0][0][0][1][2][t] x[0][2][1][1][0][t]-unbbin x[0][2][1][1][2][t]

dx[1][0][0][0][0][t]/dt=tlp McPo[t]-upu x[1][0][0][0][0][t]-ac x[0][0][1][0][0][t] x[1][0][0][0][0][t]+dc x[1][0][1][0][0][t]

dx[1][0][0][0][1][t]/dt=-ac x[0][0][1][0][0][t] x[1][0][0][0][1][t]+dc x[1][0][1][0][1][t]

dx[1][0][0][0][2][t]/dt=-ac x[0][0][1][0][0][t] x[1][0][0][0][2][t]+dc x[1][0][1][0][2][t]

dx[1][0][0][1][0][t]/dt=-ac Nf x[0][0][1][1][0][t] x[1][0][0][1][0][t]+dc x[1][0][1][1][0][t]

dx[1][0][0][1][1][t]/dt=-ac Nf x[0][0][1][1][0][t] x[1][0][0][1][1][t]+dc x[1][0][1][1][1][t]

dx[1][0][0][1][2][t]/dt=-ac Nf x[0][0][1][1][0][t] x[1][0][0][1][2][t]+dc x[1][0][1][1][2][t]

dx[1][0][1][0][0][t]/dt=ac x[0][0][1][0][0][t] x[1][0][0][0][0][t]-dc x[1][0][1][0][0][t]-hoo x[1][0][1][0][0][t]-upu x[1][0][1][0][0][t]

dx[1][0][1][0][1][t]/dt=ac x[0][0][1][0][0][t] x[1][0][0][0][1][t]-dc x[1][0][1][0][1][t]

dx[1][0][1][0][2][t]/dt=ac x[0][0][1][0][0][t] x[1][0][0][0][2][t]-dc x[1][0][1][0][2][t]

dx[1][0][1][1][0][t]/dt=ac Nf x[0][0][1][1][0][t] x[1][0][0][1][0][t]-dc x[1][0][1][1][0][t]

dx[1][0][1][1][1][t]/dt=ac Nf x[0][0][1][1][0][t] x[1][0][0][1][1][t]-dc x[1][0][1][1][1][t]

dx[1][0][1][1][2][t]/dt=ac Nf x[0][0][1][1][0][t] x[1][0][0][1][2][t]-dc x[1][0][1][1][2][t]

dx[1][1][0][0][0][t]/dt=-bbin x[0][0][0][0][1][t] x[1][1][0][0][0][t]-bbin x[0][0][0][0][2][t] x[1][1][0][0][0][t]-ac x[0][0][1][0][0][t] x[1][1][0][0][0][t]+unbbin x[1][1][0][0][1][t]+unbbin x[1][1][0][0][2][t]+dc x[1][1][1][0][0][t]

dx[1][1][0][0][1][t]/dt=bbin x[0][0][0][0][1][t] x[1][1][0][0][0][t]-unbbin x[1][1][0][0][1][t]-ac x[0][0][1][0][0][t] x[1][1][0][0][1][t]+dc x[1][1][1][0][1][t]

dx[1][1][0][0][2][t]/dt=bbin x[0][0][0][0][2][t] x[1][1][0][0][0][t]-unbbin x[1][1][0][0][2][t]-ac x[0][0][1][0][0][t] x[1][1][0][0][2][t]+dc x[1][1][1][0][2][t]

dx[1][1][0][1][0][t]/dt=-bbin Nf x[0][0][0][1][1][t] x[1][1][0][1][0][t]-bbin Nf x[0][0][0][1][2][t] x[1][1][0][1][0][t]-ac Nf x[0][0][1][1][0][t] x[1][1][0][1][0][t]+unbbin x[1][1][0][1][1][t]+unbbin x[1][1][0][1][2][t]+dc x[1][1][1][1][0][t]

dx[1][1][0][1][1][t]/dt=bbin Nf x[0][0][0][1][1][t] x[1][1][0][1][0][t]-unbbin x[1][1][0][1][1][t]-ac Nf x[0][0][1][1][0][t] x[1][1][0][1][1][t]+dc x[1][1][1][1][1][t]

dx[1][1][0][1][2][t]/dt=bbin Nf x[0][0][0][1][2][t] x[1][1][0][1][0][t]-unbbin x[1][1][0][1][2][t]-ac Nf x[0][0][1][1][0][t] x[1][1][0][1][2][t]+dc x[1][1][1][1][2][t]

dx[1][1][1][0][0][t]/dt=ac x[0][0][1][0][0][t] x[1][1][0][0][0][t]-dc x[1][1][1][0][0][t]-bbin x[0][0][0][0][1][t] x[1][1][1][0][0][t]-bbin x[0][0][0][0][2][t] x[1][1][1][0][0][t]+unbbin x[1][1][1][0][1][t]+unbbin x[1][1][1][0][2][t]

dx[1][1][1][0][1][t]/dt=ac x[0][0][1][0][0][t] x[1][1][0][0][1][t]+bbin x[0][0][0][0][1][t] x[1][1][1][0][0][t]-dc x[1][1][1][0][1][t]-unbbin x[1][1][1][0][1][t]

dx[1][1][1][0][2][t]/dt=ac x[0][0][1][0][0][t] x[1][1][0][0][2][t]+bbin x[0][0][0][0][2][t] x[1][1][1][0][0][t]-dc x[1][1][1][0][2][t]-unbbin x[1][1][1][0][2][t]

dx[1][1][1][1][0][t]/dt=ac Nf x[0][0][1][1][0][t] x[1][1][0][1][0][t]-dc x[1][1][1][1][0][t]-bbin Nf x[0][0][0][1][1][t] x[1][1][1][1][0][t]-bbin Nf x[0][0][0][1][2][t] x[1][1][1][1][0][t]+unbbin x[1][1][1][1][1][t]+unbbin x[1][1][1][1][2][t]

dx[1][1][1][1][1][t]/dt=ac Nf x[0][0][1][1][0][t] x[1][1][0][1][1][t]+bbin Nf x[0][0][0][1][1][t] x[1][1][1][1][0][t]-dc x[1][1][1][1][1][t]-unbbin x[1][1][1][1][1][t]

dx[1][1][1][1][2][t]/dt=ac Nf x[0][0][1][1][0][t] x[1][1][0][1][2][t]+bbin Nf x[0][0][0][1][2][t] x[1][1][1][1][0][t]-dc x[1][1][1][1][2][t]-unbbin x[1][1][1][1][2][t]

dx[1][2][0][0][0][t]/dt=-bbin x[0][0][0][0][1][t] x[1][2][0][0][0][t]-bbin x[0][0][0][0][2][t] x[1][2][0][0][0][t]-ac x[0][0][1][0][0][t] x[1][2][0][0][0][t]+unbbin x[1][2][0][0][1][t]+unbbin x[1][2][0][0][2][t]+dc x[1][2][1][0][0][t]

dx[1][2][0][0][1][t]/dt=bbin x[0][0][0][0][1][t] x[1][2][0][0][0][t]-unbbin x[1][2][0][0][1][t]-ac x[0][0][1][0][0][t] x[1][2][0][0][1][t]+dc x[1][2][1][0][1][t]

dx[1][2][0][0][2][t]/dt=bbin x[0][0][0][0][2][t] x[1][2][0][0][0][t]-unbbin x[1][2][0][0][2][t]-ac x[0][0][1][0][0][t] x[1][2][0][0][2][t]+dc x[1][2][1][0][2][t]

dx[1][2][0][1][0][t]/dt=-bbin Nf x[0][0][0][1][1][t] x[1][2][0][1][0][t]-bbin Nf x[0][0][0][1][2][t] x[1][2][0][1][0][t]-ac Nf x[0][0][1][1][0][t] x[1][2][0][1][0][t]+unbbin x[1][2][0][1][1][t]+unbbin x[1][2][0][1][2][t]+dc x[1][2][1][1][0][t]

dx[1][2][0][1][1][t]/dt=bbin Nf x[0][0][0][1][1][t] x[1][2][0][1][0][t]-unbbin x[1][2][0][1][1][t]-ac Nf x[0][0][1][1][0][t] x[1][2][0][1][1][t]+dc x[1][2][1][1][1][t]

dx[1][2][0][1][2][t]/dt=bbin Nf x[0][0][0][1][2][t] x[1][2][0][1][0][t]-unbbin x[1][2][0][1][2][t]-ac Nf x[0][0][1][1][0][t] x[1][2][0][1][2][t]+dc x[1][2][1][1][2][t]

dx[1][2][1][0][0][t]/dt=ac x[0][0][1][0][0][t] x[1][2][0][0][0][t]-dc x[1][2][1][0][0][t]-bbin x[0][0][0][0][1][t] x[1][2][1][0][0][t]-bbin x[0][0][0][0][2][t] x[1][2][1][0][0][t]+unbbin x[1][2][1][0][1][t]+unbbin x[1][2][1][0][2][t]

dx[1][2][1][0][1][t]/dt=ac x[0][0][1][0][0][t] x[1][2][0][0][1][t]+bbin x[0][0][0][0][1][t] x[1][2][1][0][0][t]-dc x[1][2][1][0][1][t]-unbbin x[1][2][1][0][1][t]

dx[1][2][1][0][2][t]/dt=ac x[0][0][1][0][0][t] x[1][2][0][0][2][t]+bbin x[0][0][0][0][2][t] x[1][2][1][0][0][t]-dc x[1][2][1][0][2][t]-unbbin x[1][2][1][0][2][t]

dx[1][2][1][1][0][t]/dt=ac Nf x[0][0][1][1][0][t] x[1][2][0][1][0][t]-dc x[1][2][1][1][0][t]-bbin Nf x[0][0][0][1][1][t] x[1][2][1][1][0][t]-bbin Nf x[0][0][0][1][2][t] x[1][2][1][1][0][t]+unbbin x[1][2][1][1][1][t]+unbbin x[1][2][1][1][2][t]

dx[1][2][1][1][1][t]/dt=ac Nf x[0][0][1][1][0][t] x[1][2][0][1][1][t]+bbin Nf x[0][0][0][1][1][t] x[1][2][1][1][0][t]-dc x[1][2][1][1][1][t]-unbbin x[1][2][1][1][1][t]

dx[1][2][1][1][2][t]/dt=ac Nf x[0][0][1][1][0][t] x[1][2][0][1][2][t]+bbin Nf x[0][0][0][1][2][t] x[1][2][1][1][0][t]-dc x[1][2][1][1][2][t]-unbbin x[1][2][1][1][2][t]

dx[2][0][0][0][0][t]/dt=-nl x[2][0][0][0][0][t]-up x[2][0][0][0][0][t]-ac x[0][0][1][0][0][t] x[2][0][0][0][0][t]-ar x[0][1][0][0][0][t] x[2][0][0][0][0][t]-ar x[0][1][0][0][1][t] x[2][0][0][0][0][t]-ar x[0][1][0][0][2][t] x[2][0][0][0][0][t]-ar x[0][2][0][0][0][t] x[2][0][0][0][0][t]-ar x[0][2][0][0][1][t] x[2][0][0][0][0][t]-ar x[0][2][0][0][2][t] x[2][0][0][0][0][t]+ne x[2][0][0][1][0][t]+dc x[2][0][1][0][0][t]+dr x[2][1][0][0][0][t]+dr x[2][1][0][0][1][t]+dr x[2][1][0][0][2][t]+dr x[2][2][0][0][0][t]+dr x[2][2][0][0][1][t]+dr x[2][2][0][0][2][t]

dx[2][0][0][0][1][t]/dt=-nl x[2][0][0][0][1][t]-ac x[0][0][1][0][0][t] x[2][0][0][0][1][t]+dc x[2][0][1][0][1][t]

dx[2][0][0][0][2][t]/dt=-nl x[2][0][0][0][2][t]-ac x[0][0][1][0][0][t] x[2][0][0][0][2][t]+dc x[2][0][1][0][2][t]

dx[2][0][0][1][0][t]/dt=nl x[2][0][0][0][0][t]-ne x[2][0][0][1][0][t]-up x[2][0][0][1][0][t]-ac Nf x[0][0][1][1][0][t] x[2][0][0][1][0][t]-ar Nf x[0][1][0][1][0][t] x[2][0][0][1][0][t]-ar Nf x[0][1][0][1][1][t] x[2][0][0][1][0][t]-ar Nf x[0][1][0][1][2][t] x[2][0][0][1][0][t]-ar Nf x[0][2][0][1][0][t] x[2][0][0][1][0][t]-ar Nf x[0][2][0][1][1][t] x[2][0][0][1][0][t]-ar Nf x[0][2][0][1][2][t] x[2][0][0][1][0][t]+dc x[2][0][1][1][0][t]+dr x[2][1][0][1][0][t]+dr x[2][1][0][1][1][t]+dr x[2][1][0][1][2][t]+dr x[2][2][0][1][0][t]+dr x[2][2][0][1][1][t]+dr x[2][2][0][1][2][t]

dx[2][0][0][1][1][t]/dt=nl x[2][0][0][0][1][t]-ac Nf x[0][0][1][1][0][t] x[2][0][0][1][1][t]+dc x[2][0][1][1][1][t]

dx[2][0][0][1][2][t]/dt=nl x[2][0][0][0][2][t]-ac Nf x[0][0][1][1][0][t] x[2][0][0][1][2][t]+dc x[2][0][1][1][2][t]

dx[2][0][1][0][0][t]/dt=hoo x[1][0][1][0][0][t]+ac x[0][0][1][0][0][t] x[2][0][0][0][0][t]-dc x[2][0][1][0][0][t]-hot x[2][0][1][0][0][t]-nl x[2][0][1][0][0][t]-up x[2][0][1][0][0][t]-ar x[0][1][0][0][0][t] x[2][0][1][0][0][t]-ar x[0][1][0][0][1][t] x[2][0][1][0][0][t]-ar x[0][1][0][0][2][t] x[2][0][1][0][0][t]-ar x[0][2][0][0][0][t] x[2][0][1][0][0][t]-ar x[0][2][0][0][1][t] x[2][0][1][0][0][t]-ar x[0][2][0][0][2][t] x[2][0][1][0][0][t]+ne x[2][0][1][1][0][t]+dr x[2][1][1][0][0][t]+dr x[2][1][1][0][1][t]+dr x[2][1][1][0][2][t]+dr x[2][2][1][0][0][t]+dr x[2][2][1][0][1][t]+dr x[2][2][1][0][2][t]

dx[2][0][1][0][1][t]/dt=ac x[0][0][1][0][0][t] x[2][0][0][0][1][t]-dc x[2][0][1][0][1][t]-hot x[2][0][1][0][1][t]-nl x[2][0][1][0][1][t]

dx[2][0][1][0][2][t]/dt=ac x[0][0][1][0][0][t] x[2][0][0][0][2][t]-dc x[2][0][1][0][2][t]-hot x[2][0][1][0][2][t]-nl x[2][0][1][0][2][t]

dx[2][0][1][1][0][t]/dt=ac Nf x[0][0][1][1][0][t] x[2][0][0][1][0][t]+nl x[2][0][1][0][0][t]-dc x[2][0][1][1][0][t]-hot x[2][0][1][1][0][t]-ne x[2][0][1][1][0][t]-up x[2][0][1][1][0][t]-ar Nf x[0][1][0][1][0][t] x[2][0][1][1][0][t]-ar Nf x[0][1][0][1][1][t] x[2][0][1][1][0][t]-ar Nf x[0][1][0][1][2][t] x[2][0][1][1][0][t]-ar Nf x[0][2][0][1][0][t] x[2][0][1][1][0][t]-ar Nf x[0][2][0][1][1][t] x[2][0][1][1][0][t]-ar Nf x[0][2][0][1][2][t] x[2][0][1][1][0][t]+dr x[2][1][1][1][0][t]+dr x[2][1][1][1][1][t]+dr x[2][1][1][1][2][t]+dr x[2][2][1][1][0][t]+dr x[2][2][1][1][1][t]+dr x[2][2][1][1][2][t]

dx[2][0][1][1][1][t]/dt=ac Nf x[0][0][1][1][0][t] x[2][0][0][1][1][t]+nl x[2][0][1][0][1][t]-dc x[2][0][1][1][1][t]-hot x[2][0][1][1][1][t]

dx[2][0][1][1][2][t]/dt=ac Nf x[0][0][1][1][0][t] x[2][0][0][1][2][t]+nl x[2][0][1][0][2][t]-dc x[2][0][1][1][2][t]-hot x[2][0][1][1][2][t]

dx[2][1][0][0][0][t]/dt=ar x[0][1][0][0][0][t] x[2][0][0][0][0][t]-dr x[2][1][0][0][0][t]-nl x[2][1][0][0][0][t]-bbin x[0][0][0][0][1][t] x[2][1][0][0][0][t]-bbin x[0][0][0][0][2][t] x[2][1][0][0][0][t]-ac x[0][0][1][0][0][t] x[2][1][0][0][0][t]+unbbin x[2][1][0][0][1][t]+unbbin x[2][1][0][0][2][t]+ne x[2][1][0][1][0][t]+dc x[2][1][1][0][0][t]

dx[2][1][0][0][1][t]/dt=ar x[0][1][0][0][1][t] x[2][0][0][0][0][t]+bbin x[0][0][0][0][1][t] x[2][1][0][0][0][t]-dr x[2][1][0][0][1][t]-nl x[2][1][0][0][1][t]-unbbin x[2][1][0][0][1][t]-ac x[0][0][1][0][0][t] x[2][1][0][0][1][t]+dc x[2][1][1][0][1][t]

dx[2][1][0][0][2][t]/dt=ar x[0][1][0][0][2][t] x[2][0][0][0][0][t]+bbin x[0][0][0][0][2][t] x[2][1][0][0][0][t]-dr x[2][1][0][0][2][t]-nl x[2][1][0][0][2][t]-unbbin x[2][1][0][0][2][t]-ac x[0][0][1][0][0][t] x[2][1][0][0][2][t]+dc x[2][1][1][0][2][t]

dx[2][1][0][1][0][t]/dt=ar Nf x[0][1][0][1][0][t] x[2][0][0][1][0][t]+nl x[2][1][0][0][0][t]-dr x[2][1][0][1][0][t]-ne x[2][1][0][1][0][t]-bbin Nf x[0][0][0][1][1][t] x[2][1][0][1][0][t]-bbin Nf x[0][0][0][1][2][t] x[2][1][0][1][0][t]-ac Nf x[0][0][1][1][0][t] x[2][1][0][1][0][t]+unbbin x[2][1][0][1][1][t]+unbbin x[2][1][0][1][2][t]+dc x[2][1][1][1][0][t]

dx[2][1][0][1][1][t]/dt=ar Nf x[0][1][0][1][1][t] x[2][0][0][1][0][t]+nl x[2][1][0][0][1][t]+bbin Nf x[0][0][0][1][1][t] x[2][1][0][1][0][t]-dr x[2][1][0][1][1][t]-unbbin x[2][1][0][1][1][t]-ac Nf x[0][0][1][1][0][t] x[2][1][0][1][1][t]+dc x[2][1][1][1][1][t]

dx[2][1][0][1][2][t]/dt=ar Nf x[0][1][0][1][2][t] x[2][0][0][1][0][t]+nl x[2][1][0][0][2][t]+bbin Nf x[0][0][0][1][2][t] x[2][1][0][1][0][t]-dr x[2][1][0][1][2][t]-unbbin x[2][1][0][1][2][t]-ac Nf x[0][0][1][1][0][t] x[2][1][0][1][2][t]+dc x[2][1][1][1][2][t]

dx[2][1][1][0][0][t]/dt=ar x[0][1][0][0][0][t] x[2][0][1][0][0][t]+ac x[0][0][1][0][0][t] x[2][1][0][0][0][t]-dc x[2][1][1][0][0][t]-dr x[2][1][1][0][0][t]-hot x[2][1][1][0][0][t]-nl x[2][1][1][0][0][t]-bbin x[0][0][0][0][1][t] x[2][1][1][0][0][t]-bbin x[0][0][0][0][2][t] x[2][1][1][0][0][t]+unbbin x[2][1][1][0][1][t]+unbbin x[2][1][1][0][2][t]+ne x[2][1][1][1][0][t]

dx[2][1][1][0][1][t]/dt=ar x[0][1][0][0][1][t] x[2][0][1][0][0][t]+ac x[0][0][1][0][0][t] x[2][1][0][0][1][t]+bbin x[0][0][0][0][1][t] x[2][1][1][0][0][t]-dc x[2][1][1][0][1][t]-dr x[2][1][1][0][1][t]-hot x[2][1][1][0][1][t]-nl x[2][1][1][0][1][t]-unbbin x[2][1][1][0][1][t]

dx[2][1][1][0][2][t]/dt=ar x[0][1][0][0][2][t] x[2][0][1][0][0][t]+ac x[0][0][1][0][0][t] x[2][1][0][0][2][t]+bbin x[0][0][0][0][2][t] x[2][1][1][0][0][t]-dc x[2][1][1][0][2][t]-dr x[2][1][1][0][2][t]-hot x[2][1][1][0][2][t]-nl x[2][1][1][0][2][t]-unbbin x[2][1][1][0][2][t]

dx[2][1][1][1][0][t]/dt=ar Nf x[0][1][0][1][0][t] x[2][0][1][1][0][t]+ac Nf x[0][0][1][1][0][t] x[2][1][0][1][0][t]+nl x[2][1][1][0][0][t]-dc x[2][1][1][1][0][t]-dr x[2][1][1][1][0][t]-hot x[2][1][1][1][0][t]-ne x[2][1][1][1][0][t]-bbin Nf x[0][0][0][1][1][t] x[2][1][1][1][0][t]-bbin Nf x[0][0][0][1][2][t] x[2][1][1][1][0][t]+unbbin x[2][1][1][1][1][t]+unbbin x[2][1][1][1][2][t]

dx[2][1][1][1][1][t]/dt=ar Nf x[0][1][0][1][1][t] x[2][0][1][1][0][t]+ac Nf x[0][0][1][1][0][t] x[2][1][0][1][1][t]+nl x[2][1][1][0][1][t]+bbin Nf x[0][0][0][1][1][t] x[2][1][1][1][0][t]-dc x[2][1][1][1][1][t]-dr x[2][1][1][1][1][t]-hot x[2][1][1][1][1][t]-unbbin x[2][1][1][1][1][t]

dx[2][1][1][1][2][t]/dt=ar Nf x[0][1][0][1][2][t] x[2][0][1][1][0][t]+ac Nf x[0][0][1][1][0][t] x[2][1][0][1][2][t]+nl x[2][1][1][0][2][t]+bbin Nf x[0][0][0][1][2][t] x[2][1][1][1][0][t]-dc x[2][1][1][1][2][t]-dr x[2][1][1][1][2][t]-hot x[2][1][1][1][2][t]-unbbin x[2][1][1][1][2][t]

dx[2][2][0][0][0][t]/dt=ar x[0][2][0][0][0][t] x[2][0][0][0][0][t]-dr x[2][2][0][0][0][t]-nl x[2][2][0][0][0][t]-bbin x[0][0][0][0][1][t] x[2][2][0][0][0][t]-bbin x[0][0][0][0][2][t] x[2][2][0][0][0][t]-ac x[0][0][1][0][0][t] x[2][2][0][0][0][t]+unbbin x[2][2][0][0][1][t]+unbbin x[2][2][0][0][2][t]+ne x[2][2][0][1][0][t]+dc x[2][2][1][0][0][t]

dx[2][2][0][0][1][t]/dt=ar x[0][2][0][0][1][t] x[2][0][0][0][0][t]+bbin x[0][0][0][0][1][t] x[2][2][0][0][0][t]-dr x[2][2][0][0][1][t]-nl x[2][2][0][0][1][t]-unbbin x[2][2][0][0][1][t]-ac x[0][0][1][0][0][t] x[2][2][0][0][1][t]+dc x[2][2][1][0][1][t]

dx[2][2][0][0][2][t]/dt=ar x[0][2][0][0][2][t] x[2][0][0][0][0][t]+bbin x[0][0][0][0][2][t] x[2][2][0][0][0][t]-dr x[2][2][0][0][2][t]-nl x[2][2][0][0][2][t]-unbbin x[2][2][0][0][2][t]-ac x[0][0][1][0][0][t] x[2][2][0][0][2][t]+dc x[2][2][1][0][2][t]

dx[2][2][0][1][0][t]/dt=ar Nf x[0][2][0][1][0][t] x[2][0][0][1][0][t]+nl x[2][2][0][0][0][t]-dr x[2][2][0][1][0][t]-ne x[2][2][0][1][0][t]-bbin Nf x[0][0][0][1][1][t] x[2][2][0][1][0][t]-bbin Nf x[0][0][0][1][2][t] x[2][2][0][1][0][t]-ac Nf x[0][0][1][1][0][t] x[2][2][0][1][0][t]+unbbin x[2][2][0][1][1][t]+unbbin x[2][2][0][1][2][t]+dc x[2][2][1][1][0][t]

dx[2][2][0][1][1][t]/dt=ar Nf x[0][2][0][1][1][t] x[2][0][0][1][0][t]+nl x[2][2][0][0][1][t]+bbin Nf x[0][0][0][1][1][t] x[2][2][0][1][0][t]-dr x[2][2][0][1][1][t]-unbbin x[2][2][0][1][1][t]-ac Nf x[0][0][1][1][0][t] x[2][2][0][1][1][t]+dc x[2][2][1][1][1][t]

dx[2][2][0][1][2][t]/dt=ar Nf x[0][2][0][1][2][t] x[2][0][0][1][0][t]+nl x[2][2][0][0][2][t]+bbin Nf x[0][0][0][1][2][t] x[2][2][0][1][0][t]-dr x[2][2][0][1][2][t]-unbbin x[2][2][0][1][2][t]-ac Nf x[0][0][1][1][0][t] x[2][2][0][1][2][t]+dc x[2][2][1][1][2][t]

dx[2][2][1][0][0][t]/dt=ar x[0][2][0][0][0][t] x[2][0][1][0][0][t]+ac x[0][0][1][0][0][t] x[2][2][0][0][0][t]-dc x[2][2][1][0][0][t]-dr x[2][2][1][0][0][t]-hot x[2][2][1][0][0][t]-nl x[2][2][1][0][0][t]-bbin x[0][0][0][0][1][t] x[2][2][1][0][0][t]-bbin x[0][0][0][0][2][t] x[2][2][1][0][0][t]+unbbin x[2][2][1][0][1][t]+unbbin x[2][2][1][0][2][t]+ne x[2][2][1][1][0][t]

dx[2][2][1][0][1][t]/dt=ar x[0][2][0][0][1][t] x[2][0][1][0][0][t]+ac x[0][0][1][0][0][t] x[2][2][0][0][1][t]+bbin x[0][0][0][0][1][t] x[2][2][1][0][0][t]-dc x[2][2][1][0][1][t]-dr x[2][2][1][0][1][t]-hot x[2][2][1][0][1][t]-nl x[2][2][1][0][1][t]-unbbin x[2][2][1][0][1][t]

dx[2][2][1][0][2][t]/dt=ar x[0][2][0][0][2][t] x[2][0][1][0][0][t]+ac x[0][0][1][0][0][t] x[2][2][0][0][2][t]+bbin x[0][0][0][0][2][t] x[2][2][1][0][0][t]-dc x[2][2][1][0][2][t]-dr x[2][2][1][0][2][t]-hot x[2][2][1][0][2][t]-nl x[2][2][1][0][2][t]-unbbin x[2][2][1][0][2][t]

dx[2][2][1][1][0][t]/dt=ar Nf x[0][2][0][1][0][t] x[2][0][1][1][0][t]+ac Nf x[0][0][1][1][0][t] x[2][2][0][1][0][t]+nl x[2][2][1][0][0][t]-dc x[2][2][1][1][0][t]-dr x[2][2][1][1][0][t]-hot x[2][2][1][1][0][t]-ne x[2][2][1][1][0][t]-bbin Nf x[0][0][0][1][1][t] x[2][2][1][1][0][t]-bbin Nf x[0][0][0][1][2][t] x[2][2][1][1][0][t]+unbbin x[2][2][1][1][1][t]+unbbin x[2][2][1][1][2][t]

dx[2][2][1][1][1][t]/dt=ar Nf x[0][2][0][1][1][t] x[2][0][1][1][0][t]+ac Nf x[0][0][1][1][0][t] x[2][2][0][1][1][t]+nl x[2][2][1][0][1][t]+bbin Nf x[0][0][0][1][1][t] x[2][2][1][1][0][t]-dc x[2][2][1][1][1][t]-dr x[2][2][1][1][1][t]-hot x[2][2][1][1][1][t]-unbbin x[2][2][1][1][1][t]

dx[2][2][1][1][2][t]/dt=ar Nf x[0][2][0][1][2][t] x[2][0][1][1][0][t]+ac Nf x[0][0][1][1][0][t] x[2][2][0][1][2][t]+nl x[2][2][1][0][2][t]+bbin Nf x[0][0][0][1][2][t] x[2][2][1][1][0][t]-dc x[2][2][1][1][2][t]-dr x[2][2][1][1][2][t]-hot x[2][2][1][1][2][t]-unbbin x[2][2][1][1][2][t]

dx[3][0][0][0][0][t]/dt=-upu x[3][0][0][0][0][t]-ac x[0][0][1][0][0][t] x[3][0][0][0][0][t]+dc x[3][0][1][0][0][t]

dx[3][0][0][0][1][t]/dt=-ac x[0][0][1][0][0][t] x[3][0][0][0][1][t]+dc x[3][0][1][0][1][t]

dx[3][0][0][0][2][t]/dt=-ac x[0][0][1][0][0][t] x[3][0][0][0][2][t]+dc x[3][0][1][0][2][t]

dx[3][0][0][1][0][t]/dt=-ac Nf x[0][0][1][1][0][t] x[3][0][0][1][0][t]+dc x[3][0][1][1][0][t]

dx[3][0][0][1][1][t]/dt=-ac Nf x[0][0][1][1][0][t] x[3][0][0][1][1][t]+dc x[3][0][1][1][1][t]

dx[3][0][0][1][2][t]/dt=-ac Nf x[0][0][1][1][0][t] x[3][0][0][1][2][t]+dc x[3][0][1][1][2][t]

dx[3][0][1][0][0][t]/dt=ac x[0][0][1][0][0][t] x[3][0][0][0][0][t]-dc x[3][0][1][0][0][t]-hoo x[3][0][1][0][0][t]-upu x[3][0][1][0][0][t]

dx[3][0][1][0][1][t]/dt=ac x[0][0][1][0][0][t] x[3][0][0][0][1][t]-dc x[3][0][1][0][1][t]

dx[3][0][1][0][2][t]/dt=ac x[0][0][1][0][0][t] x[3][0][0][0][2][t]-dc x[3][0][1][0][2][t]

dx[3][0][1][1][0][t]/dt=ac Nf x[0][0][1][1][0][t] x[3][0][0][1][0][t]-dc x[3][0][1][1][0][t]

dx[3][0][1][1][1][t]/dt=ac Nf x[0][0][1][1][0][t] x[3][0][0][1][1][t]-dc x[3][0][1][1][1][t]

dx[3][0][1][1][2][t]/dt=ac Nf x[0][0][1][1][0][t] x[3][0][0][1][2][t]-dc x[3][0][1][1][2][t]

dx[3][1][0][0][0][t]/dt=-bbin x[0][0][0][0][1][t] x[3][1][0][0][0][t]-bbin x[0][0][0][0][2][t] x[3][1][0][0][0][t]-ac x[0][0][1][0][0][t] x[3][1][0][0][0][t]+unbbin x[3][1][0][0][1][t]+unbbin x[3][1][0][0][2][t]+dc x[3][1][1][0][0][t]

dx[3][1][0][0][1][t]/dt=bbin x[0][0][0][0][1][t] x[3][1][0][0][0][t]-unbbin x[3][1][0][0][1][t]-ac x[0][0][1][0][0][t] x[3][1][0][0][1][t]+dc x[3][1][1][0][1][t]

dx[3][1][0][0][2][t]/dt=bbin x[0][0][0][0][2][t] x[3][1][0][0][0][t]-unbbin x[3][1][0][0][2][t]-ac x[0][0][1][0][0][t] x[3][1][0][0][2][t]+dc x[3][1][1][0][2][t]

dx[3][1][0][1][0][t]/dt=-bbin Nf x[0][0][0][1][1][t] x[3][1][0][1][0][t]-bbin Nf x[0][0][0][1][2][t] x[3][1][0][1][0][t]-ac Nf x[0][0][1][1][0][t] x[3][1][0][1][0][t]+unbbin x[3][1][0][1][1][t]+unbbin x[3][1][0][1][2][t]+dc x[3][1][1][1][0][t]

dx[3][1][0][1][1][t]/dt=bbin Nf x[0][0][0][1][1][t] x[3][1][0][1][0][t]-unbbin x[3][1][0][1][1][t]-ac Nf x[0][0][1][1][0][t] x[3][1][0][1][1][t]+dc x[3][1][1][1][1][t]

dx[3][1][0][1][2][t]/dt=bbin Nf x[0][0][0][1][2][t] x[3][1][0][1][0][t]-unbbin x[3][1][0][1][2][t]-ac Nf x[0][0][1][1][0][t] x[3][1][0][1][2][t]+dc x[3][1][1][1][2][t]

dx[3][1][1][0][0][t]/dt=ac x[0][0][1][0][0][t] x[3][1][0][0][0][t]-dc x[3][1][1][0][0][t]-bbin x[0][0][0][0][1][t] x[3][1][1][0][0][t]-bbin x[0][0][0][0][2][t] x[3][1][1][0][0][t]+unbbin x[3][1][1][0][1][t]+unbbin x[3][1][1][0][2][t]

dx[3][1][1][0][1][t]/dt=ac x[0][0][1][0][0][t] x[3][1][0][0][1][t]+bbin x[0][0][0][0][1][t] x[3][1][1][0][0][t]-dc x[3][1][1][0][1][t]-unbbin x[3][1][1][0][1][t]

dx[3][1][1][0][2][t]/dt=ac x[0][0][1][0][0][t] x[3][1][0][0][2][t]+bbin x[0][0][0][0][2][t] x[3][1][1][0][0][t]-dc x[3][1][1][0][2][t]-unbbin x[3][1][1][0][2][t]

dx[3][1][1][1][0][t]/dt=ac Nf x[0][0][1][1][0][t] x[3][1][0][1][0][t]-dc x[3][1][1][1][0][t]-bbin Nf x[0][0][0][1][1][t] x[3][1][1][1][0][t]-bbin Nf x[0][0][0][1][2][t] x[3][1][1][1][0][t]+unbbin x[3][1][1][1][1][t]+unbbin x[3][1][1][1][2][t]

dx[3][1][1][1][1][t]/dt=ac Nf x[0][0][1][1][0][t] x[3][1][0][1][1][t]+bbin Nf x[0][0][0][1][1][t] x[3][1][1][1][0][t]-dc x[3][1][1][1][1][t]-unbbin x[3][1][1][1][1][t]

dx[3][1][1][1][2][t]/dt=ac Nf x[0][0][1][1][0][t] x[3][1][0][1][2][t]+bbin Nf x[0][0][0][1][2][t] x[3][1][1][1][0][t]-dc x[3][1][1][1][2][t]-unbbin x[3][1][1][1][2][t]

dx[3][2][0][0][0][t]/dt=-bbin x[0][0][0][0][1][t] x[3][2][0][0][0][t]-bbin x[0][0][0][0][2][t] x[3][2][0][0][0][t]-ac x[0][0][1][0][0][t] x[3][2][0][0][0][t]+unbbin x[3][2][0][0][1][t]+unbbin x[3][2][0][0][2][t]+dc x[3][2][1][0][0][t]

dx[3][2][0][0][1][t]/dt=bbin x[0][0][0][0][1][t] x[3][2][0][0][0][t]-unbbin x[3][2][0][0][1][t]-ac x[0][0][1][0][0][t] x[3][2][0][0][1][t]+dc x[3][2][1][0][1][t]

dx[3][2][0][0][2][t]/dt=bbin x[0][0][0][0][2][t] x[3][2][0][0][0][t]-unbbin x[3][2][0][0][2][t]-ac x[0][0][1][0][0][t] x[3][2][0][0][2][t]+dc x[3][2][1][0][2][t]

dx[3][2][0][1][0][t]/dt=-bbin Nf x[0][0][0][1][1][t] x[3][2][0][1][0][t]-bbin Nf x[0][0][0][1][2][t] x[3][2][0][1][0][t]-ac Nf x[0][0][1][1][0][t] x[3][2][0][1][0][t]+unbbin x[3][2][0][1][1][t]+unbbin x[3][2][0][1][2][t]+dc x[3][2][1][1][0][t]

dx[3][2][0][1][1][t]/dt=bbin Nf x[0][0][0][1][1][t] x[3][2][0][1][0][t]-unbbin x[3][2][0][1][1][t]-ac Nf x[0][0][1][1][0][t] x[3][2][0][1][1][t]+dc x[3][2][1][1][1][t]

dx[3][2][0][1][2][t]/dt=bbin Nf x[0][0][0][1][2][t] x[3][2][0][1][0][t]-unbbin x[3][2][0][1][2][t]-ac Nf x[0][0][1][1][0][t] x[3][2][0][1][2][t]+dc x[3][2][1][1][2][t]

dx[3][2][1][0][0][t]/dt=ac x[0][0][1][0][0][t] x[3][2][0][0][0][t]-dc x[3][2][1][0][0][t]-bbin x[0][0][0][0][1][t] x[3][2][1][0][0][t]-bbin x[0][0][0][0][2][t] x[3][2][1][0][0][t]+unbbin x[3][2][1][0][1][t]+unbbin x[3][2][1][0][2][t]

dx[3][2][1][0][1][t]/dt=ac x[0][0][1][0][0][t] x[3][2][0][0][1][t]+bbin x[0][0][0][0][1][t] x[3][2][1][0][0][t]-dc x[3][2][1][0][1][t]-unbbin x[3][2][1][0][1][t]

dx[3][2][1][0][2][t]/dt=ac x[0][0][1][0][0][t] x[3][2][0][0][2][t]+bbin x[0][0][0][0][2][t] x[3][2][1][0][0][t]-dc x[3][2][1][0][2][t]-unbbin x[3][2][1][0][2][t]

dx[3][2][1][1][0][t]/dt=ac Nf x[0][0][1][1][0][t] x[3][2][0][1][0][t]-dc x[3][2][1][1][0][t]-bbin Nf x[0][0][0][1][1][t] x[3][2][1][1][0][t]-bbin Nf x[0][0][0][1][2][t] x[3][2][1][1][0][t]+unbbin x[3][2][1][1][1][t]+unbbin x[3][2][1][1][2][t]

dx[3][2][1][1][1][t]/dt=ac Nf x[0][0][1][1][0][t] x[3][2][0][1][1][t]+bbin Nf x[0][0][0][1][1][t] x[3][2][1][1][0][t]-dc x[3][2][1][1][1][t]-unbbin x[3][2][1][1][1][t]

dx[3][2][1][1][2][t]/dt=ac Nf x[0][0][1][1][0][t] x[3][2][0][1][2][t]+bbin Nf x[0][0][0][1][2][t] x[3][2][1][1][0][t]-dc x[3][2][1][1][2][t]-unbbin x[3][2][1][1][2][t]

dx[4][0][0][0][0][t]/dt=-up x[4][0][0][0][0][t]-ac x[0][0][1][0][0][t] x[4][0][0][0][0][t]-ar x[0][1][0][0][0][t] x[4][0][0][0][0][t]-ar x[0][1][0][0][1][t] x[4][0][0][0][0][t]-ar x[0][1][0][0][2][t] x[4][0][0][0][0][t]-ar x[0][2][0][0][0][t] x[4][0][0][0][0][t]-ar x[0][2][0][0][1][t] x[4][0][0][0][0][t]-ar x[0][2][0][0][2][t] x[4][0][0][0][0][t]+ne x[4][0][0][1][0][t]+dc x[4][0][1][0][0][t]+dr x[4][1][0][0][0][t]+dr x[4][1][0][0][1][t]+dr x[4][1][0][0][2][t]+dr x[4][2][0][0][0][t]+dr x[4][2][0][0][1][t]+dr x[4][2][0][0][2][t]

dx[4][0][0][0][1][t]/dt=-ac x[0][0][1][0][0][t] x[4][0][0][0][1][t]+dc x[4][0][1][0][1][t]

dx[4][0][0][0][2][t]/dt=-ac x[0][0][1][0][0][t] x[4][0][0][0][2][t]+dc x[4][0][1][0][2][t]

dx[4][0][0][1][0][t]/dt=-ne x[4][0][0][1][0][t]-up x[4][0][0][1][0][t]-ac Nf x[0][0][1][1][0][t] x[4][0][0][1][0][t]-ar Nf x[0][1][0][1][0][t] x[4][0][0][1][0][t]-ar Nf x[0][1][0][1][1][t] x[4][0][0][1][0][t]-ar Nf x[0][1][0][1][2][t] x[4][0][0][1][0][t]-ar Nf x[0][2][0][1][0][t] x[4][0][0][1][0][t]-ar Nf x[0][2][0][1][1][t] x[4][0][0][1][0][t]-ar Nf x[0][2][0][1][2][t] x[4][0][0][1][0][t]+dc x[4][0][1][1][0][t]+dr x[4][1][0][1][0][t]+dr x[4][1][0][1][1][t]+dr x[4][1][0][1][2][t]+dr x[4][2][0][1][0][t]+dr x[4][2][0][1][1][t]+dr x[4][2][0][1][2][t]

dx[4][0][0][1][1][t]/dt=-ac Nf x[0][0][1][1][0][t] x[4][0][0][1][1][t]+dc x[4][0][1][1][1][t]

dx[4][0][0][1][2][t]/dt=-ac Nf x[0][0][1][1][0][t] x[4][0][0][1][2][t]+dc x[4][0][1][1][2][t]

dx[4][0][1][0][0][t]/dt=hot x[2][0][1][0][0][t]+hoo x[3][0][1][0][0][t]+ac x[0][0][1][0][0][t] x[4][0][0][0][0][t]-dc x[4][0][1][0][0][t]-up x[4][0][1][0][0][t]-ar x[0][1][0][0][0][t] x[4][0][1][0][0][t]-ar x[0][1][0][0][1][t] x[4][0][1][0][0][t]-ar x[0][1][0][0][2][t] x[4][0][1][0][0][t]-ar x[0][2][0][0][0][t] x[4][0][1][0][0][t]-ar x[0][2][0][0][1][t] x[4][0][1][0][0][t]-ar x[0][2][0][0][2][t] x[4][0][1][0][0][t]+ne x[4][0][1][1][0][t]+dr x[4][1][1][0][0][t]+dr x[4][1][1][0][1][t]+dr x[4][1][1][0][2][t]+dr x[4][2][1][0][0][t]+dr x[4][2][1][0][1][t]+dr x[4][2][1][0][2][t]

dx[4][0][1][0][1][t]/dt=hot x[2][0][1][0][1][t]+ac x[0][0][1][0][0][t] x[4][0][0][0][1][t]-dc x[4][0][1][0][1][t]

dx[4][0][1][0][2][t]/dt=hot x[2][0][1][0][2][t]+ac x[0][0][1][0][0][t] x[4][0][0][0][2][t]-dc x[4][0][1][0][2][t]

dx[4][0][1][1][0][t]/dt=hot x[2][0][1][1][0][t]+ac Nf x[0][0][1][1][0][t] x[4][0][0][1][0][t]-dc x[4][0][1][1][0][t]-ne x[4][0][1][1][0][t]-up x[4][0][1][1][0][t]-ar Nf x[0][1][0][1][0][t] x[4][0][1][1][0][t]-ar Nf x[0][1][0][1][1][t] x[4][0][1][1][0][t]-ar Nf x[0][1][0][1][2][t] x[4][0][1][1][0][t]-ar Nf x[0][2][0][1][0][t] x[4][0][1][1][0][t]-ar Nf x[0][2][0][1][1][t] x[4][0][1][1][0][t]-ar Nf x[0][2][0][1][2][t] x[4][0][1][1][0][t]+dr x[4][1][1][1][0][t]+dr x[4][1][1][1][1][t]+dr x[4][1][1][1][2][t]+dr x[4][2][1][1][0][t]+dr x[4][2][1][1][1][t]+dr x[4][2][1][1][2][t]

dx[4][0][1][1][1][t]/dt=hot x[2][0][1][1][1][t]+ac Nf x[0][0][1][1][0][t] x[4][0][0][1][1][t]-dc x[4][0][1][1][1][t]

dx[4][0][1][1][2][t]/dt=hot x[2][0][1][1][2][t]+ac Nf x[0][0][1][1][0][t] x[4][0][0][1][2][t]-dc x[4][0][1][1][2][t]

dx[4][1][0][0][0][t]/dt=ar x[0][1][0][0][0][t] x[4][0][0][0][0][t]-dr x[4][1][0][0][0][t]-bbin x[0][0][0][0][1][t] x[4][1][0][0][0][t]-bbin x[0][0][0][0][2][t] x[4][1][0][0][0][t]-ac x[0][0][1][0][0][t] x[4][1][0][0][0][t]+unbbin x[4][1][0][0][1][t]+unbbin x[4][1][0][0][2][t]+ne x[4][1][0][1][0][t]+dc x[4][1][1][0][0][t]

dx[4][1][0][0][1][t]/dt=ar x[0][1][0][0][1][t] x[4][0][0][0][0][t]+bbin x[0][0][0][0][1][t] x[4][1][0][0][0][t]-dr x[4][1][0][0][1][t]-unbbin x[4][1][0][0][1][t]-ac x[0][0][1][0][0][t] x[4][1][0][0][1][t]+dc x[4][1][1][0][1][t]

dx[4][1][0][0][2][t]/dt=ar x[0][1][0][0][2][t] x[4][0][0][0][0][t]+bbin x[0][0][0][0][2][t] x[4][1][0][0][0][t]-dr x[4][1][0][0][2][t]-unbbin x[4][1][0][0][2][t]-ac x[0][0][1][0][0][t] x[4][1][0][0][2][t]+dc x[4][1][1][0][2][t]

dx[4][1][0][1][0][t]/dt=ar Nf x[0][1][0][1][0][t] x[4][0][0][1][0][t]-dr x[4][1][0][1][0][t]-ne x[4][1][0][1][0][t]-bbin Nf x[0][0][0][1][1][t] x[4][1][0][1][0][t]-bbin Nf x[0][0][0][1][2][t] x[4][1][0][1][0][t]-ac Nf x[0][0][1][1][0][t] x[4][1][0][1][0][t]+unbbin x[4][1][0][1][1][t]+unbbin x[4][1][0][1][2][t]+dc x[4][1][1][1][0][t]

dx[4][1][0][1][1][t]/dt=ar Nf x[0][1][0][1][1][t] x[4][0][0][1][0][t]+bbin Nf x[0][0][0][1][1][t] x[4][1][0][1][0][t]-dr x[4][1][0][1][1][t]-unbbin x[4][1][0][1][1][t]-ac Nf x[0][0][1][1][0][t] x[4][1][0][1][1][t]+dc x[4][1][1][1][1][t]

dx[4][1][0][1][2][t]/dt=ar Nf x[0][1][0][1][2][t] x[4][0][0][1][0][t]+bbin Nf x[0][0][0][1][2][t] x[4][1][0][1][0][t]-dr x[4][1][0][1][2][t]-unbbin x[4][1][0][1][2][t]-ac Nf x[0][0][1][1][0][t] x[4][1][0][1][2][t]+dc x[4][1][1][1][2][t]

dx[4][1][1][0][0][t]/dt=hot x[2][1][1][0][0][t]+ar x[0][1][0][0][0][t] x[4][0][1][0][0][t]+ac x[0][0][1][0][0][t] x[4][1][0][0][0][t]-dc x[4][1][1][0][0][t]-dr x[4][1][1][0][0][t]-bbin x[0][0][0][0][1][t] x[4][1][1][0][0][t]-bbin x[0][0][0][0][2][t] x[4][1][1][0][0][t]+unbbin x[4][1][1][0][1][t]+unbbin x[4][1][1][0][2][t]+ne x[4][1][1][1][0][t]

dx[4][1][1][0][1][t]/dt=hot x[2][1][1][0][1][t]+ar x[0][1][0][0][1][t] x[4][0][1][0][0][t]+ac x[0][0][1][0][0][t] x[4][1][0][0][1][t]+bbin x[0][0][0][0][1][t] x[4][1][1][0][0][t]-dc x[4][1][1][0][1][t]-dr x[4][1][1][0][1][t]-unbbin x[4][1][1][0][1][t]

dx[4][1][1][0][2][t]/dt=hot x[2][1][1][0][2][t]+ar x[0][1][0][0][2][t] x[4][0][1][0][0][t]+ac x[0][0][1][0][0][t] x[4][1][0][0][2][t]+bbin x[0][0][0][0][2][t] x[4][1][1][0][0][t]-dc x[4][1][1][0][2][t]-dr x[4][1][1][0][2][t]-unbbin x[4][1][1][0][2][t]

dx[4][1][1][1][0][t]/dt=hot x[2][1][1][1][0][t]+ar Nf x[0][1][0][1][0][t] x[4][0][1][1][0][t]+ac Nf x[0][0][1][1][0][t] x[4][1][0][1][0][t]-dc x[4][1][1][1][0][t]-dr x[4][1][1][1][0][t]-ne x[4][1][1][1][0][t]-bbin Nf x[0][0][0][1][1][t] x[4][1][1][1][0][t]-bbin Nf x[0][0][0][1][2][t] x[4][1][1][1][0][t]+unbbin x[4][1][1][1][1][t]+unbbin x[4][1][1][1][2][t]

dx[4][1][1][1][1][t]/dt=hot x[2][1][1][1][1][t]+ar Nf x[0][1][0][1][1][t] x[4][0][1][1][0][t]+ac Nf x[0][0][1][1][0][t] x[4][1][0][1][1][t]+bbin Nf x[0][0][0][1][1][t] x[4][1][1][1][0][t]-dc x[4][1][1][1][1][t]-dr x[4][1][1][1][1][t]-unbbin x[4][1][1][1][1][t]

dx[4][1][1][1][2][t]/dt=hot x[2][1][1][1][2][t]+ar Nf x[0][1][0][1][2][t] x[4][0][1][1][0][t]+ac Nf x[0][0][1][1][0][t] x[4][1][0][1][2][t]+bbin Nf x[0][0][0][1][2][t] x[4][1][1][1][0][t]-dc x[4][1][1][1][2][t]-dr x[4][1][1][1][2][t]-unbbin x[4][1][1][1][2][t]

dx[4][2][0][0][0][t]/dt=ar x[0][2][0][0][0][t] x[4][0][0][0][0][t]-dr x[4][2][0][0][0][t]-bbin x[0][0][0][0][1][t] x[4][2][0][0][0][t]-bbin x[0][0][0][0][2][t] x[4][2][0][0][0][t]-ac x[0][0][1][0][0][t] x[4][2][0][0][0][t]+unbbin x[4][2][0][0][1][t]+unbbin x[4][2][0][0][2][t]+ne x[4][2][0][1][0][t]+dc x[4][2][1][0][0][t]

dx[4][2][0][0][1][t]/dt=ar x[0][2][0][0][1][t] x[4][0][0][0][0][t]+bbin x[0][0][0][0][1][t] x[4][2][0][0][0][t]-dr x[4][2][0][0][1][t]-unbbin x[4][2][0][0][1][t]-ac x[0][0][1][0][0][t] x[4][2][0][0][1][t]+dc x[4][2][1][0][1][t]

dx[4][2][0][0][2][t]/dt=ar x[0][2][0][0][2][t] x[4][0][0][0][0][t]+bbin x[0][0][0][0][2][t] x[4][2][0][0][0][t]-dr x[4][2][0][0][2][t]-unbbin x[4][2][0][0][2][t]-ac x[0][0][1][0][0][t] x[4][2][0][0][2][t]+dc x[4][2][1][0][2][t]

dx[4][2][0][1][0][t]/dt=ar Nf x[0][2][0][1][0][t] x[4][0][0][1][0][t]-dr x[4][2][0][1][0][t]-ne x[4][2][0][1][0][t]-bbin Nf x[0][0][0][1][1][t] x[4][2][0][1][0][t]-bbin Nf x[0][0][0][1][2][t] x[4][2][0][1][0][t]-ac Nf x[0][0][1][1][0][t] x[4][2][0][1][0][t]+unbbin x[4][2][0][1][1][t]+unbbin x[4][2][0][1][2][t]+dc x[4][2][1][1][0][t]

dx[4][2][0][1][1][t]/dt=ar Nf x[0][2][0][1][1][t] x[4][0][0][1][0][t]+bbin Nf x[0][0][0][1][1][t] x[4][2][0][1][0][t]-dr x[4][2][0][1][1][t]-unbbin x[4][2][0][1][1][t]-ac Nf x[0][0][1][1][0][t] x[4][2][0][1][1][t]+dc x[4][2][1][1][1][t]

dx[4][2][0][1][2][t]/dt=ar Nf x[0][2][0][1][2][t] x[4][0][0][1][0][t]+bbin Nf x[0][0][0][1][2][t] x[4][2][0][1][0][t]-dr x[4][2][0][1][2][t]-unbbin x[4][2][0][1][2][t]-ac Nf x[0][0][1][1][0][t] x[4][2][0][1][2][t]+dc x[4][2][1][1][2][t]

dx[4][2][1][0][0][t]/dt=hot x[2][2][1][0][0][t]+ar x[0][2][0][0][0][t] x[4][0][1][0][0][t]+ac x[0][0][1][0][0][t] x[4][2][0][0][0][t]-dc x[4][2][1][0][0][t]-dr x[4][2][1][0][0][t]-bbin x[0][0][0][0][1][t] x[4][2][1][0][0][t]-bbin x[0][0][0][0][2][t] x[4][2][1][0][0][t]+unbbin x[4][2][1][0][1][t]+unbbin x[4][2][1][0][2][t]+ne x[4][2][1][1][0][t]

dx[4][2][1][0][1][t]/dt=hot x[2][2][1][0][1][t]+ar x[0][2][0][0][1][t] x[4][0][1][0][0][t]+ac x[0][0][1][0][0][t] x[4][2][0][0][1][t]+bbin x[0][0][0][0][1][t] x[4][2][1][0][0][t]-dc x[4][2][1][0][1][t]-dr x[4][2][1][0][1][t]-unbbin x[4][2][1][0][1][t]

dx[4][2][1][0][2][t]/dt=hot x[2][2][1][0][2][t]+ar x[0][2][0][0][2][t] x[4][0][1][0][0][t]+ac x[0][0][1][0][0][t] x[4][2][0][0][2][t]+bbin x[0][0][0][0][2][t] x[4][2][1][0][0][t]-dc x[4][2][1][0][2][t]-dr x[4][2][1][0][2][t]-unbbin x[4][2][1][0][2][t]

dx[4][2][1][1][0][t]/dt=hot x[2][2][1][1][0][t]+ar Nf x[0][2][0][1][0][t] x[4][0][1][1][0][t]+ac Nf x[0][0][1][1][0][t] x[4][2][0][1][0][t]-dc x[4][2][1][1][0][t]-dr x[4][2][1][1][0][t]-ne x[4][2][1][1][0][t]-bbin Nf x[0][0][0][1][1][t] x[4][2][1][1][0][t]-bbin Nf x[0][0][0][1][2][t] x[4][2][1][1][0][t]+unbbin x[4][2][1][1][1][t]+unbbin x[4][2][1][1][2][t]

dx[4][2][1][1][1][t]/dt=hot x[2][2][1][1][1][t]+ar Nf x[0][2][0][1][1][t] x[4][0][1][1][0][t]+ac Nf x[0][0][1][1][0][t] x[4][2][0][1][1][t]+bbin Nf x[0][0][0][1][1][t] x[4][2][1][1][0][t]-dc x[4][2][1][1][1][t]-dr x[4][2][1][1][1][t]-unbbin x[4][2][1][1][1][t]

dx[4][2][1][1][2][t]/dt=hot x[2][2][1][1][2][t]+ar Nf x[0][2][0][1][2][t] x[4][0][1][1][0][t]+ac Nf x[0][0][1][1][0][t] x[4][2][0][1][2][t]+bbin Nf x[0][0][0][1][2][t] x[4][2][1][1][0][t]-dc x[4][2][1][1][2][t]-dr x[4][2][1][1][2][t]-unbbin x[4][2][1][1][2][t]

dx[5][0][0][0][0][t]/dt=tlp McPt[t]-upu x[5][0][0][0][0][t]-ac x[0][0][1][0][0][t] x[5][0][0][0][0][t]+dc x[5][0][1][0][0][t]

dx[5][0][0][0][1][t]/dt=-ac x[0][0][1][0][0][t] x[5][0][0][0][1][t]+dc x[5][0][1][0][1][t]

dx[5][0][0][0][2][t]/dt=-ac x[0][0][1][0][0][t] x[5][0][0][0][2][t]+dc x[5][0][1][0][2][t]

dx[5][0][0][1][0][t]/dt=-ac Nf x[0][0][1][1][0][t] x[5][0][0][1][0][t]+dc x[5][0][1][1][0][t]

dx[5][0][0][1][1][t]/dt=-ac Nf x[0][0][1][1][0][t] x[5][0][0][1][1][t]+dc x[5][0][1][1][1][t]

dx[5][0][0][1][2][t]/dt=-ac Nf x[0][0][1][1][0][t] x[5][0][0][1][2][t]+dc x[5][0][1][1][2][t]

dx[5][0][1][0][0][t]/dt=ac x[0][0][1][0][0][t] x[5][0][0][0][0][t]-dc x[5][0][1][0][0][t]-hto x[5][0][1][0][0][t]-upu x[5][0][1][0][0][t]

dx[5][0][1][0][1][t]/dt=ac x[0][0][1][0][0][t] x[5][0][0][0][1][t]-dc x[5][0][1][0][1][t]

dx[5][0][1][0][2][t]/dt=ac x[0][0][1][0][0][t] x[5][0][0][0][2][t]-dc x[5][0][1][0][2][t]

dx[5][0][1][1][0][t]/dt=ac Nf x[0][0][1][1][0][t] x[5][0][0][1][0][t]-dc x[5][0][1][1][0][t]

dx[5][0][1][1][1][t]/dt=ac Nf x[0][0][1][1][0][t] x[5][0][0][1][1][t]-dc x[5][0][1][1][1][t]

dx[5][0][1][1][2][t]/dt=ac Nf x[0][0][1][1][0][t] x[5][0][0][1][2][t]-dc x[5][0][1][1][2][t]

dx[5][1][0][0][0][t]/dt=-bbin x[0][0][0][0][1][t] x[5][1][0][0][0][t]-bbin x[0][0][0][0][2][t] x[5][1][0][0][0][t]-ac x[0][0][1][0][0][t] x[5][1][0][0][0][t]+unbbin x[5][1][0][0][1][t]+unbbin x[5][1][0][0][2][t]+dc x[5][1][1][0][0][t]

dx[5][1][0][0][1][t]/dt=bbin x[0][0][0][0][1][t] x[5][1][0][0][0][t]-unbbin x[5][1][0][0][1][t]-ac x[0][0][1][0][0][t] x[5][1][0][0][1][t]+dc x[5][1][1][0][1][t]

dx[5][1][0][0][2][t]/dt=bbin x[0][0][0][0][2][t] x[5][1][0][0][0][t]-unbbin x[5][1][0][0][2][t]-ac x[0][0][1][0][0][t] x[5][1][0][0][2][t]+dc x[5][1][1][0][2][t]

dx[5][1][0][1][0][t]/dt=-bbin Nf x[0][0][0][1][1][t] x[5][1][0][1][0][t]-bbin Nf x[0][0][0][1][2][t] x[5][1][0][1][0][t]-ac Nf x[0][0][1][1][0][t] x[5][1][0][1][0][t]+unbbin x[5][1][0][1][1][t]+unbbin x[5][1][0][1][2][t]+dc x[5][1][1][1][0][t]

dx[5][1][0][1][1][t]/dt=bbin Nf x[0][0][0][1][1][t] x[5][1][0][1][0][t]-unbbin x[5][1][0][1][1][t]-ac Nf x[0][0][1][1][0][t] x[5][1][0][1][1][t]+dc x[5][1][1][1][1][t]

dx[5][1][0][1][2][t]/dt=bbin Nf x[0][0][0][1][2][t] x[5][1][0][1][0][t]-unbbin x[5][1][0][1][2][t]-ac Nf x[0][0][1][1][0][t] x[5][1][0][1][2][t]+dc x[5][1][1][1][2][t]

dx[5][1][1][0][0][t]/dt=ac x[0][0][1][0][0][t] x[5][1][0][0][0][t]-dc x[5][1][1][0][0][t]-bbin x[0][0][0][0][1][t] x[5][1][1][0][0][t]-bbin x[0][0][0][0][2][t] x[5][1][1][0][0][t]+unbbin x[5][1][1][0][1][t]+unbbin x[5][1][1][0][2][t]

dx[5][1][1][0][1][t]/dt=ac x[0][0][1][0][0][t] x[5][1][0][0][1][t]+bbin x[0][0][0][0][1][t] x[5][1][1][0][0][t]-dc x[5][1][1][0][1][t]-unbbin x[5][1][1][0][1][t]

dx[5][1][1][0][2][t]/dt=ac x[0][0][1][0][0][t] x[5][1][0][0][2][t]+bbin x[0][0][0][0][2][t] x[5][1][1][0][0][t]-dc x[5][1][1][0][2][t]-unbbin x[5][1][1][0][2][t]

dx[5][1][1][1][0][t]/dt=ac Nf x[0][0][1][1][0][t] x[5][1][0][1][0][t]-dc x[5][1][1][1][0][t]-bbin Nf x[0][0][0][1][1][t] x[5][1][1][1][0][t]-bbin Nf x[0][0][0][1][2][t] x[5][1][1][1][0][t]+unbbin x[5][1][1][1][1][t]+unbbin x[5][1][1][1][2][t]

dx[5][1][1][1][1][t]/dt=ac Nf x[0][0][1][1][0][t] x[5][1][0][1][1][t]+bbin Nf x[0][0][0][1][1][t] x[5][1][1][1][0][t]-dc x[5][1][1][1][1][t]-unbbin x[5][1][1][1][1][t]

dx[5][1][1][1][2][t]/dt=ac Nf x[0][0][1][1][0][t] x[5][1][0][1][2][t]+bbin Nf x[0][0][0][1][2][t] x[5][1][1][1][0][t]-dc x[5][1][1][1][2][t]-unbbin x[5][1][1][1][2][t]

dx[5][2][0][0][0][t]/dt=-bbin x[0][0][0][0][1][t] x[5][2][0][0][0][t]-bbin x[0][0][0][0][2][t] x[5][2][0][0][0][t]-ac x[0][0][1][0][0][t] x[5][2][0][0][0][t]+unbbin x[5][2][0][0][1][t]+unbbin x[5][2][0][0][2][t]+dc x[5][2][1][0][0][t]

dx[5][2][0][0][1][t]/dt=bbin x[0][0][0][0][1][t] x[5][2][0][0][0][t]-unbbin x[5][2][0][0][1][t]-ac x[0][0][1][0][0][t] x[5][2][0][0][1][t]+dc x[5][2][1][0][1][t]

dx[5][2][0][0][2][t]/dt=bbin x[0][0][0][0][2][t] x[5][2][0][0][0][t]-unbbin x[5][2][0][0][2][t]-ac x[0][0][1][0][0][t] x[5][2][0][0][2][t]+dc x[5][2][1][0][2][t]

dx[5][2][0][1][0][t]/dt=-bbin Nf x[0][0][0][1][1][t] x[5][2][0][1][0][t]-bbin Nf x[0][0][0][1][2][t] x[5][2][0][1][0][t]-ac Nf x[0][0][1][1][0][t] x[5][2][0][1][0][t]+unbbin x[5][2][0][1][1][t]+unbbin x[5][2][0][1][2][t]+dc x[5][2][1][1][0][t]

dx[5][2][0][1][1][t]/dt=bbin Nf x[0][0][0][1][1][t] x[5][2][0][1][0][t]-unbbin x[5][2][0][1][1][t]-ac Nf x[0][0][1][1][0][t] x[5][2][0][1][1][t]+dc x[5][2][1][1][1][t]

dx[5][2][0][1][2][t]/dt=bbin Nf x[0][0][0][1][2][t] x[5][2][0][1][0][t]-unbbin x[5][2][0][1][2][t]-ac Nf x[0][0][1][1][0][t] x[5][2][0][1][2][t]+dc x[5][2][1][1][2][t]

dx[5][2][1][0][0][t]/dt=ac x[0][0][1][0][0][t] x[5][2][0][0][0][t]-dc x[5][2][1][0][0][t]-bbin x[0][0][0][0][1][t] x[5][2][1][0][0][t]-bbin x[0][0][0][0][2][t] x[5][2][1][0][0][t]+unbbin x[5][2][1][0][1][t]+unbbin x[5][2][1][0][2][t]

dx[5][2][1][0][1][t]/dt=ac x[0][0][1][0][0][t] x[5][2][0][0][1][t]+bbin x[0][0][0][0][1][t] x[5][2][1][0][0][t]-dc x[5][2][1][0][1][t]-unbbin x[5][2][1][0][1][t]

dx[5][2][1][0][2][t]/dt=ac x[0][0][1][0][0][t] x[5][2][0][0][2][t]+bbin x[0][0][0][0][2][t] x[5][2][1][0][0][t]-dc x[5][2][1][0][2][t]-unbbin x[5][2][1][0][2][t]

dx[5][2][1][1][0][t]/dt=ac Nf x[0][0][1][1][0][t] x[5][2][0][1][0][t]-dc x[5][2][1][1][0][t]-bbin Nf x[0][0][0][1][1][t] x[5][2][1][1][0][t]-bbin Nf x[0][0][0][1][2][t] x[5][2][1][1][0][t]+unbbin x[5][2][1][1][1][t]+unbbin x[5][2][1][1][2][t]

dx[5][2][1][1][1][t]/dt=ac Nf x[0][0][1][1][0][t] x[5][2][0][1][1][t]+bbin Nf x[0][0][0][1][1][t] x[5][2][1][1][0][t]-dc x[5][2][1][1][1][t]-unbbin x[5][2][1][1][1][t]

dx[5][2][1][1][2][t]/dt=ac Nf x[0][0][1][1][0][t] x[5][2][0][1][2][t]+bbin Nf x[0][0][0][1][2][t] x[5][2][1][1][0][t]-dc x[5][2][1][1][2][t]-unbbin x[5][2][1][1][2][t]

dx[6][0][0][0][0][t]/dt=-nl x[6][0][0][0][0][t]-up x[6][0][0][0][0][t]-ac x[0][0][1][0][0][t] x[6][0][0][0][0][t]-ar x[0][1][0][0][0][t] x[6][0][0][0][0][t]-ar x[0][1][0][0][1][t] x[6][0][0][0][0][t]-ar x[0][1][0][0][2][t] x[6][0][0][0][0][t]-ar x[0][2][0][0][0][t] x[6][0][0][0][0][t]-ar x[0][2][0][0][1][t] x[6][0][0][0][0][t]-ar x[0][2][0][0][2][t] x[6][0][0][0][0][t]+ne x[6][0][0][1][0][t]+dc x[6][0][1][0][0][t]+dr x[6][1][0][0][0][t]+dr x[6][1][0][0][1][t]+dr x[6][1][0][0][2][t]+dr x[6][2][0][0][0][t]+dr x[6][2][0][0][1][t]+dr x[6][2][0][0][2][t]

dx[6][0][0][0][1][t]/dt=-nl x[6][0][0][0][1][t]-ac x[0][0][1][0][0][t] x[6][0][0][0][1][t]+dc x[6][0][1][0][1][t]

dx[6][0][0][0][2][t]/dt=-nl x[6][0][0][0][2][t]-ac x[0][0][1][0][0][t] x[6][0][0][0][2][t]+dc x[6][0][1][0][2][t]

dx[6][0][0][1][0][t]/dt=nl x[6][0][0][0][0][t]-ne x[6][0][0][1][0][t]-up x[6][0][0][1][0][t]-ac Nf x[0][0][1][1][0][t] x[6][0][0][1][0][t]-ar Nf x[0][1][0][1][0][t] x[6][0][0][1][0][t]-ar Nf x[0][1][0][1][1][t] x[6][0][0][1][0][t]-ar Nf x[0][1][0][1][2][t] x[6][0][0][1][0][t]-ar Nf x[0][2][0][1][0][t] x[6][0][0][1][0][t]-ar Nf x[0][2][0][1][1][t] x[6][0][0][1][0][t]-ar Nf x[0][2][0][1][2][t] x[6][0][0][1][0][t]+dc x[6][0][1][1][0][t]+dr x[6][1][0][1][0][t]+dr x[6][1][0][1][1][t]+dr x[6][1][0][1][2][t]+dr x[6][2][0][1][0][t]+dr x[6][2][0][1][1][t]+dr x[6][2][0][1][2][t]

dx[6][0][0][1][1][t]/dt=nl x[6][0][0][0][1][t]-ac Nf x[0][0][1][1][0][t] x[6][0][0][1][1][t]+dc x[6][0][1][1][1][t]

dx[6][0][0][1][2][t]/dt=nl x[6][0][0][0][2][t]-ac Nf x[0][0][1][1][0][t] x[6][0][0][1][2][t]+dc x[6][0][1][1][2][t]

dx[6][0][1][0][0][t]/dt=hto x[5][0][1][0][0][t]+ac x[0][0][1][0][0][t] x[6][0][0][0][0][t]-dc x[6][0][1][0][0][t]-nl x[6][0][1][0][0][t]-up x[6][0][1][0][0][t]-ar x[0][1][0][0][0][t] x[6][0][1][0][0][t]-ar x[0][1][0][0][1][t] x[6][0][1][0][0][t]-ar x[0][1][0][0][2][t] x[6][0][1][0][0][t]-ar x[0][2][0][0][0][t] x[6][0][1][0][0][t]-ar x[0][2][0][0][1][t] x[6][0][1][0][0][t]-ar x[0][2][0][0][2][t] x[6][0][1][0][0][t]+ne x[6][0][1][1][0][t]+dr x[6][1][1][0][0][t]+dr x[6][1][1][0][1][t]+dr x[6][1][1][0][2][t]+dr x[6][2][1][0][0][t]+dr x[6][2][1][0][1][t]+dr x[6][2][1][0][2][t]

dx[6][0][1][0][1][t]/dt=ac x[0][0][1][0][0][t] x[6][0][0][0][1][t]-dc x[6][0][1][0][1][t]-nl x[6][0][1][0][1][t]

dx[6][0][1][0][2][t]/dt=ac x[0][0][1][0][0][t] x[6][0][0][0][2][t]-dc x[6][0][1][0][2][t]-nl x[6][0][1][0][2][t]

dx[6][0][1][1][0][t]/dt=ac Nf x[0][0][1][1][0][t] x[6][0][0][1][0][t]+nl x[6][0][1][0][0][t]-dc x[6][0][1][1][0][t]-ne x[6][0][1][1][0][t]-up x[6][0][1][1][0][t]-ar Nf x[0][1][0][1][0][t] x[6][0][1][1][0][t]-ar Nf x[0][1][0][1][1][t] x[6][0][1][1][0][t]-ar Nf x[0][1][0][1][2][t] x[6][0][1][1][0][t]-ar Nf x[0][2][0][1][0][t] x[6][0][1][1][0][t]-ar Nf x[0][2][0][1][1][t] x[6][0][1][1][0][t]-ar Nf x[0][2][0][1][2][t] x[6][0][1][1][0][t]+dr x[6][1][1][1][0][t]+dr x[6][1][1][1][1][t]+dr x[6][1][1][1][2][t]+dr x[6][2][1][1][0][t]+dr x[6][2][1][1][1][t]+dr x[6][2][1][1][2][t]

dx[6][0][1][1][1][t]/dt=ac Nf x[0][0][1][1][0][t] x[6][0][0][1][1][t]+nl x[6][0][1][0][1][t]-dc x[6][0][1][1][1][t]

dx[6][0][1][1][2][t]/dt=ac Nf x[0][0][1][1][0][t] x[6][0][0][1][2][t]+nl x[6][0][1][0][2][t]-dc x[6][0][1][1][2][t]

dx[6][1][0][0][0][t]/dt=ar x[0][1][0][0][0][t] x[6][0][0][0][0][t]-dr x[6][1][0][0][0][t]-nl x[6][1][0][0][0][t]-bbin x[0][0][0][0][1][t] x[6][1][0][0][0][t]-bbin x[0][0][0][0][2][t] x[6][1][0][0][0][t]-ac x[0][0][1][0][0][t] x[6][1][0][0][0][t]+unbbin x[6][1][0][0][1][t]+unbbin x[6][1][0][0][2][t]+ne x[6][1][0][1][0][t]+dc x[6][1][1][0][0][t]

dx[6][1][0][0][1][t]/dt=ar x[0][1][0][0][1][t] x[6][0][0][0][0][t]+bbin x[0][0][0][0][1][t] x[6][1][0][0][0][t]-dr x[6][1][0][0][1][t]-nl x[6][1][0][0][1][t]-unbbin x[6][1][0][0][1][t]-ac x[0][0][1][0][0][t] x[6][1][0][0][1][t]+dc x[6][1][1][0][1][t]

dx[6][1][0][0][2][t]/dt=ar x[0][1][0][0][2][t] x[6][0][0][0][0][t]+bbin x[0][0][0][0][2][t] x[6][1][0][0][0][t]-dr x[6][1][0][0][2][t]-nl x[6][1][0][0][2][t]-unbbin x[6][1][0][0][2][t]-ac x[0][0][1][0][0][t] x[6][1][0][0][2][t]+dc x[6][1][1][0][2][t]

dx[6][1][0][1][0][t]/dt=ar Nf x[0][1][0][1][0][t] x[6][0][0][1][0][t]+nl x[6][1][0][0][0][t]-dr x[6][1][0][1][0][t]-ne x[6][1][0][1][0][t]-bbin Nf x[0][0][0][1][1][t] x[6][1][0][1][0][t]-bbin Nf x[0][0][0][1][2][t] x[6][1][0][1][0][t]-ac Nf x[0][0][1][1][0][t] x[6][1][0][1][0][t]+unbbin x[6][1][0][1][1][t]+unbbin x[6][1][0][1][2][t]+dc x[6][1][1][1][0][t]

dx[6][1][0][1][1][t]/dt=ar Nf x[0][1][0][1][1][t] x[6][0][0][1][0][t]+nl x[6][1][0][0][1][t]+bbin Nf x[0][0][0][1][1][t] x[6][1][0][1][0][t]-dr x[6][1][0][1][1][t]-unbbin x[6][1][0][1][1][t]-ac Nf x[0][0][1][1][0][t] x[6][1][0][1][1][t]+dc x[6][1][1][1][1][t]

dx[6][1][0][1][2][t]/dt=ar Nf x[0][1][0][1][2][t] x[6][0][0][1][0][t]+nl x[6][1][0][0][2][t]+bbin Nf x[0][0][0][1][2][t] x[6][1][0][1][0][t]-dr x[6][1][0][1][2][t]-unbbin x[6][1][0][1][2][t]-ac Nf x[0][0][1][1][0][t] x[6][1][0][1][2][t]+dc x[6][1][1][1][2][t]

dx[6][1][1][0][0][t]/dt=ar x[0][1][0][0][0][t] x[6][0][1][0][0][t]+ac x[0][0][1][0][0][t] x[6][1][0][0][0][t]-dc x[6][1][1][0][0][t]-dr x[6][1][1][0][0][t]-nl x[6][1][1][0][0][t]-bbin x[0][0][0][0][1][t] x[6][1][1][0][0][t]-bbin x[0][0][0][0][2][t] x[6][1][1][0][0][t]+unbbin x[6][1][1][0][1][t]+unbbin x[6][1][1][0][2][t]+ne x[6][1][1][1][0][t]

dx[6][1][1][0][1][t]/dt=ar x[0][1][0][0][1][t] x[6][0][1][0][0][t]+ac x[0][0][1][0][0][t] x[6][1][0][0][1][t]+bbin x[0][0][0][0][1][t] x[6][1][1][0][0][t]-dc x[6][1][1][0][1][t]-dr x[6][1][1][0][1][t]-nl x[6][1][1][0][1][t]-unbbin x[6][1][1][0][1][t]

dx[6][1][1][0][2][t]/dt=ar x[0][1][0][0][2][t] x[6][0][1][0][0][t]+ac x[0][0][1][0][0][t] x[6][1][0][0][2][t]+bbin x[0][0][0][0][2][t] x[6][1][1][0][0][t]-dc x[6][1][1][0][2][t]-dr x[6][1][1][0][2][t]-nl x[6][1][1][0][2][t]-unbbin x[6][1][1][0][2][t]

dx[6][1][1][1][0][t]/dt=ar Nf x[0][1][0][1][0][t] x[6][0][1][1][0][t]+ac Nf x[0][0][1][1][0][t] x[6][1][0][1][0][t]+nl x[6][1][1][0][0][t]-dc x[6][1][1][1][0][t]-dr x[6][1][1][1][0][t]-ne x[6][1][1][1][0][t]-bbin Nf x[0][0][0][1][1][t] x[6][1][1][1][0][t]-bbin Nf x[0][0][0][1][2][t] x[6][1][1][1][0][t]+unbbin x[6][1][1][1][1][t]+unbbin x[6][1][1][1][2][t]

dx[6][1][1][1][1][t]/dt=ar Nf x[0][1][0][1][1][t] x[6][0][1][1][0][t]+ac Nf x[0][0][1][1][0][t] x[6][1][0][1][1][t]+nl x[6][1][1][0][1][t]+bbin Nf x[0][0][0][1][1][t] x[6][1][1][1][0][t]-dc x[6][1][1][1][1][t]-dr x[6][1][1][1][1][t]-unbbin x[6][1][1][1][1][t]

dx[6][1][1][1][2][t]/dt=ar Nf x[0][1][0][1][2][t] x[6][0][1][1][0][t]+ac Nf x[0][0][1][1][0][t] x[6][1][0][1][2][t]+nl x[6][1][1][0][2][t]+bbin Nf x[0][0][0][1][2][t] x[6][1][1][1][0][t]-dc x[6][1][1][1][2][t]-dr x[6][1][1][1][2][t]-unbbin x[6][1][1][1][2][t]

dx[6][2][0][0][0][t]/dt=ar x[0][2][0][0][0][t] x[6][0][0][0][0][t]-dr x[6][2][0][0][0][t]-nl x[6][2][0][0][0][t]-bbin x[0][0][0][0][1][t] x[6][2][0][0][0][t]-bbin x[0][0][0][0][2][t] x[6][2][0][0][0][t]-ac x[0][0][1][0][0][t] x[6][2][0][0][0][t]+unbbin x[6][2][0][0][1][t]+unbbin x[6][2][0][0][2][t]+ne x[6][2][0][1][0][t]+dc x[6][2][1][0][0][t]

dx[6][2][0][0][1][t]/dt=ar x[0][2][0][0][1][t] x[6][0][0][0][0][t]+bbin x[0][0][0][0][1][t] x[6][2][0][0][0][t]-dr x[6][2][0][0][1][t]-nl x[6][2][0][0][1][t]-unbbin x[6][2][0][0][1][t]-ac x[0][0][1][0][0][t] x[6][2][0][0][1][t]+dc x[6][2][1][0][1][t]

dx[6][2][0][0][2][t]/dt=ar x[0][2][0][0][2][t] x[6][0][0][0][0][t]+bbin x[0][0][0][0][2][t] x[6][2][0][0][0][t]-dr x[6][2][0][0][2][t]-nl x[6][2][0][0][2][t]-unbbin x[6][2][0][0][2][t]-ac x[0][0][1][0][0][t] x[6][2][0][0][2][t]+dc x[6][2][1][0][2][t]

dx[6][2][0][1][0][t]/dt=ar Nf x[0][2][0][1][0][t] x[6][0][0][1][0][t]+nl x[6][2][0][0][0][t]-dr x[6][2][0][1][0][t]-ne x[6][2][0][1][0][t]-bbin Nf x[0][0][0][1][1][t] x[6][2][0][1][0][t]-bbin Nf x[0][0][0][1][2][t] x[6][2][0][1][0][t]-ac Nf x[0][0][1][1][0][t] x[6][2][0][1][0][t]+unbbin x[6][2][0][1][1][t]+unbbin x[6][2][0][1][2][t]+dc x[6][2][1][1][0][t]

dx[6][2][0][1][1][t]/dt=ar Nf x[0][2][0][1][1][t] x[6][0][0][1][0][t]+nl x[6][2][0][0][1][t]+bbin Nf x[0][0][0][1][1][t] x[6][2][0][1][0][t]-dr x[6][2][0][1][1][t]-unbbin x[6][2][0][1][1][t]-ac Nf x[0][0][1][1][0][t] x[6][2][0][1][1][t]+dc x[6][2][1][1][1][t]

dx[6][2][0][1][2][t]/dt=ar Nf x[0][2][0][1][2][t] x[6][0][0][1][0][t]+nl x[6][2][0][0][2][t]+bbin Nf x[0][0][0][1][2][t] x[6][2][0][1][0][t]-dr x[6][2][0][1][2][t]-unbbin x[6][2][0][1][2][t]-ac Nf x[0][0][1][1][0][t] x[6][2][0][1][2][t]+dc x[6][2][1][1][2][t]

dx[6][2][1][0][0][t]/dt=ar x[0][2][0][0][0][t] x[6][0][1][0][0][t]+ac x[0][0][1][0][0][t] x[6][2][0][0][0][t]-dc x[6][2][1][0][0][t]-dr x[6][2][1][0][0][t]-nl x[6][2][1][0][0][t]-bbin x[0][0][0][0][1][t] x[6][2][1][0][0][t]-bbin x[0][0][0][0][2][t] x[6][2][1][0][0][t]+unbbin x[6][2][1][0][1][t]+unbbin x[6][2][1][0][2][t]+ne x[6][2][1][1][0][t]

dx[6][2][1][0][1][t]/dt=ar x[0][2][0][0][1][t] x[6][0][1][0][0][t]+ac x[0][0][1][0][0][t] x[6][2][0][0][1][t]+bbin x[0][0][0][0][1][t] x[6][2][1][0][0][t]-dc x[6][2][1][0][1][t]-dr x[6][2][1][0][1][t]-nl x[6][2][1][0][1][t]-unbbin x[6][2][1][0][1][t]

dx[6][2][1][0][2][t]/dt=ar x[0][2][0][0][2][t] x[6][0][1][0][0][t]+ac x[0][0][1][0][0][t] x[6][2][0][0][2][t]+bbin x[0][0][0][0][2][t] x[6][2][1][0][0][t]-dc x[6][2][1][0][2][t]-dr x[6][2][1][0][2][t]-nl x[6][2][1][0][2][t]-unbbin x[6][2][1][0][2][t]

dx[6][2][1][1][0][t]/dt=ar Nf x[0][2][0][1][0][t] x[6][0][1][1][0][t]+ac Nf x[0][0][1][1][0][t] x[6][2][0][1][0][t]+nl x[6][2][1][0][0][t]-dc x[6][2][1][1][0][t]-dr x[6][2][1][1][0][t]-ne x[6][2][1][1][0][t]-bbin Nf x[0][0][0][1][1][t] x[6][2][1][1][0][t]-bbin Nf x[0][0][0][1][2][t] x[6][2][1][1][0][t]+unbbin x[6][2][1][1][1][t]+unbbin x[6][2][1][1][2][t]

dx[6][2][1][1][1][t]/dt=ar Nf x[0][2][0][1][1][t] x[6][0][1][1][0][t]+ac Nf x[0][0][1][1][0][t] x[6][2][0][1][1][t]+nl x[6][2][1][0][1][t]+bbin Nf x[0][0][0][1][1][t] x[6][2][1][1][0][t]-dc x[6][2][1][1][1][t]-dr x[6][2][1][1][1][t]-unbbin x[6][2][1][1][1][t]

dx[6][2][1][1][2][t]/dt=ar Nf x[0][2][0][1][2][t] x[6][0][1][1][0][t]+ac Nf x[0][0][1][1][0][t] x[6][2][0][1][2][t]+nl x[6][2][1][0][2][t]+bbin Nf x[0][0][0][1][2][t] x[6][2][1][1][0][t]-dc x[6][2][1][1][2][t]-dr x[6][2][1][1][2][t]-unbbin x[6][2][1][1][2][t]
