# Supplementary material for: Emergence of Noise-Induced Oscillations in the Central Circadian Pacemaker
Source: PLoS Biol. 2010 Oct 12;8(10):e1000513. doi: 10.1371/journal.pbio.1000513 (PMC2953532; doi:10.1371/journal.pbio.1000513)
Supplement: Text S1 — Supplementary methods. (0.06 MB DOC) [file pbio.1000513.s016.doc]

Supplementary Methods:

**Emergence of noise-induced oscillations**

**in the central circadian pacemaker**

**Caroline H. Ko^1,2,3,#,**^, Yujiro R. Yamada^4,#^, David K. Welsh^5,6,7,#^,**

**Ethan D. Buhr2,^, Andrew C. Liu^8,+^, Eric E. Zhang^8^, Martin R. Ralph^3,9^,**

**Steve A. Kay^5^, Daniel B. Forger^4,10^, Joseph S. Takahashi^1,2,11*^**

^1^ Department of Neuroscience, UT Southwestern Medical Center, Dallas, TX, USA.

^2^ Department of Neurobiology and Physiology, Northwestern University, Evanston, IL, USA.

^3^ Department of Psychology, University of Toronto, ON, Canada.

^4^ Department of Mathematics, University of Michigan, Ann Arbor, MI, USA.

^5^ Department of Cell and Developmental Biology, and

^6^ Department of Psychiatry, University of California, San Diego, CA, USA.

**^7^** Veterans Affairs San Diego Healthcare System, San Diego, CA USA.

^8^ Genomics Institute of Novartis Research Foundation, San Diego, CA, USA

^9^ Center for Biological Timing and Cognition, University of Toronto, ON, Canada.

^10^ Center for Computational Medicine and Bioinformatics, University of Michigan, Ann Arbor, MI, USA.

^11^ Howard Hughes Medical Institute, UT Southwestern Medical Center, Dallas, TX, USA.

* Correspondence should be addressed to:

Joseph S. Takahashi, Ph.D.

Department of Neuroscience

University of Texas Southwestern Medical Center

5323 Harry Hines Blvd., NA4.118

Dallas, TX 75390-9111

214-648-1876 (admin)

[Joseph.Takahashi@UTSouthwestern.edu](mailto:Joseph.Takahashi@UTSouthwestern.edu)

^#^ These authors contributed equally to this work.

^**^ Current address: Department of Chemistry, Northwestern University, Evanston, IL, 60622, USA.

^ Current address: Department of Ophthalmology, University of Washington, Seattle, WA, 98195, USA.

^+^ Current address: Department of Biology, University of Memphis, Memphis, TN, 38152, USA.

**Supplemental Methods**

**Fibroblast cell culture and DNA constructs**

Primary mouse fibroblasts were generated from tail samples by a standard enzymatic digestion procedure. Fibroblasts that spontaneously overcame replicative senescence (immortalization) were used. All fibroblasts were cultured in DMEM supplemented with 10% fetal bovine serum and antibiotics. They were grown to confluence prior to bioluminescence recording.

Transient transfection of 293T cells and Luciferase reporter assay for CRY1 repression were performed using Dual Light kit (Tropix) in triplicate wells of 96-well plates as described previously [81].

For quantitative PCR (Q-PCR), cyber green (ABI) was used according to manufacturer’s instructions. Primers used in the PCR were:

Bmal1F – GGACTTCGCCTCTACCTGTTCA;

Bmal1R – AACCATGTGCGAGTGCAGGCGC;

Bmal2F – TCTATGATCCCTCCACACATCCCCA; and,

Bmal2R – CGCATCCAACCACAAACAGGAAGC.

The primers were designed and tested to have consistent amplification efficiency for both WT and *Bmal1*^−/−^ template DNA.

**ADDITIONAL SUPPORTING INFORMATION FILES**

**Video S1.** Bioluminescence expression patterns from *Bmal1*^−/−^ SCN explants

**Video S2.** Bioluminescence expression patterns from *Bmal1*^−/−^ dissociated SCN neurons

**Protocol S1.** Code for the stochastic mathematical simulation of the circadian molecular clock; the code is written in the C programming language. This code was used to generate simulations found in Figures 3B, 4A, 4D, 4E, 5B, 5C, 5D, 5F, 6D, 8C, S3 and S4. The parameters, and a brief description, used in this code can be found in Figure S7.

**Protocol S2.** Description of model equations in human readable format. The equations for this model, parameters, and a brief description can be found in Figure S7. The equations were simulated in MatlLab and were used to generate Figures 3D, 4B, 4C, 5D, 5E, and 5F.

**Dataset S1**. Bioluminescence data from 14 individual WT SCN explants.

**Dataset S2**. Bioluminescence data from 16 individual *Bmal1^-/-^* SCN explants.

**Dataset S3**. Bioluminescence data from 40 individual *Bmal1^-/-^* SCN cells in an intact SCN explant slice.

**Dataset S4**. Bioluminescence data from 40 individual *Bmal1^-/-^* SCN cells in an intact SCN explant slice.

**Dataset S5**. Bioluminescence data from 115 individual *Bmal1^-/-^* SCN cells dispersed in culture.

**Dataset S6**. Bioluminescence data from 128 individual *Bmal1^-/-^* SCN cells dispersed in culture.
